# Comparative Transcriptomics of the Saprobic and Parasitic Growth Phases in *Coccidioides* spp

**DOI:** 10.1371/journal.pone.0041034

**Published:** 2012-07-20

**Authors:** Emily Whiston, Hua Zhang Wise, Thomas J. Sharpton, Ginger Jui, Garry T. Cole, John W. Taylor

**Affiliations:** 1 Department of Plant and Microbial Biology, University of California, Berkeley, California, United States of America; 2 Department of Biology, The University of Texas at San Antonio, San Antonio, Texas, United States of America; 3 The J. David Gladstone Institutes, University of California San Francisco, San Francisco, California, United States of America; Yonsei University, Republic of Korea

## Abstract

*Coccidioides immitis* and *C. posadasii*, the causative agents of coccidioidomycosis, are dimorphic fungal pathogens, which grow as hyphae in the saprobic phase in the environment and as spherules in the parasitic phase in the mammalian host. In this study, we use comparative transcriptomics to identify gene expression differences between the saprobic and parasitic growth phases. We prepared Illumina mRNA sequencing libraries for saprobic-phase hyphae and parasitic-phase spherules *in vitro* for *C. immitis* isolate RS and *C. posadasii* isolate C735 in biological triplicate. Of 9,910 total predicted genes in *Coccidioides*, we observed 1,298 genes up-regulated in the saprobic phase of both *C. immitis* and *C. posadasii* and 1,880 genes up-regulated in the parasitic phase of both species. Comparing the saprobic and parasitic growth phases, we observed considerable differential expression of cell surface-associated genes, particularly chitin-related genes. We also observed differential expression of several virulence factors previously identified in *Coccidioides* and other dimorphic fungal pathogens. These included alpha (1,3) glucan synthase, SOWgp, and several genes in the urease pathway. Furthermore, we observed differential expression in many genes predicted to be under positive selection in two recent *Coccidioides* comparative genomics studies. These results highlight a number of genes that may be crucial to dimorphic phase-switching and virulence in *Coccidioides*. These observations will impact priorities for future genetics-based studies in *Coccidioides* and provide context for studies in other fungal pathogens.

## Introduction

The methods for transcriptional profiling have changed dramatically in recent years from microarray-based techniques to full transcriptome sequencing using next-generation sequencing (NGS) technologies. NGS offers many advantages over traditional microarrays, but the underlying principle of comparative transcriptomics remains the same: analysis of changes in gene expression between conditions can identify genes critical to cellular responses to environmental cues, morphological change and growth. In particular, transcriptional profiling has been used in many fungal pathogens to identify genes critical to growth in a host environment [1], [2], [3], [4].

*Coccidioides* spp. are dimorphic fungal pathogens that cause the mammalian disease coccidioidomycosis, also known as San Joaquin Valley Fever – a potentially fatal infection that can occur in healthy human adults [5]. Formerly considered a single species, we now know that there are two species of *Coccidioides*: *C. immitis* and *C. posadasii* [6]. *C. immitis* is distributed throughout central and southern California and has at least two populations; *C. posadasii* is distributed throughout Arizona, Texas, Mexico and parts of South America and harbors at least three populations 6,7. There are no discernable phenotypic differences in pathogenicity between the two species, although differences in salt tolerance and thermal tolerance have been observed [6], [8], [9].

**Figure 1:**
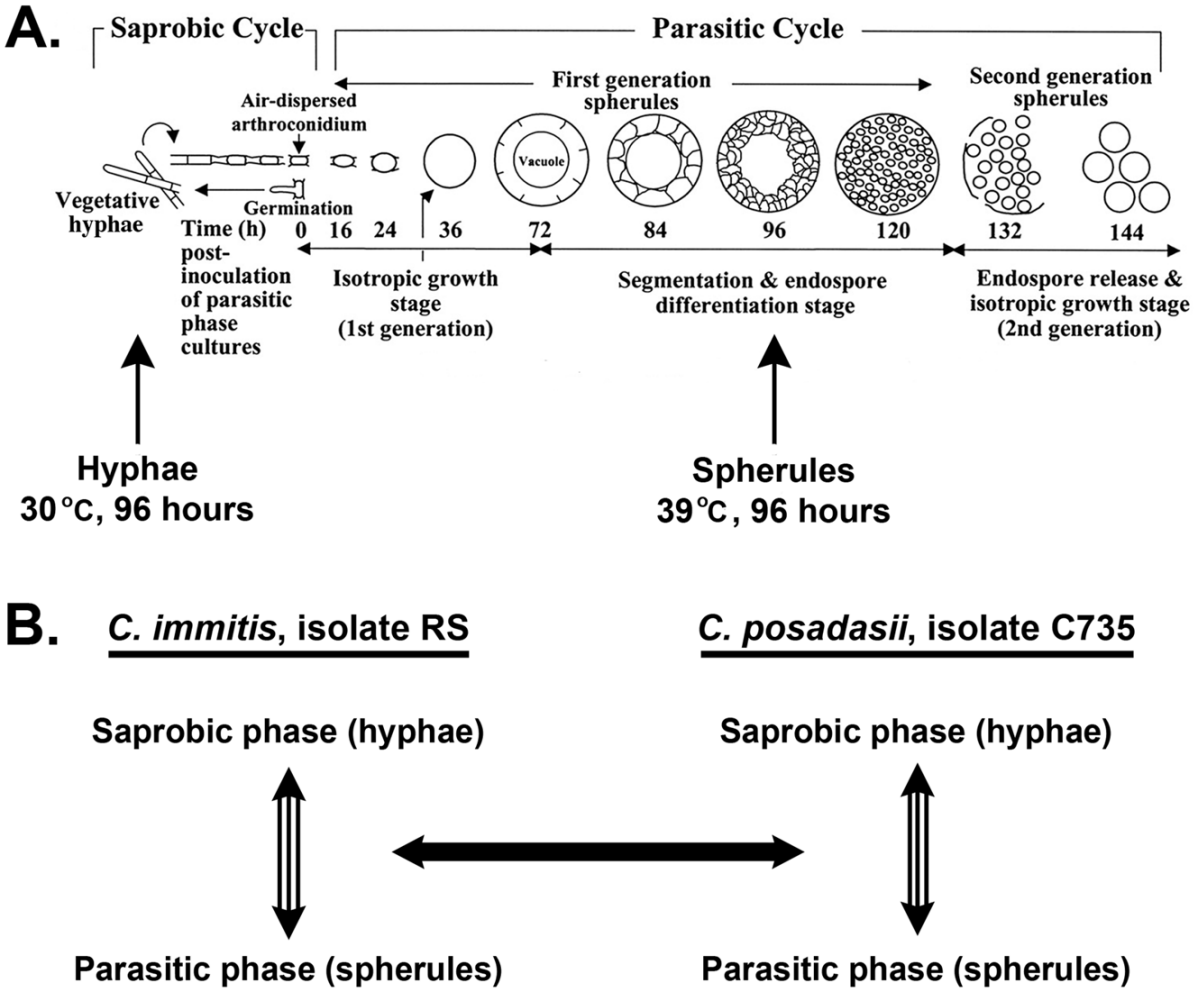
*Coccidioides* growth cycle and study overview. *Coccidioides* growth cycle in culture (**A**), total RNA was collected at 96 hours from hyphae and spherules, which were grown at 30°C and 39°C, respectively. Lifecycle in culture illustration adapted from Delgado *et al*, 2003 [11]. Samples were collected in biological triplicate and the results from *C. immitis* isolate RS and *C. posadasii* isolate C735 were compared (**B**).

*Coccidioides* spp. grow as mycelia in arid soil in association with dead mammals. Asexual reproduction occurs by production of arthroconidia, which are the infectious agents of disease that can cause pulmonary infection when inhaled by mammals. Unlike the other mammalian dimorphic fungal pathogens, which grow as yeast in the host, *Coccidioides* has a morphologically complex parasitic cycle [10], [11] (figure 1). Arthroconidia enlarge *in vivo* to form spherule initials that undergo isotropic growth to form mature spherules, within which nuclei divide and are packaged into hundreds of endospores that fill the maternal spherules. When a spherule ruptures due to continued isotropic growth, endospores are released and continue the cycle in the lungs or may enter the bloodstream and disseminate to almost any tissue and cause life-threatening secondary infections. This unique parasitic cycle distinguishes *Coccidioides* from other medically-important dimorphic fungal pathogens not only in growth morphology, but also in innate immune response because, unlike yeast cells, mature spherules are too large (60->100 µm in diameter) for mammalian immune cell phagocytosis [12]. Only one other dimorphic fungal pathogen, *Cryptococcus neoformans*, forms “giant cells” (up to 30 µm in diameter) that are too large to be phagocytosed by the host cells [13].

At least 150,000 people are infected with *Coccidioides* spp. annually in the United States; 40% of whom develop mild to severe pulmonary symptoms [12]. This number is likely an underestimate, judging from studies that estimate that 10–50% of people in endemic regions have been exposed to *Coccidioides* [14], including the southwestern U.S., which is home to over 30 million people. In an estimated 1–6% of clinically diagnosed cases of coccidioidomycosis, the initial pulmonary infection can disseminate to other soft tissues, including the brain, and cause secondary infections [12]. Disseminated disease is potentially fatal, even in healthy adults, although it is more common in children, the elderly and immune compromised patients. In children, the mortality rate of hospitalized patients is 1.5% [15]. Recent increases in symptomatic coccidioidomycosis cases have been reported in southern California (over 3-fold increase from 2000 to 2006) and Arizona (over 2-fold increase from 2001 to 2007) [9]. These local epidemics are likely correlated with drought associated with climate change in the Southwest, outbreaks associated with strong winds and other soil disturbances, and shifting population (ie: influx of immunologically naïve people from non-endemic areas) [9], [16]. Due to the potentially severe pathogenicity of *Coccidioides* spp., its ease of dissemination via air-borne spores, and the absence of a vaccine, *C. immitis* and *C. posadasii* are considered by the U.S. Department of Health and Human Services to be Select Agents with the potential for bioterrorism [17].

A previous study compared transcription levels in the saprobic and parasitic phases by microarray analysis of just 1,000 genes in two isolates of *C. posadasii* [18]. That study found that genes related to stress response and lipid metabolism were significantly up-regulated in the parasitic phase. Since that study, 20 full genomes have become available for *Coccidioides* spp.: 10 *C. immitis* and 10 *C. posadasii* [8], [19]. Of these sequenced isolates, *C. immitis* isolate RS is a finished genome with six contigs, each representing a whole chromosome. Together, the six chromosomes total 28.9 Mb and contain 9,910 genes. In this study, we use NGS to assess gene expression of all annotated genes in the saprobic and parasitic growth phases with the aim of identifying genes that are differentially expressed between the *Coccidioides* growth phases in both *C. immitis* and *C. posadasii*.

Amongst all of the genes differentially expressed between the saprobic and parasitic phases, we detected expression changes between the growth phases in stress response, cell wall remodeling, polar growth and transcription factors. We also specifically investigated gene expression of previously identified vaccine candidates [8], genes in introgressed regions [8], genes showing evidence of positive selection [19], known virulence factors, and other genes of interest from previous studies in *Coccidioides* and other dimorphic fungal pathogens. We also found genes with no predicted function that show strong differential expression between the *Coccidioides* growth phases. Based on Pfam domain predictions and sequence homology to proteins in other species, nearly 50% of the genes in *Coccidioides* have no predicted function or recognized functional domains. A priori, any of these approximately 4,500 genes could be important to pathogenicity. By identifying genes potentially important in dimorphic-switching and parasitic growth, transcriptional profiling of the saprobic and parasitic phases will prioritize future reverse genetics-based studies of proteins with no known function.

## Results

### Experimental Design

To compare gene expression in the saprobic and parasitic growth phases between the sibling *Coccidioides* species, *C. immitis* and *C. posadasii*, we focused our analyses on actively-growing hyphae (saprobic phase) and pre-endosporulation spherules (parasitic phase) cultured for 96 hours *in vitro* in their respective growth conditions as described in the methods. We chose *C. immitis* isolate RS and *C. posadasii* isolate C735 from a pool of 20 recently sequenced *Coccidioides* spp. isolates [8] because they represent the best assembled genomes for *C. immitis* and *C. posadasii* respectively. We chose the 96 hour time-point because at this stage in culture, saprobic-phase hyphae are in the exponential phase of filamentation and the parasitic phase is in near-synchrony – spherules are segmented and in the early stage of endospore differentiation (figure 1a). To find genes that were significantly differentially expressed between the two growth phases, we first assessed gene expression within species and then compared gene sets between species (figure 1b).

### Library Summary

**Figure 2:**
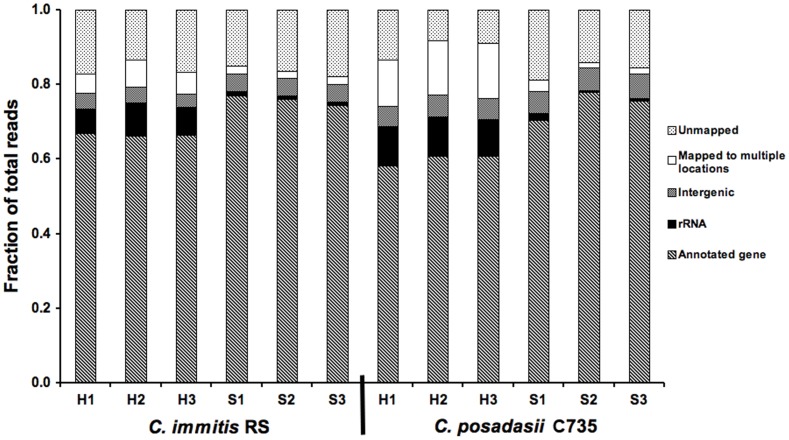
Library mapping summary. Per-lane read proportions for all libraries. H: hyphae (saprobic phase), S: spherule (parasitic phase).

Three RNAseq libraries were prepared and sequenced for each of the two species and two growth phases. From these 12 RNAseq libraries, the mean number of total 36 base pair Illumina reads was 12.1×10^6^±4.1×10^6^. Across all libraries, a mean of 85.4% of reads mapped to the genome. Reads that did not map include adapter-dimers and reads with base-calling errors. Of the mapped reads, a mean of 11.3% in parasitic-phase libraries and 2.5% in saprobic-phase libraries (p = 0.001) mapped to multiple locations in the genome and were therefore not assigned or considered in gene expression statistical analyses; a mean of 80% of these unassigned reads mapped to predicted repetitive elements. Of the mapped reads that were assigned, reads mapping to predicted genes accounted for a mean of 81.9% in saprobic-phase libraries and 92.1% in parasitic-phase libraries (p<0.0001) (figure 2). Although the library preparation protocol includes an mRNA pull-down, not all of the rRNA was removed from the samples because assigned reads mapping to ribosomal RNA sequence accounted for a mean of 11.8% in saprobic-phase libraries and 1.3% in parasitic-phase libraries (p<0.0001). The high level of mRNA reads in saprobic-phase samples accounts for the disparity in the percentage of reads mapping to predicted genes between the parasitic and saprobic-phase libraries. Finally, assigned reads mapping to intergenic regions that included UTRs and non-predicted genes accounted for a mean of 6.3% in saprobic-phase and 6.4% in parasitic-phase libraries.

**Figure 3:**
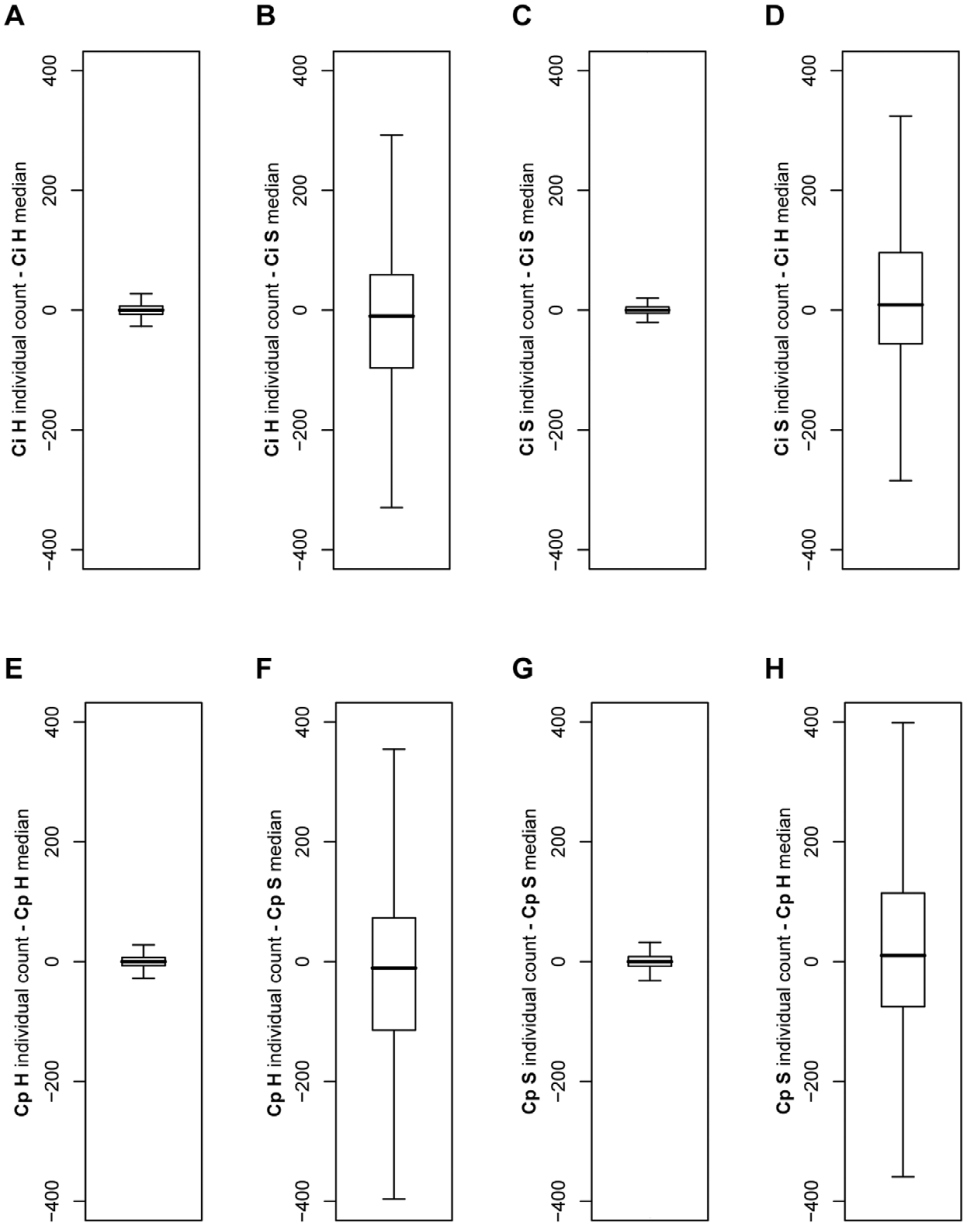
Median-difference boxplots. Median-difference boxplots showing the interquartile range (box with median line) of individual counts from the median count for all genes. Plot “whiskers” extend 1.5 times the interquartile range; outlier points are not shown. Ci: *C. immitis*, Cp: *C. posadasii*, H: hyphae (saprobic phase), S: spherule (parasitic phase).

As expected, we observed far greater expression differences between conditions than between biological replicates within conditions. To assess reproducibility across biological replicates, median-difference plots were used to compare the libraries within and between biological replicates (Figure 3). These plots show that gene expression levels among biological replicates for the same condition are much more similar to each other than biological replicates between conditions, indicating that our results are reproducible and that there is a strong difference in gene expression between the saprobic and parasitic growth phases.

### Genes Showing Higher Expression Levels in the Saprobic Phase (Hyphal Growth)

**Figure 4:**
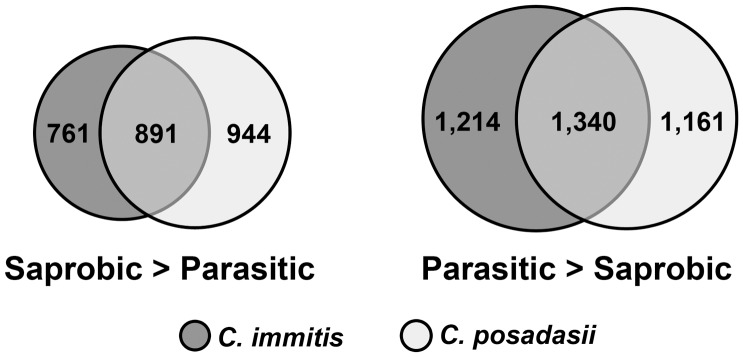
Differentially expressed genes in *C. immitis* and *C. posadasii*. Venn diagrams showing the number of genes commonly differentially regulated in the saprobic vs. parasitic growth phases of *C. immitis* and *C. posadasii*.

In *C. immitis* isolate RS, 2,303 genes showed a significantly higher level of expression (up-regulated) in the saprobic phase compared to the parasitic phase. In *C. posadasii* isolate C735, 2,177 genes showed a significantly higher level of expression (up-regulated) in the saprobic phase compared to the parasitic phase. Comparing these two gene sets, 1,298 genes were significantly up-regulated in the saprobic phase in both species (figure 4a, Table S1). The 15 genes most strongly up-regulated in the saprobic phase in both *Coccidioides* spp. are shown in table 1. Of these, 8 are predicted to be secreted proteins.

**Table 1:** Top 15 genes with significantly higher expression (up-regulated) in the saprobic phase.

Fold difference*	Annotation	Gene ID
185	Conserved protein (insect antifreeze protein repeat domain, predicted secreted)	CIMG_00925
166	Acetyltransferase	CIMG_07556
106	Acetamidase	CIMG_02374
101	Conserved hypothetical protein (predicted secreted)	CIMG_03870
94	Fungal hydrophobin (predicted secreted)	CIMG_06615
75	Conserved protein (PAN domain, predicted secreted)	CIMG_09824
53	Conserved protein (zinc-finger domain)	CIMG_00099
53	Conserved hypothetical protein	CIMG_06344
43	Putative serine proteinase	CIMG_09304
41	Cell wall synthesis protein (beta-glucosidase domain, SUN family, predicted secreted)	CIMG_05254
34	Hypothetical protein (predicted secreted)	CIMG_07839
31	Hypothetical protein	CIMG_13374
31	Helix-loop-helix transcription factor	CIMG_02390
29	Conserved hypothetical protein (pyridine nucleotide-disulphide oxidoreductase domain, predicted secreted)	CIMG_07557
24	Prp4 (CRoW domain-containing protein, predicted secreted)	CIMG_07303

* Fold difference is the mean saprobic/parasitic-phase expression level in *C. immitis* and *C. posadasii*.

In a functional enrichment test, 24 Gene Ontology (GO) terms were significantly enriched in the 1,298 genes up-regulated in the saprobic phase in both *C. immitis* and *C. posadasii* (Table S3). These included: 8 terms associated with mitosis/cytokinesis; 4 associated with nuclear export; 3 associated with budding; 3 associated with cytoskeleton; and the unique terms calmodulin binding, conjugation with cellular fusion, ergosterol biosynthesis, establishment of cell polarity, isoprenoid biosynthesis, and peroxisomal membrane.

### Genes Showing Higher Expression Levels in the Parasitic Phase (Spherule Growth)

In *C. immitis* isolate RS, 3,394 genes showed a significantly higher level of expression (up-regulated) in the parasitic phase compared to the saprobic phase. In *C. posadasii* isolate C735, 2,865 genes showed a significantly higher level of expression (up-regulated) in the parasitic phase compared to the saprobic phase. Comparing these gene sets, 1,880 genes were up-regulated in the parasitic phase in both species (figure 4b, Table S2). The 15 genes most strongly up-regulated in the parasitic phase in *Coccidioides* spp. are shown in table 2. Of these, two are predicted secreted proteins and two other genes are also involved in cell wall structure, sphingosine hydroxylase (CIMG_01209) and polysaccharide deacetylase (CIMG_02628). Of the top 15 genes up-regulated in the parasitic phase, 10 are hypothetical proteins with no inferred function.

**Table 2:** Top 15 genes with significantly higher expression (up-regulated) in the parasitic phase.

Fold difference*	Annotation	Gene ID
125	Conserved hypothetical protein (DUF 536)	CIMG_09539
68	Heat shock protein 30 (Hsp20/alpha-crystallin domain)	CIMG_01749
31	Conserved hypothetical protein	CIMG_12822
30	Conserved hypothetical protein (YCII-related domain)	CIMG_07089
29	Conserved hypothetical protein	CIMG_13084
28	Hypothetical protein	CIMG_11522
28	Hypothetical protein	CIMG_05235
26	Polysaccharide deacetylase (Arp2/3 complex subunit Arc16)	CIMG_02628
24	Conserved hypothetical protein (predicted secreted)	CIMG_00509
23	Hypothetical protein	CIMG_11203
19	Spherule outer-wall glycoprotein (SOWgp, predicted secreted)	CIMG_04613
18	Conserved hypothetical protein	CIMG_10488
18	Hypothetical protein	CIMG_10670
17	Sphingosine hydroxylase	CIMG_01209
17	Conserved hypothetical protein	CIMG_04740

* Fold difference is the mean parasitic/saprobic-phase expression level in *C. immitis* and *C. posadasii*.

In a functional enrichment test, 3 GO terms were significantly enriched in the 1,880 genes up-regulated in the parasitic phase in both *C. immitis* and *C. posadasii* (Table S3). These were: response to light, lipid particle, and oxidoreductase activity.

### Specific Genes of Interest

In addition to searching for genes that might be important to the parasitic phase based on a significant change in their expression between the two growth phases, we used our data to assess gene expression for genes identified in functional and bioinformatics studies from *Coccidioides* and other fungal dimorphic pathogens (tables 3–4). These include genes important to parasitism, virulence, the regulation of dimorphism, protective immune response (vaccine candidates), cell-surface tagged proteins, and genes previously found to be differentially expressed in *Coccidioides posadasii* by microarray [18].

**Table 3:** Genes of interest from previous studies.

Category	Gene name	Group of study(reference(s))	*Coccidioides*Gene ID	*C. immitis*exp. ratio*	*C. posadasii*exp. ratio*
**Dimorphism regulation**	4-hydroxyl-phenyl pyruvate dioxygenase(*4-HPPD*)	*Paracoccidioides* [24], [25]	CIMG_01466	3.52**	4.90**
	*DRK1*	Multiple [21], [62]	CIMG_04512	0.87	0.59**
	Ornithine decarboxylase (*ODC*)	*Coccidioides* [23]	CIMG_08778	0.84	1.92**
	*RYP1* (*WOR1, TOS9*)	Multiple [21], [22]	CIMG_02671	0.91	1.20**
	*RYP2*	*Histoplasma* [20]	CIMG_01530	0.61	0.63
	*RYP3*	*Histoplasma* [20]	CIMG_09962	0.38**	0.32**
**Virulence Factors**	Alpha (1,3) glucan synthase (*AGS1*)	Multiple [26], [27], [28], [29]	CIMG_13256	1.93**	1.59**
	1,3-beta-glucanosyltransferase	*Coccidioides* [25]	CIMG_00181	1.06	3.64**
	Metalloprotease 1 (*MEP1*)	*Coccidioides* [32]	CIMG_08674	0.34**	2.04**
	Urease	*Coccidioides* [37]	CIMG_04935	1.60	2.38**
	Urease accessory protein	*Coccidioides*	CIMG_05165	1.46**	0.76
	Ureidoglycolate hydrolase (*UGH*)	*Coccidioides*	CIMG_02178	1.89**	3.48**
**Cellular processes**	*GAC1*	*Saccharomyces*	CIMG_05377	6.62**	18.18**
	*GLC7*	*Saccharomyces*	CIMG_04906	0.63**	0.55**
	*SEC1*	*Saccharomyces* [33], [34]	CIMG_00724	1.94**	1.72**

* Expression ratio: parasitic/saprobic expression. Ratios >1.0 indicate higher expression (up-regulation) in the parasitic phase and ratios <1.0 indicate higher expression (up-regulation) in the saprobic phase.

** p-value <0.05.

**Table 4:** mRNA expression levels of previously identified vaccine candidates [8].

Category	Gene ID	Annotation	*C. immitis* expression*	*C. posadasii* expression*	Differential regulation**
**Proline-rich proteins**	CIMG_09696	Prp1	35,414	55,176	No
	CIMG_09560	Prp2	149	129	No
	CIMG_02492	Prp3	124	666	No
	CIMG_07303	Prp4	62	46	Up H (3.9-fold)
	CIMG_05560	Prp5	170	108	No
	CIMG_07843	Prp6	384	485	No
	CIMG_09029	Prp7	39	61	No
	CIMG_02073	Prp8	241	223	Up S (2.2-fold)
**Low polymorph.,** **High T-cell epitope**	CIMG_00642	Conserved hypothetical protein (putative COPI-associated protein)	941	651	No
	CIMG_02599	Conserved hypothetical protein (DUF2015)	2,822	3,078	No
	CIMG_03437	Conserved hypothetical protein	883	-	No
	CIMG_04746	Conserved hypothetical protein	902	664	No
	CIMG_04894	Conserved hypothetical protein	814	1,168	No
	CIMG_07187	Iron/copper transporter Atx1	724	776	No
	CIMG_07738	Conserved hypothetical protein	384	325	Up H (15-fold)
	CIMG_08533	Golgi membrane protein YIPF5	800	548	No
	CIMG_09101	Unfolded protein response protein Orm1	103	74	No
	CIMG_10953	Conserved hypothetical protein	1,929	1,327	No
	CIMG_11035	Conserved hypothetical protein	814	1,378	No
	CIMG_12464	Integral membrane protein	746	306	No

* Parasitic phase expression levels are normalized by library size and gene length.

** Differential regulation: Up H: up-regulated in hyphae (saprobic phase), Up S: up-regulated in spherules (parasitic phase).

Regulation of dimorphism has been well-studied in the mammalian dimorphic pathogen *Histoplasma capsulatum*; the master regulators Drk1, Ryp1, Ryp2 and Ryp3 are all up-regulated in the parasitic yeast phase and are critical for yeast-phase growth [20], [21]. The Ryp1 homolog in *Candida albicans* (Wor1) is a white-opaque transition master regulator [22]. None of the homologs of these *Histoplasma* dimorphism regulators (Ryp1, Ryp2, Ryp3 and Drk1) are up-regulated in the parasitic spherule phase in *Coccidioides* spp (table 3). Curiously, the *Coccidioides* Ryp3 homolog is up-regulated in hyphae (table 3) and may therefore play a role in hyphal phase growth in *Coccidioides*. Another gene, ODC (CIMG_08778), which was previously implicated as a spherule phase regulator in *Coccidioides* [23] shows differential expression only in *C. posadasii* in this study (table 3). One gene potentially involved in *Paracoccidioides* dimorphism regulation, 4-hydroxyl-phenyl pyruvate dioxygenase (4-HPPD, CIMG_01466) [24], [25], is up-regulated in the spherule phase of both *C. immitis* and *C. posadasii* (table 3).

Several virulence factors previously identified in *Coccidioides* and other dimorphic fungal pathogens [25] are up-regulated in the parasitic phase: *AGS1* (CIMG_13256), *SOWgp* (CIMG_00181), and *UGH* (CIMG_02178). Loss of *AGS1* in *Histoplasma* and other fungal pathogens has been shown to decrease virulence [26], [27], [28], [29], [30]. SOWgp is a known *Coccidioides* virulence factor [10], [31]. UGH has been shown to be critical to *Coccidioides* virulence (Hua Zhang, personal communication). Other virulence factors did not show the expected expression patterns and may indicate isolate and species differences in gene expression that affect virulence. Mep1 was previously shown in *C. posadasii* to degrade SOWgp on endospore walls and helps prevent immune detection of small endospores [32]. *MEP1* (CIMG_08674) is up-regulated in the parasitic phase in *C. posadasii* but shows the opposite expression in *C. immitis* (up-regulated in the saprobic phase). Other genes that are differentially regulated either in *C. immitis* or in *C. posadasii* (but not both) include urease, urease accessory protein, 1,3-beta-glucanosyltransferase, and arginase.

Homologs of several well-studied genes involved in cellular processes are differentially regulated in *Coccidioides*, including *GAC1*, *GLC7* and *SEC1* (Table 3). *GAC1* is up-regulated in the parasitic phase; its gene product is a regulator of Glc7, the catalytic subunit of protein phosphatase type 1; surprisingly, unlike *GAC1*, *GLC7* is up-regulated in the saprobic phase. Sec1 is involved in vesicle trafficking and secretion (SNARE regulation) [33], [34] and is up-regulated in the parasitic phase.

Chitin is a critical component of the fungal cell wall and several predicted chitin-associated genes are differentially regulated in the saprobic and parasitic phases of *Coccidioides*. Of 21 total chitin-related genes predicted in *Coccidioides immitis* isolate RS, 8 were significantly differentially expressed in both *C. immitis* and *C. posadasii*. Of these, 5 were up-regulated in the saprobic phase; these were chitin synthase 2 (3.7-fold, CIMG_08655), class III chitin synthase (4.1-fold, CIMG_05647), a chitin synthase activator (1.9-fold, CIMG_08769), chitinase 1 (28-fold, CIMG_02795), and chitinase 3 (41-fold, CIMG_02860). Of the 8 chitin-related genes differentially regulated in both *C. immitis* and *C. posadasii*, 3 were up-regulated in the parasitic phase; these were chitinase 2 (4.5-fold, CIMG_00348), a chitin synthase activator (2-fold, CIMG_10086), and chitinase 7 (3-fold, CIMG_03822). These results are consistent with the considerable cell wall restructuring that must occur during dimorphic phase-switching.

A recent phylo-genomics study of *Coccidioides* spp. and other sequenced species in the Eurotiomycetes identified 50 genes that show evidence of positive selection between *C. immitis* and *C. posadasii*. Of these, 11 genes were up-regulated in the parasitic phase in both *C. immitis* and *C. posadasii* (Table S2) and 13 were up-regulated in the saprobic phase in both species (Table S1). An additional five genes that show evidence of positive selection were significantly differentially in both species, but showed opposing gene expression patterns in *C. immitis* and *C. posadasii*; these were: trimethyllysine dioxygenase (CIMG_03536), two hypothetical proteins (CIMG_04115 and CIMG_05894), a RhoGEF domain-containing protein (CIMG_07534), and a fungal Zn binuclear cluster domain-containing protein (CIMG_10021). The remaining 21 genes with evidence of positive selection showed no significant differences in transcription between the saprobic and parasitic phases. This study also identified 92 genes that appear to be individual gene gains in *Coccidioides* spp. Of these, 22 genes were up-regulated in the parasitic phase in both *C. immitis* and *C. posadasii* (Table S2) and 8 were up-regulated in the saprobic phase in both species (Table S1).

In a recent *Coccidioides* population genomics study, one of the most interesting findings was evidence of introgression between *C. immitis* and *C. posadasii* [8]. Of 70 such regions of introgression, one region with a highly conserved boundary was of particular interest. There are two genes found at the conserved boundary of this introgressed region; one showed extremely low levels of expression in all conditions (*MEP4,* CIMG_00508), while the other was strongly up-regulated in the parasitic phase in both *C. immitis* and *C. posadasii* (CIMG_00509, table 2). The population genomics study also identified 20 genes as potential vaccine candidates [8]. Of these, all showed detectable expression in both the parasitic and saprobic phases; 2 were significantly up-regulated in the saprobic phase and 1 was significantly up-regulated in the parasitic phase (Table 4).

We also investigated expression of genes previously found to be significantly different between *C. posadasii* saprobic phase and two stages of parasitic phase development (36 and 136 hours) in a microarray study of 1000 genes [18]. These two periods of incubation of the parasitic phase corresponded with the differentiation of pre-segmented and endospoulating spherules, respectively. In that study, 27 genes were up-regulated in the saprobic phase (compared to both parasitic phase time-points); in this study, 14 of those genes are significantly up-regulated in the saprobic phase, while 1 is significantly up-regulated in the parasitic phase (Tables S1, S2). The previous study also found 65 genes that were up-regulated in both parasitic phase time-points (compared to the saprobic); in this study, 21 of those genes are significantly up-regulated in the parasitic phase, while 8 are significantly up-regulated in the saprobic phase (Tables S1, S2).

## Discussion

With 33% of the total number of annotated genes in *Coccidioides* spp. differentially expressed between the saprobic and parasitic growth phases in this study, we have focused on genes of particular biological interest. These included genes related to the cell wall, cellular processes, vesicle trafficking, regulation of dimorphism, virulence, protective immune response (vaccine candidates), and genes of unknown function. We were also keenly interested in how these results compare to previous studies in *Coccidioides* spp. and other dimorphic fungal pathogens.

### Cell Wall-associated Proteins

Given the major differences in growth and cell morphology between the saprobic (polar growth as hyphae) and parasitic (isotropic growth as spherules) phases of *Coccidioides* spp., we expected to observe considerable differences in expression of genes that encode cell wall-associated and secreted proteins. Of the 15 genes most strongly up-regulated in the saprobic phase of both *C. immitis* and *C. posadasii*, 8 were predicted to be secreted proteins and may therefore be particularly important in the hyphal cell wall or cell surface. Additionally, the Gene Ontology (GO) term ergosterol biosynthesis was significantly over-represented in a functional enrichment test for all genes up-regulated in the saprobic phase; ergosterol is a major component of fungal cell membranes.

Cell wall-associated and secreted proteins were also common in the genes up-regulated in the parasitic phase of both *C. immtis* and *C. posadasii*. Of the 15 genes most strongly up-regulated in the parasitic phase, four were predicted secreted or cell-wall associated. Of these, CIMG_00509 is of particular interest even though it is a short peptide (99 amino acids in length) with no predicted function. It is interesting because this gene is unique to *Coccidioides* [19] and it lies just inside the conserved boundary of a region introgressed from *C. posadasii* to *C. immitis* [8]. Such regions are thought to spread through recipient populations by introgression following hybridization because genes within the region are positively selected [8]. CIMG_00509 was overlooked in a population genomics study [8] because it lies next to the gene encoding metalloprotease 4 (*MEP4*, CIMG_00508). However, *MEP4* has a very low level of expression in both hyphae and spherule samples (<25 total read counts in all libraries) whereas CIMG 00509 is up-regulated 24-fold in spherules. Based on these results, we argue that selection may be acting on CIMG_00509 and not *MEP4*, as previously thought.

In addition to CIMG_00509, three other genes of the top 15 most strongly up-regulated in the parasitic phase were predicted to be secreted or associated with the cell-wall and two others are associated with cell-wall structure. These include SOWgp, an immuno-reactive cell-surface antigen, which has been previously studied in *Coccidioides*. As confirmed in this study, spherule-specific expression of *SOWgp* in parasitic phase growth has been previously shown [10]. *SOWgp* mutant strains show reduced virulence in mice [10]. SOWgp has a highly variable repetitive region that may be involved in immune evasion [35].

In addition to other cell wall genes strongly differentially expressed, we were specifically interested in expression of chitin-associated genes, because chitin is a major component of the fungal cell wall and was previously proposed as an anti-fungal drug target [36]. We observed 8 chitin-associated genes differentially expressed in *Coccidioides* – some up-regulated in the saprobic and some in the parasitic phase. These results suggest that there is considerable restructuring of chitin between growth phases. Given the redundancy of chitin-related genes, single gene deletions may not yield distinctive phenotypes. To prioritize the order of deletion, it may be useful to begin with those that are differentially regulated.

### Virulence Factors

We were particularly interested in secreted virulence factors previously identified in *Coccidioides* and other mammalian dimorphic pathogens. One of the best-studied virulence factors in *Coccidioides* is urease, which is released by parasitic-phase spherules in the host. Urease hydrolyzes both pathogen and host-derived urea, which yields ammonia, resulting in a significant increase in pH [37]. Urease activity also elicits a strong inflammatory host response, which combined with localized alkalinity, causes local tissue damage and exacerbates the course of disease [37]. We found that several genes associated with the urease pathway were up-regulated in the parasitic phase in this study: urease and two arginases were up-regulated in *C. posadasii*, urease accessory protein was up-regulated in *C. immitis*, and ureidoglycolate hydrolase (UGH) was up-regulated in both species. Studies assessing the roles of urease and UGH in *Coccidioides* pathogenicity are in progress. Another virulence factor previously studied in *Coccidioides*, Mep1, was up-regulated in spherules in *C. posadasii* but showed the opposite expression in *C. immitis* (up-regulated in hyphae). Mep1, a metalloprotease, degrades SOWgp on endospores to prevent phagocytosis and was previously shown to be highly expressed at 132 hr but not at 96 hr in *C. posadasii* [32]. Mep1 expression likely fluctuates during the parasitic growth cycle and seems that regulation of this gene is different in *C. immitis* and *C. posadasii*, accounting for the opposing results observed.

We were also interested in virulence factors that have been studied in other dimorphic fungal pathogens. Many virulence factors are species-specific and not found in *Coccidioides*, such as the *Paracoccidioides* glycoproteins gp43 and gp70 [25]. The only homolog of a known virulence factor from another species that was up-regulated in the parasitic phase in both *C. immitis* and *C. posadasii* was *AGS1*; Ags1 synthesizes the cell-wall polysaccharide α-(1,3)-glucan and has been shown to contribute to virulence in the dimorphic fungal pathogens *Histoplasma, Blastomyces, Paracoccidioides* and the non-dimorphic pathogen *Aspergillus* [25], [26], [27], [28], [29], [30], [38]. These results suggest that virulence factors in *Coccidioides* and the other dimorphic fungal pathogens have evolved separately. It is worth noting that one of the virulence factors mentioned above, Mep1, is among the protease gene families with extreme gene family expansion in *Coccidioides* species, but not in *Histoplasma* [19], underscoring the apparent independent evolution of pathogenicity in these two dimorphic fungal pathogens.

### Vaccine Candidates

To help prioritize further research on candidate vaccine targets, we have examined expression of genes that encode proteins that may stimulate host cellular immunity against coccidioidomycosis. These putative vaccine candidates fall into 2 categories: a family of proline-rich proteins (Prp) (8 genes) and proteins likely to be immunoreactive judging from their high T-cell epitope density and low polymorphism (12 genes) [8]. All vaccine candidates showed detectable expression in the parasitic phase. Prp1 (also known as Ag2/PRA) and Prp2 have already been tested as single vaccine candidates, as well as in a combined vaccine [39]. The Prp1/Prp2 combination vaccine offered better protection than the single-protein vaccines, but was still unable to provide sterile immunity in mice [39]. The *PRP1* gene showed extremely high expression levels in both the saprobic and parasitic phases in both *C. immitis* and *C. posadasii*. Of the other 7 Prp genes, only *PRP8* was up-regulated in spherules in both species, although the relative expression was below the mean for all genes. Of the 12 genes with high epitope density and low polymorphism, four (CIMG_02599, CIMG_04894, CIMG_10953 and CIMG_11035) had very high relative expression in the parasitic phase of both species and should be prioritized for vaccine studies. Given the results of the Prp1/Prp2 vaccine study, a combination vaccine targeting multiple high-expression genes may be more successful than single gene target vaccines.

### Cellular Processes and Metabolism

Differential expression of genes related to cellular processes and metabolism may be important in dimorphic phase-switching and growth. Functional enrichment analysis of the differentially regulated gene sets highlights major growth differences between the saprobic and parasitic phases. Functional (GO-term) enrichment for all genes up-regulated in the saprobic phase in both *C. immitis* and *C. posadasii* showed that this gene set is, as expected, enriched for cellular functions associated with fungal hyphal growth – notably the functional terms budding, cytoskeleton, establishment of cell polarity, calmodulin binding and peroxisomal membrane. The peroxisomal membrane in filamentous fungi includes woronin bodies, which help to control leaks after hyphal damage by blocking septal pores [40]. The major gene required for woronin body formation is *HEX1* [40], [41]; the homolog of this gene (CIMG_06738) was up-regulated in hyphae (Table S2). It is highly unlikely that woronin bodies would be associated with non-hyphal fungal growth, such as parasitic-phase spherules. In addition to the ‘classic’ hyphal growth functional terms enriched, 8 terms associated with mitosis/cytokinesis were enriched in the saprobic phase up-regulated genes. The upregulation of many cell-cycle related genes in the saprobic phase is consistent with active nuclear and cell division in hyphae at 96 hours and its near absence in parasitic-phase spherules as early as 72 hours after their induction [42].

Curiously, the catalytic subunit of protein phosphatase type 1 (*GLC7*, CIMG_04906) was up-regulated in the saprobic phase of both species, while its regulatory subunit (*GAC1*, CIMG_05377) was up-regulated in the parasitic phase. Gac1 has been previously linked to ion homeostasis and glycogen accumulation [43] and its activity in parasitic-phase spherules may therefore be related to these functions in the host environment. The opposing regulation of *GLC7* and *GAC1* indicates that protein phosphatase type 1 may be critical to growth in both the saprobic and parasitic phases but may serve different functions.

### Vesicle Trafficking

Secondary metabolites secreted by vesicle trafficking pathways have previously been shown to be critical to virulence in other fungal pathogens, such as *Aspergillus* spp. [44]. The velvet complex, a global regulator of secondary metabolite production, includes the genes *VEA* (CIMG_06878, not differentially expressed), *VELB* (CIMG_09962, up-regulated in the saprobic phase in *C. immitis* and *C. posadasii*) and *LAEA* (CIMG_03247, up-regulated in the saprobic phase in *C. posadasii*). These results suggest that the velvet secondary metabolite pathway may not be important in *Coccidioides* virulence. VelB has been shown to be critical to spore development in *A. nidulans* [45], and may play a similar role in spore production by *Coccidioides* saprobic-phase hyphae. Interestingly, the homolog of vesicle-trafficking gene *SEC1* in *Coccidioides* (CIMG_00724) was up-regulated in the parasitic phase of *C. immitis* and *C. posadasii*. Vesicle trafficking via Sec1 may be critical to spherule growth – whether vesicles are involved in virulence, cellular growth processes or both.

### Regulation of Dimorphism

Regulation of dimorphism has been studied in numerous mammalian fungal pathogens. The best-studied dimorphism regulators are the Ryp genes in *Histoplasma capsulatum*. Ryp1, Ryp2 and Ryp3 are transcriptional regulators with pivotal roles in pathogenic yeast-phase growth and dimorphic phase-switching in *H. capsulatum* [20]; all three genes are up-regulated during yeast-phase growth. In this study, the *Coccidioides RYP1* homolog (CIMG_02671) was up-regulated in the parasitic phase in *C. posadasii*, but not differentially regulated in *C. immitis*; the *RYP2* homolog (CIMG_01530) was not differentially expressed in either species; and the *RYP3* homolog was up-regulated in the saprobic phase of both *C. immitis* and *C. posadasii*. These results suggest that regulation of the parasitic phase in *Coccidioides* is different from that of *Histoplasma*. However, further sampling of additional parasitic-phase growth time-points, particularly early time-points during the spore-to-spherule transition, is necessary before concluding that the above genes are not involved in parasitic-phase growth.

Although the *H. capsulatum* dimorphism master regulators did not show similar results here, a homolog of a gene thought to be involved in *Paracoccidioides* dimorphism was up-regulated in the parasitic phase. The protein 4-HPPD is involved in aromatic amino acid catabolism and is up-regulated during the mycelium-to-yeast transition in *Paracoccidioides* [24], [25]. Chemical inhibition of 4-HPPD prevents *Paracoccidioides* transition to parasitic yeast phase [24]. The homolog of *4-HPPD* in *Coccidioides* (CIMG_01466) was up-regulated in the parasitic phase of both *C. immitis* and *C. posadasii* and may therefore play a similar role in *Coccidioides* parasitic-phase growth. Interestingly, the *Coccidioides* 4-HPPD protein has been previously shown to elicit a specific T-cell immune response [46], [47].

### Genes under Positive Selection

Based on a genome-wide comparison of synonymous and non-synonymous nucleic acid substitutions between *C. immitis* isolate RS and *C. posdasii* isolate C735, 50 genes showed evidence of positive selection [19]. Of these, 13 genes were up-regulated in the saprobic phase of both *Coccidioides* species. Positive selection in these genes could be related to adaptation to the different environments, both physical parameters and differences in the local small mammal hosts. The saprobic phase up-regulated genes under positive selection included a chitin synthase activator and the transcription factor HacA, which is related to unfolded protein response [48]. Another saprobic-phase up-regulated gene under positive selection, a predicted O-methyltransferase, is unique to *Coccidioides* [19] and may be important to saprobic-phase gene regulation. We also observed 11 genes up-regulated in the parasitic phase that appear to be under positive selection, again likely related to adaptation to the physical and biological environment – including potential differences in immune response to infection between local small mammal hosts. Furthermore, we observed 5 genes that showed opposing expression patterns in *C. immitis* and *C. posadasii*; positive selection in these genes may indicate reciprocal adaptation by functional divergence following speciation.

### Genes of Unknown Function

Genes of unknown function may be important in *Coccidioides* virulence and dimorphic growth. With few exceptions, gene functions in *Coccidioides* have been inferred from functional domain predictions and homology to genes studied in other organisms. Of all *Coccidioides* genes, 40% have at least one associated functional GO term, compared to 47% of the saprobic-phase up-regulated genes and just 29% of the parasitic-phase up-regulated genes. The GO terms used in this study were derived from homology with *Saccharomyces cerevisiae*, *S. pombe* and *Neurospora* spp. As the spherule morphology is unique to *Coccidioides*, it is logical that genes previously investigated in *Neurospora* (hyphal growth) and *Saccharomyces* (yeast growth) are not highly relevant to parasitic-phase growth. These results imply that control of the parasitic spherule growth form relies on a different set of genes than those that are important for hyphal or yeast phase growth in other fungi, as opposed to unique biological functions of the same gene set.

### Comparison with Previous Transcriptional Profiling

In a previous study, Johannesson *et al*. profiled gene expression between two isolates of *C. posadasii* using a microarray with 70mers for 1000 of the 9,910 *Coccidioides* genes [18]. There was relatively little overlap between the results of that study and those reported here (44% of saprobic-phase up-regulated genes in common, 20% of parasitic-phase up-regulated genes). Several critical factors likely account for the disparate results observed between this study and the previous one. There were obvious methodology differences –1,000 gene microarray vs. whole transcriptome sequencing and two isolates of one species instead of one representative isolate for two species. There also were significant biological differences in experimental design between the studies as well. The previous study collected mRNA at two parasitic phase timepoints: late isotropic growth (36 hours post-inoculum) and endospore release (132 hours). Here, we collected mRNA from parasitic-phase spherules undergoing segmentation (96 hours). Given the significant morphological changes during spherule maturation and endospore release, we predict that there are many changes in gene expression within the parasitic cycle that would account for the disparate results of this study and the previous one. Interestingly, both studies observed approximately 50% overlap in differential gene expression between the two isolates/species used, whether they were from the same species (Johannesson et al., 2006) or different species (this study). This amount of overlap indicates that there is considerable variation in gene expression between isolates and species. Although understanding the basis of differences in expression would be interesting, in terms of the prevention and treatment of disease, our chief concern is with identifying the core set of genes responsible for dimorphic growth and virulence during the parasitic phase in both species.

## Materials and Methods

### Isolates and Media

*C. posadasii* isolate C735 and *C. immitis* isolate RS, were grown as the saprobic (hyphae) and parasitic (spherule) phases. Arthroconidia were isolated from mycelia grown on GYE agar plates (1% glucose, 0.5% yeast extract, 1.5% agar) at 30°C for 4 to 6 weeks and used to inoculate cultures. To induce the spherule growth morphology, parasitic phase cultures were grown in modified Converse liquid medium [49] containing 15.96 mM ammonium acetate, 3.7 mM KH_2_PO_4_, 3.0 mM K_2_HPO_4_, 1.6 mM MgSO_4_, 0.0125 mM ZnSO_4_, 0.24 mM NaCl, 0.0204 mM CaCl_2_, 0.143 mM NaHCO_3_, 0.5 g of Tamol SN/liter, 4.0 g of glucose/liter, and 0.05 g of N-Z amine/liter, as previously described [50]. Parasitic phase cultures were purged with 10% CO_2_ immediately after inoculation, and then again 48 hours later. The cultures were incubated at 39°C in a 140-rpm shaking incubator. Parasitic phase spherules in near-synchronized, pre-endosporulation stage of development were harvested at 96 hours post-inoculation. To induce the hyphal morphology, saprobic phase cultures were grown in liquid GYE media and the cultures were incubated at 30°C in a shaking incubator as above. Saprobic phase hyphae were harvested by vacuum filtration 96 hours post-inoculation and frozen in liquid N_2_.

### Isolation of Total RNA

RNA was released from frozen saprobic phase hyphae by grinding with a mortar and pestle. To release RNA from parasitic phase spherules, the samples were mechanically disrupted using a bead mill (Mini-Beadbeater, Biospec Products, Bartlesville, OK). Total RNA was isolated from both the hyphal and spherule phases using a Qiagen RNeasy Plant Mini Kit (Qiagen; Valencia CA, USA). Three biological replicates of hyphal and spherule RNA were prepared.

### RNAseq Library Preparation and Illumina Sequencing

mRNA was isolated from the total RNA using Dynabeads Oligo(dT)_25_ (Invitrogen) on a magnetic separation stand (Promega, Madison WI). The isolated mRNA was then chemically fragmented using a fragmentation buffer (Ambion, Austin TX) and reversed transcribed to cDNA using ArrayScript reverse transcriptase (Ambion, Autstin TX). The cDNA ends were repaired using End-It DNA end-repair (Epicentre, Madison WI). We prepared adapters for Illumina single-end sequencing [51], which were ligated onto the cDNA fragments. The fragments were amplified using a previously-described emulsion PCR protocol [51]. Finally, 200 base-pair fragments were selected from a 2% agarose gel and purified with a Min-Elute gel extraction kit (Qiagen, Valencia CA). Library quality was assessed by Bioanalyzer assay. Thirty-six base-pair single-end reads were sequenced at the Vincent J. Coates Genomics Sequencing Facility at U.C. Berkeley on an Illumina Genome Analyzer II (Illumina, Inc., San Diego CA). Sequences are available at the NCBI short read archive (http://trace.ncbi.nlm.nih.gov/Traces/sra/; accession number SRA054882).

### Genomes

*C. immitis* isolate RS genome sequence and annotation version 3 from the Broad Institute [8] and *C. posadasii* isolate C735 genome sequence version 1 from TIGR/J. Craig Venter Institute [19] were used for all analyses. To ensure that the gene models used were equivalent and accurate homolog predictions were used, a genome alignment of the two species was constructed using Mercator/MAVID [52], [53], [54] and *C. posadasii* gene models were inferred from the *C. immitis* annotation. Gene Ontology (GO) terms [55] were derived from homology with *S. cerevisiae, S. pombe* and *Neurospora* spp. The program RepeatMasker [56] was used to predict repetitive elements. The program SignalP [57] was used to predict signal peptides for putative secreted proteins.

### Data Analysis

Thirty-six base-pair Illumina reads were mapped to the *C. immitis* RS and *C. posadasii* C735 genomes respectively using Tophat/Bowtie [58]. Reads mapped to the genome were assigned to genes using Python scripts (http://python.org). Median-difference boxplots were generated in R (http://www.r-project.org). Statistically significant differences between levels of gene expression in the saprobic and parasitic phases were assessed using DESeq [59]. Significance of functional enrichment of GO terms in differentially expressed gene sets was assessed using the hypergeometric distribution [60]. All resulting *p*-values were adjusted for multiple hypothesis testing using the Benjamini-Hochberg method [61].

## Supporting Information

Table S1 All genes with significantly higher expression (up-regulated) in the saprobic phase in both *C. immitis* and *C. posadasii*. *: Predicted secreted; +: Gene under positive selection [19]; G: Gene gained in *Coccidioides* [19]; H, S: Previously found to be up-regulated in saprobic-phase Hyphae or parasitic-phase Spherules by microarray [18].
(PDF)

Table S2 All genes with significantly higher expression (up-regulated) in the parasitic phase in both *C. immitis* and *C. posadasii*. *: Predicted secreted; +: Gene under positive selection [19]; G: Gene gained in *Coccidioides* [19]; H, S: Previously found to be up-regulated in saprobic-phase Hyphae or parasitic-phase Spherules by microarray [18].
(PDF)

Table S3 Gene ontology (GO) terms significantly enriched (*p*-value <0.05) in saprobic-phase and parasitic-phase up-regulated gene sets.
(DOCX)
