# Supplementary material for: Comparative Transcriptomics of the Saprobic and Parasitic Growth Phases in Coccidioides spp
Source: PLoS One. 2012 Jul 20;7(7):e41034. doi: 10.1371/journal.pone.0041034 (PMC3401177; doi:10.1371/journal.pone.0041034)
Supplement: Table S1 — All genes with significantly higher expression (up-regulated) in the saprobic phase in both C. immitis and C. posadasii. *: Predicted secreted; +: Gene under positive selection [19]; G: Gene gained in Coccidioides [19]; H, S: Previously found to be up-regulated in saprobic-phase Hyphae or parasitic-phase Spherules by microarray [18]. (PDF) [file pone.0041034.s001.pdf]

Table S1: All genes up-regulated in saprobic phase

BROAD ID	*C. immitis* H/S	*C. immitis* *p*-value	*C. posadasii* H/S	*C. posadasii* p-value	Gene description	Previous Studies
CIMG_00035	12.29	8.39E-79	2.27	9.55E-07	3-hydroxyisobutyrate dehydrogenase	
CIMG_00039	3.17	5.12E-13	1.96	0.00035	conserved hypothetical protein	
CIMG_00043	2.62	1.05E-09	1.79	0.00119	dynactin Arp1 p25 subunit	
CIMG_00044	2.30	1.08E-10	2.87	7.97E-10	ADP-ribosylation factor 6	
CIMG_00065	2.56	3.24E-11	1.80	0.00107	Mis12-Mtw1 domain-containing protein	
CIMG_00067	3.02	1.59E-10	1.39	0.03698	conserved hypothetical protein (hosphotransferase enzyme family)	
CIMG_00078	1.82	0.00014	2.74	3.25E-09	conserved hypothetical protein	
CIMG_00080	2.01	2.15E-08	8.23	1.04E-24	TAM domain methyltransferase	
CIMG_00090	1.64	0.00035	1.59	0.00110	LRP16 family protein	
CIMG_00093	2.65	4.96E-14	1.85	0.00052	glycolipid transfer protein HET-C2	
CIMG_00099	40.63	2.37E-103	65.14	7.19E-84	C2H2 zinc finger domain-containing protein	
CIMG_00104	7.23	0.00002	3.03	0.00241	conserved hypothetical protein	*
CIMG_00107	2.03	1.75E-06	1.70	0.00287	conserved hypothetical protein	
CIMG_00108	1.92	1.42E-06	4.41	5.86E-21	sulfate transporter	
CIMG_00110	4.66	5.98E-35	1.88	0.00009	potassium ion channel Yvc1	
CIMG_00113	1.31	0.04877	2.27	2.45E-07	glutaminyl-tRNA synthetase	
CIMG_00121	2.09	3.73E-07	3.02	2.14E-13	MFS alpha-glucoside transporter	
CIMG_00146	2.31	5.42E-10	7.82	7.15E-20	conserved hypothetical protein	
CIMG_00152	2.20	3.89E-06	2.29	3.05E-06	MFS monocarboxylate transporter	
CIMG_00185	2.91	3.72E-17	3.28	1.61E-12	inositol-3-phosphate synthase	
CIMG_00189	7.30	5.26E-27	14.55	8.74E-28	conserved hypothetical protein (LIM domain)	
CIMG_00202	1.56	0.02240	24.89	2.33E-54	conserved hypothetical protein	
CIMG_00216	17.84	3.07E-103	1.74	0.00619	copper amine oxidase	H
CIMG_00223	3.83	3.83E-19	1.89	0.00702	FAD synthetase	
CIMG_00236	1.97	2.77E-07	2.07	0.00022	isopentenyl-diphosphate delta-isomerase	
CIMG_00245	9.92	5.42E-66	5.98	7.55E-15	DENN domain-containing protein	
CIMG_00246	1.90	3.02E-06	2.58	5.95E-06	conserved hypothetical protein	
CIMG_00290	8.23	3.77E-68	4.87	4.93E-21	MFS transporter	
CIMG_00291	1.47	0.00447	1.48	0.00314	Spc97/Spc98 family protein	
CIMG_00297	1.96	7.09E-07	1.86	0.01127	conserved hypothetical protein	
CIMG_00302	7.52	4.29E-59	4.12	1.21E-12	conserved hypothetical protein	*
CIMG_00304	1.59	0.00023	2.01	0.00003	importin beta-5 subunit	
CIMG_00321	1.57	0.00046	1.84	0.00018	Prolyl-tRNA synthetase	*
CIMG_00323	2.31	1.32E-06	2.98	1.09E-08	WD domain-containing protein	
CIMG_00328	5.67	7.98E-16	1.67	0.00654	urease accessory protein UreD	
CIMG_00329	2.06	4.42E-09	1.64	0.00343	nuclear cohesin complex subunit	
CIMG_00338	4.29	7.80E-09	1.54	0.00346	polysaccharide deacetylase	*
CIMG_00345	1.70	0.03204	14.86	1.02E-50	carbohydrate-binding protein	
CIMG_00346	1.37	0.04742	1.65	0.00139	phosphatidylinositol:UDP-GlcNAc transferase PIG-C	
CIMG_00366	4.99	1.73E-25	1.82	0.00085	annexin ANXC4	
CIMG_00367	2.18	1.08E-08	9.50	4.24E-31	Sep4b	
CIMG_00372	1.45	0.00885	2.33	7.59E-07	conserved hypothetical protein (Dopa 4,5-dioxygenase family)	
CIMG_00374	2.42	1.62E-09	4.43	9.59E-18	conserved hypothetical protein	
CIMG_00383	1.37	0.03407	1.92	0.00017	RRM domain-containing protein	
CIMG_00385	1.54	0.00282	1.32	0.03032	WD repeat protein	
CIMG_00389	7.44	4.01E-52	4.70	4.67E-15	conserved hypothetical protein	
CIMG_00390	14.62	1.84E-42	9.52	1.40E-19	conserved hypothetical protein	
CIMG_00400	10.35	8.59E-75	2.34	0.00059	UMTA (methyltransferase)	
CIMG_00422	1.66	0.00009	2.56	1.52E-08	Rac1 GTPase	
CIMG_00438	2.01	5.22E-07	1.66	0.01865	dolichyl-phosphate-mannose-protein mannosyltransferase	
CIMG_00440	2.69	3.98E-16	1.94	0.00025	palmitoyltransferase erf2	
CIMG_00448	1.57	0.00250	16.12	3.93E-41	conserved hypothetical protein	
CIMG_00457	1.99	4.64E-08	1.67	0.00080	phosphoserine phosphatase	
CIMG_00462	2.50	0.00051	2.24	0.00204	conserved hypothetical protein	
CIMG_00474	2.70	4.35E-13	3.10	1.44E-11	aminoalcoholphosphotransferase	
CIMG_00475	1.48	0.00755	2.03	0.00001	nipsnap family protein	
CIMG_00483	2.06	3.17E-07	1.35	0.03727	conserved hypothetical protein (Ankyrin repeat)	
CIMG_00494	1.60	0.00612	2.11	0.00005	conserved hypothetical protein	
CIMG_00513	2.20	4.44E-09	3.07	3.68E-08	hypothetical protein (linker histone H1 and H5 family)	
CIMG_00522	2.70	6.64E-15	9.02	1.75E-36	dityrosine transporter A	
CIMG_00523	1.40	0.00715	9.49	2.45E-30	Dit2 protein	
CIMG_00524	4.70	6.18E-35	5.08	1.11E-19	conserved hypothetical protein (putative pyoverdine/dityrosine biosynthesis protein)	
CIMG_00529	2.29	2.91E-06	14.39	1.80E-53	conserved hypothetical protein	
CIMG_00536	10.48	5.35E-61	9.22	5.20E-28	conserved hypothetical protein	*
CIMG_00548	1.43	0.00515	1.33	0.03840	chromatin remodeling complex subunit	
CIMG_00614	3.87	6.16E-23	1.69	0.02009	methylmalonate-semialdehyde dehydrogenase	

*: Predicted secreted;
+: Gene under positive selection [19]; G: Gene gained in Coccidioides [19]
H, S: Up-regulated in Hyphae or Spherule by microarray [18]

Table S1: All genes up-regulated in saprobic phase

CIMG_00621	2.16	8.01E-09	5.82	3.79E-18	histidyl-tRNA synthetase	
CIMG_00625	3.00	2.53E-18	1.87	0.00010	kex protein	*
CIMG_00653	7.63	1.05E-19	6.56	7.99E-13	hypothetical protein	
CIMG_00654	11.96	6.74E-90	14.24	2.98E-42	conserved hypothetical protein	
CIMG_00661	1.51	0.03058	1.64	0.00135	oxidoreductase	
CIMG_00670	2.09	5.98E-09	1.70	0.00032	MFS multidrug transporter	
CIMG_00689	17.62	3.07E-103	5.72	1.32E-18	Conseved hypothetical protein (DUF221)	
CIMG_00690	1.97	0.00013	1.78	0.00106	conserved hypothetical protein	
CIMG_00693	11.91	1.75E-79	3.70	1.49E-08	conserved hypothetical protein (CFEM domain)	*
CIMG_00694	11.54	1.46E-80	6.30	4.18E-19	siderophore iron transporter	
CIMG_00695	1.59	0.00034	2.55	9.89E-09	SH3 domain-containing protein	
CIMG_00711	2.68	5.09E-15	1.86	0.00011	conserved hypothetical protein	
CIMG_00714	1.98	5.11E-08	2.32	1.67E-06	phosphomevalonate kinase	
CIMG_00716	1.35	0.03109	1.55	0.01708	20S proteasome maturation protein Ump1	
CIMG_00744	1.42	0.00910	1.54	0.03709	conserved hypothetical protein	
CIMG_00765	2.03	1.96E-07	2.37	0.00002	asparaginyl-tRNA synthetase	
CIMG_00773	2.54	1.90E-12	11.58	4.85E-43	conserved hypothetical protein	
CIMG_00782	3.15	2.12E-19	7.01	1.60E-21	siderophore iron transporter mirC	
CIMG_00786	1.71	0.00003	1.75	0.00493	cell division control protein 42	
CIMG_00805	24.02	1.01E-125	10.97	5.12E-29	conserved hypothetical protein (putative pre-mRNA-splicing factor cwc15)	
CIMG_00823	4.52	1.40E-22	2.23	1.65E-06	conserved hypothetical protein	
CIMG_00829	2.17	2.89E-10	3.10	2.07E-12	RING finger membrane protein	
CIMG_00844	5.80	8.36E-13	3.51	8.71E-07	conserved hypothetical protein	
CIMG_00847	2.90	4.92E-18	3.00	2.80E-08	GTP binding protein	
CIMG_00855	1.55	0.00196	1.47	0.00770	prenyl cysteine carboxyl methyltransferase Ste14	
CIMG_00856	12.36	2.36E-27	7.69	3.62E-18	conserved hypothetical protein	
CIMG_00860	1.75	0.00001	2.18	9.62E-07	diacylglycerol O-acyltransferase	
CIMG_00881	3.27	3.92E-23	20.34	1.72E-39	flotillin domain-containing protein	
CIMG_00904	4.40	1.17E-34	5.81	3.62E-19	cell pattern formation-associated protein	
CIMG_00911	1.71	0.00003	1.88	0.00014	60S ribosomal protein L19	
CIMG_00917	4.36	3.92E-28	7.71	7.78E-30	conserved hypothetical protein (fungal Zn(2)-Cys(6) binuclear cluster domain)	
CIMG_00925	203.36	1.25E-150	165.69	5.12E-72	conserved hypothetical protein	*
CIMG_00926	1.81	0.00017	6.12	3.30E-14	MFS transporter	
CIMG_00927	11.00	2.65E-57	9.89	1.34E-22	acyl-CoA synthetase	
CIMG_00936	2.35	3.54E-07	6.82	8.49E-28	nuclear migration protein	
CIMG_00940	4.59	2.03E-32	5.08	6.86E-19	protein kinase	
CIMG_00943	4.70	2.09E-30	2.15	0.01024	L-ornithine 5-monooxygenase	
CIMG_00947	2.15	1.71E-07	2.44	1.14E-06	conserved hypothetical protein	
CIMG_00948	1.69	0.00004	2.09	2.38E-06	prolidase	
CIMG_00949	1.56	0.00528	1.99	0.00047	conserved hypothetical protein (BTB/POZ domain)	
CIMG_00951	1.38	0.01873	1.80	0.00056	TOR signaling pathway protein TipA	
CIMG_00970	2.93	6.13E-06	3.60	2.60E-06	conserved hypothetical protein	*
CIMG_00972	1.83	6.31E-06	1.70	0.00266	cytoskeletal actin	
CIMG_00975	1.59	0.00051	1.97	0.00067	DUF907 domain-containing protein	*
CIMG_00981	2.15	4.41E-09	1.61	0.00331	NAD-specific glutamate dehydrogenase	
CIMG_00993	1.76	0.00002	2.05	0.00009	pre-mRNA branch site protein p14	
CIMG_00995	2.33	1.29E-10	2.08	5.38E-06	conserved hypothetical protein (putative vacuolar membrane protein)	
CIMG_00999	1.81	0.00002	1.71	0.00197	SIR2 family histone deacetylase	
CIMG_01004	2.67	1.31E-15	1.59	0.01575	double-strand-break repair protein rad21	
CIMG_01010	1.40	0.01636	3.41	1.93E-12	conserved hypothetical protein (fungal Zn(2)-Cys(6) binuclear cluster domain)	
CIMG_01012	2.59	1.28E-11	1.92	0.00028	riboflavin kinase	
CIMG_01028	4.02	1.31E-25	2.86	6.81E-12	conserved hypothetical protein	
CIMG_01040	1.69	0.00019	1.22	0.00786	conserved hypothetical protein (DUF258)	
CIMG_01041	2.25	0.00066	1.85	0.01139	conserved hypothetical protein (C2H2 type zinc finger)	
CIMG_01045	1.63	0.00064	1.66	0.00857	F-box protein	
CIMG_01059	1.79	6.63E-06	1.93	9.90E-06	RecQ family helicase MusN	
CIMG_01064	1.96	3.83E-07	2.02	1.34E-06	KH domain RNA binding protein	
CIMG_01096	2.03	7.00E-09	1.56	0.00369	aspartate aminotransferase	
CIMG_01102	10.42	1.87E-75	6.29	7.82E-26	cell wall glucanase	*
CIMG_01124	1.51	0.00123	1.77	0.00557	ADP-ribosylation factor	
CIMG_01129	5.10	5.50E-36	2.26	4.96E-07	MFS monocarboxylate transporter	
CIMG_01132	3.07	7.38E-16	1.49	0.01526	Conserved hypothetical protein	
CIMG_01149	1.52	0.00229	1.86	0.00039	DNA lyase	
CIMG_01157	1.90	0.00007	1.81	0.00182	conserved hypothetical protein (short chain dehydrogenase)	
CIMG_01159	1.86	1.07E-06	1.59	0.00087	general amidase GmdA	
CIMG_01161	2.23	6.84E-10	2.20	4.91E-06	cell division control protein 2	
CIMG_01166	1.42	0.00824	3.76	9.00E-18	3-methyl-2-oxobutanoate dehydrogenase	
CIMG_01172	1.31	0.04891	1.46	0.01723	cytomegalovirus gH-receptor family protein	
CIMG_01181	2.45	2.29E-12	69.98	9.02E-51	conserved hypothetical protein (cerato-platanin family)	*
CIMG_01182	16.38	9.65E-24	160.61	8.62E-40	conserved hypothetical protein (DUF2349)	*

*: Predicted secreted;
+: Gene under positive selection [19]; G: Gene gained in Coccididioides [19]
H, S: Up-regulated in Hyphae or Spherule by microarray [18]

Table S1: All genes up-regulated in saprobic phase

CIMG_01183	3.45	6.49E-22	9.34	2.49E-24	conserved hypothetical protein	
CIMG_01185	12.02	3.94E-58	8.30	1.03E-27	hypothetical protein	
CIMG_01186	2.04	0.00002	1.71	0.01420	conserved hypothetical protein	
CIMG_01197	1.93	4.34E-06	1.90	0.00167	conserved hypothetical protein (Cytochrome P450)	*
CIMG_01205	2.94	2.15E-17	4.44	5.34E-20	beta-glucosidase 6	
CIMG_01236	1.77	5.07E-06	3.82	1.08E-15	serine/threonine kinase PakA	
CIMG_01242	2.60	2.96E-11	1.76	0.00050	Fe-containing alcohol dehydrogenase	
CIMG_01246	1.99	1.07E-07	3.92	1.57E-10	PH domain-containing protein	
CIMG_01248	1.37	0.01151	1.70	0.00046	catabolite repression protein creC	
CIMG_01252	1.55	0.00169	2.13	3.89E-06	telomere silencing protein Zds1	
CIMG_01261	1.83	2.49E-06	1.54	0.00369	conserved hypothetical protein	
CIMG_01266	8.00	4.58E-59	7.43	6.12E-25	alanine aminotransferase	
CIMG_01269	3.87	1.26E-28	1.93	0.00034	peroxisomal biogenesis factor PEX11	
CIMG_01271	1.48	0.00339	1.53	0.03819	conserved hypothetical protein (fungal Zn(2)-Cys(6) binuclear cluster domain)	
CIMG_01279	3.58	4.50E-24	1.89	0.00088	conserved hypothetical protein	
CIMG_01303	7.02	4.65E-33	9.13	7.63E-23	BYS1 domain-containing protein	* H
CIMG_01304	1.64	0.00027	1.94	0.00070	GDP-mannose pyrophosphorylase A	
CIMG_01308	13.23	1.09E-18	2.29	0.00002	conserved hypothetical protein	
CIMG_01313	2.88	4.88E-13	4.57	1.80E-17	fumarylacetoacetase	
CIMG_01314	2.72	3.10E-10	2.89	3.24E-08	maleylacetoacetate isomerase	
CIMG_01317	3.44	3.70E-20	3.52	9.41E-13	adenosylmethionine-8-amino-7-oxononanoate transaminase	
CIMG_01328	3.23	7.22E-12	6.08	6.11E-28	monooxygenase	
CIMG_01340	2.89	3.44E-13	3.50	5.00E-14	conserved hypothetical protein (PHD finge,; transcription factor S-II domain, SPOC domain)	
CIMG_01344	1.53	0.00101	1.45	0.00278	ubiquitin-protein ligase Ufd4	
CIMG_01346	1.35	0.02475	2.01	0.00007	nicotinamide mononucleotide adenylyl transferase	
CIMG_01348	1.63	0.00008	2.05	0.00103	phosphoserine phosphatase	
CIMG_01353	2.43	8.37E-11	3.47	2.71E-14	sugar isomerase	
CIMG_01357	1.70	0.00017	1.75	0.00001	2-oxoisovalerate dehydrogenase alpha subunit	
CIMG_01360	1.94	5.21E-06	2.01	0.00002	cellular morphogenesis protein	
CIMG_01361	3.40	2.69E-23	2.94	1.54E-08	5'-methylthioadenosine phosphorylase	
CIMG_01363	7.15	2.20E-39	4.60	1.37E-18	beta-glucosidase	* H
CIMG_01375	1.50	0.00331	1.95	0.00077	eukaryotic peptide chain release factor GTP-binding subunit	
CIMG_01376	1.94	3.94E-06	1.98	0.00008	pre-rRNA processing protein	
CIMG_01383	2.35	6.59E-11	3.06	2.58E-09	Ser/Thr protein phosphatase	
CIMG_01413	3.26	6.28E-22	2.47	2.29E-08	ribulose-phosphate 3-epimerase	
CIMG_01418	3.77	2.95E-25	4.92	6.10E-12	ABC transporter	
CIMG_01422	5.26	3.35E-34	4.62	7.10E-12	tubulin alpha-2 chain	
CIMG_01426	2.35	1.90E-12	1.40	0.04234	gamma-butyrobetaine hydroxylase subfamily	
CIMG_01428	2.19	8.63E-06	5.32	3.99E-15	small oligopeptide transporter	
CIMG_01429	14.94	2.21E-83	13.83	8.38E-30	nonribosomal peptide synthetase 6	
CIMG_01430	13.09	1.54E-86	23.07	1.87E-49	ABC multidrug transporter	
CIMG_01431	11.43	2.29E-78	21.76	2.73E-46	conserved hypothetical protein (putative pyridine nucleotide-disulphide oxidoreductase)	
CIMG_01432	3.23	2.22E-14	3.69	5.01E-07	L-ornithine 5-monooxygenase	
CIMG_01434	12.93	4.38E-82	5.54	1.66E-21	long-chain-fatty-acid-CoA ligase	
CIMG_01435	21.50	2.09E-104	14.17	1.28E-35	acetylase	
CIMG_01436	9.65	3.58E-69	11.59	2.39E-26	siderophore iron transporter	
CIMG_01441	1.98	4.44E-08	1.44	0.01270	protein kinase	
CIMG_01460	1.63	0.00024	1.88	0.00005	transcription regulator BDF1	
CIMG_01490	1.95	2.34E-07	1.52	0.04008	conserved hypothetical protein	
CIMG_01507	1.88	0.00006	2.83	1.06E-08	conserved hypothetical protein	
CIMG_01510	14.59	3.39E-98	4.02	8.10E-13	acetyl-coenzyme A synthetase FacA	
CIMG_01511	1.54	0.00193	2.15	0.00003	nucleoporin SONB	
CIMG_01518	1.68	0.00317	3.59	3.98E-08	conserved hypothetical protein	
CIMG_01539	4.14	1.98E-31	2.79	5.76E-09	conserved hypothetical protein	
CIMG_01541	1.90	7.69E-06	2.75	6.52E-09	GPI mannosyltransferase 2	*
CIMG_01542	1.75	7.70E-06	2.47	1.40E-09	proteasome activator subunit 4	
CIMG_01543	3.09	6.16E-16	2.93	3.20E-06	MOSC domain-containing protein	
CIMG_01545	2.02	2.01E-08	1.97	0.00002	2-deoxy-D-gluconate 3-dehydrogenase	
CIMG_01546	2.97	7.31E-19	2.93	1.43E-08	fructose-1,6-bisphosphatase	
CIMG_01550	1.37	0.04053	2.40	0.00120	conserved hypothetical protein	
CIMG_01555	2.44	9.69E-12	2.99	3.28E-08	saccharopine dehydrogenase	
CIMG_01558	2.07	1.62E-08	1.55	0.03165	glycerol-3-phosphate O-acyltransferase	
CIMG_01570	3.60	1.41E-20	5.21	6.14E-18	sucrase/ferredoxin domain-containing protein	
CIMG_01571	3.69	1.26E-13	5.72	3.69E-20	C2H2 zinc finger domain-containing protein	
CIMG_01576	2.07	2.29E-08	4.73	3.29E-14	mannose-1-phosphate guanylyltransferase	
CIMG_01582	1.43	0.01362	1.94	4.60E-06	glycerol kinase	
CIMG_01584	2.93	4.42E-20	4.14	4.24E-09	conserved hypothetical protein	
CIMG_01588	2.10	0.00009	2.60	0.00001	conserved hypothetical protein (microtubule associated)	

*: Predicted secreted;
+: Gene under positive selection [19]; G: Gene gained in Coccidioides [19]
H, S: Up-regulated in Hyphae or Spherule by microarray [18]

Table S1: All genes up-regulated in saprobic phase

CIMG_01597	1.55	0.00584	2.70	0.00002	2-oxoglutarate dehydrogenase E1 component	
CIMG_01600	2.24	6.47E-08	1.27	0.02747	conserved hypothetical protein (BSD domain, TFIIH p62 subunit N-terminal domain)	
CIMG_01606	3.14	5.86E-14	1.32	0.01738	tubulin-specific chaperone	
CIMG_01620	1.65	0.00066	3.84	3.58E-18	conserved hypothetical protein (RING finger)	
CIMG_01621	2.95	7.48E-15	2.63	2.80E-08	conserved hypothetical protein	
CIMG_01622	1.62	0.00025	1.56	0.00186	N-terminal acetyltransferase catalytic subunit	
CIMG_01626	1.69	0.00193	2.03	0.00041	conserved hypothetical protein	
CIMG_01631	1.52	0.00685	2.85	6.27E-08	phosphatidylinositol phospholipase C	
CIMG_01641	1.63	0.00012	1.61	0.00502	histone deacetylase SIR2	
CIMG_01646	3.14	1.16E-20	2.13	0.00006	G2/M-specific cyclin NimE	
CIMG_01661	1.93	1.03E-07	2.52	2.52E-09	Conserved hypothetical protein (putative autophagy regulatory protein Atg2)	
CIMG_01663	10.60	6.95E-74	2.59	1.00E-06	acyl-coenzyme A synthetase ACSM5	
CIMG_01667	2.35	2.13E-10	2.01	0.00007	ubiquitin conjugating enzyme	
CIMG_01670	2.55	1.97E-14	1.58	0.02315	3,4-dihydroxy-2-butanone 4-phosphate synthase	
CIMG_01674	2.62	7.89E-09	4.00	1.68E-13	magnesium dependent phosphatase	**G**
CIMG_01686	3.73	1.01E-22	2.53	1.39E-06	spindle pole body component (alp14)	
CIMG_01687	1.40	0.02638	2.20	2.49E-06	nuclear migration protein NudF	
CIMG_01697	1.69	0.00005	2.28	2.33E-06	leucyl-tRNA synthetase	
CIMG_01708	1.69	0.00006	2.32	7.88E-07	GPI transamidase component GAA1	
CIMG_01710	1.68	0.03629	1.53	0.00149	RTA1 domain-containing protein	
CIMG_01713	10.19	2.88E-55	4.30	1.99E-14	kinesin family protein	
CIMG_01715	1.38	0.00879	2.62	5.25E-09	mitogen-activated protein kinase	
CIMG_01735	2.09	1.32E-08	1.73	0.01285	mitochondrial RNA splicing protein	
CIMG_01760	1.51	0.00217	2.21	2.97E-07	autophagy protein Apg9	
CIMG_01786	1.44	0.01522	2.03	0.00003	metal homeostatis protein bsd2	
CIMG_01789	2.43	7.33E-11	1.93	0.00244	conserved hypothetical protein (transmembrane alpha-helix domain)	*
CIMG_01793	1.52	0.00113	1.83	0.00014	threonyl-tRNA synthetase	
CIMG_01802	4.70	8.20E-07	3.06	0.00003	conserved hypothetical protein	*
CIMG_01807	2.05	1.61E-08	1.99	0.00020	aspartyl-tRNA synthetase	
CIMG_01816	1.79	8.21E-06	1.85	0.00139	bZIP transcription factor	
CIMG_01819	3.68	2.48E-20	1.67	0.01367	conserved hypothetical protein	
CIMG_01834	4.23	0.00164	2.86	0.00007	conserved hypothetical protein	
CIMG_01839	1.84	3.07E-06	2.88	2.68E-09	Leucine-Rich Repeat domain-containing protein	
CIMG_01844	2.07	8.79E-09	1.50	0.00175	37S ribosomal protein Rsm22	
CIMG_01854	4.58	1.90E-35	3.80	1.35E-11	conserved hypothetical protein	
CIMG_01860	3.56	2.48E-20	6.17	1.68E-22	conserved hypothetical protein (putative beta-lactamase)	*
CIMG_01861	3.57	2.76E-13	3.51	5.39E-15	conserved hypothetical protein (putative HC-toxin synthetase)	
CIMG_01871	1.55	0.00970	5.78	3.40E-24	prenylcysteine lyase	*
CIMG_01890	2.80	6.11E-13	1.75	0.00094	spindle-pole body protein	
CIMG_01894	2.37	6.76E-11	1.80	0.00040	DNA polymerase epsilon catalytic subunit A	
CIMG_01906	1.77	0.00012	2.64	1.33E-08	conserved hypothetical protein	*
CIMG_01913	1.65	0.00092	2.58	2.80E-07	OefC	
CIMG_01923	7.87	8.61E-26	7.09	1.37E-18	conserved hypothetical protein	
CIMG_01926	1.81	0.00001	1.39	0.04727	C2H2 transcription factor	
CIMG_01939	1.63	0.00040	1.49	0.02124	N-acetylglucosaminyl transferase component Gpi1	*
CIMG_01949	2.29	5.57E-10	3.31	6.93E-09	SEC14 cytosolic factor	
CIMG_01950	1.51	0.00827	1.77	0.00417	conserved hypothetical protein (kinetochore protein Mis14 like)	
CIMG_01963	4.03	1.00E-18	2.32	0.00042	conserved hypothetical protein	
CIMG_01967	2.57	8.92E-14	2.03	0.00766	transcriptional regulator	
CIMG_01972	1.73	0.00008	4.04	9.47E-14	protein-tyrosine phosphatase	
CIMG_01987	1.83	0.00048	2.39	5.83E-06	conserved hypothetical protein (DUF2467, C2H2 type zinc finger)	
CIMG_02033	1.76	8.81E-06	18.70	3.51E-39	conserved hypothetical protein	
CIMG_02053	1.61	0.00020	2.98	7.28E-10	conserved hypothetical protein (casein kinase substrate phosphoprotein PP28 domain)	
CIMG_02057	1.58	0.00025	2.26	1.07E-07	anucleate primary sterigmata protein B	
CIMG_02074	1.78	6.19E-06	1.30	0.03187	phospholipase D1	
CIMG_02090	1.61	0.00031	1.70	0.00187	DnaJ domain-containing protein	*
CIMG_02102	1.82	5.51E-06	1.79	0.00052	ATP-dependent RNA helicase DHH1	
CIMG_02103	1.46	0.00360	1.84	0.00005	DNA repair protein Rad18	
CIMG_02109	2.20	9.54E-10	2.42	2.36E-08	nuclear envelope pore membrane protein	
CIMG_02132	1.80	0.00128	2.68	0.00009	histone H2A.2	
CIMG_02133	3.24	1.25E-13	3.34	3.32E-07	histone H2B	
CIMG_02136	1.39	0.02561	1.40	0.01801	F-box domain-containing protein	
CIMG_02161	2.13	1.98E-08	2.57	3.66E-07	valyl-tRNA synthetase	
CIMG_02171	2.71	8.16E-13	4.11	8.61E-14	C2H2 finger domain-containing protein	
CIMG_02172	3.00	1.57E-19	1.66	0.00134	peroxisomal membrane protein	
CIMG_02182	1.85	5.12E-06	1.95	0.00023	conserved hypothetical protein (putative Noc1p protein)	
CIMG_02194	1.65	0.00368	5.14	2.71E-18	conserved hypothetical protein	
CIMG_02196	1.66	0.00014	1.46	0.02711	DnaJ domain-containing protein	

*: Predicted secreted;
+: Gene under positive selection [19]; G: Gene gained in Coccidioides [19]
H, S: Up-regulated in Hyphae or Spherule by microarray [18]

Table S1: All genes up-regulated in saprobic phase

CIMG_02201	1.75	0.00004	1.51	0.02694	mannosyltransferase PMTI	
CIMG_02206	2.13	5.26E-09	1.64	0.00407	oligosaccharyl transferase, STT3 subunit	*
CIMG_02211	4.18	6.36E-31	2.49	7.18E-07	hypothetical protein (putative GPI-anchored glycine-serine rich protein)	*
CIMG_02217	1.90	2.67E-07	14.16	2.92E-37	RNA polymerase Rpb1 C-terminal repeat domain-containing protein	
CIMG_02232	2.51	7.03E-13	2.92	6.83E-08	triacylglycerol lipase	
CIMG_02238	9.10	5.62E-30	4.46	2.13E-16	conserved hypothetical protein (patatin-like phospholipase)	H
CIMG_02245	1.93	2.05E-07	4.56	1.19E-19	conserved hypothetical protein (fungal specific transcription factor domain)	
CIMG_02250	1.94	1.64E-07	1.98	0.00010	conserved hypothetical protein (putative preprotein translocase subunit Sec66)	*
CIMG_02259	7.13	9.49E-56	10.78	1.25E-38	conserved hypothetical protein (DUF2403, DUF2401)	*
CIMG_02262	1.71	0.00006	1.74	0.01634	acetyl-CoA acetyltransferase	
CIMG_02271	1.79	0.00088	1.68	0.02905	cyclin-dependent kinase regulatory subunit	
CIMG_02275	1.56	0.00081	4.14	1.71E-15	hypothetical protein (C2HC4 type zinc finger/RING finger)	
CIMG_02277	16.46	2.76E-80	9.16	2.49E-34	benzoate 4-monooxygenase cytochrome P450	*
CIMG_02279	15.35	1.48E-99	1.88	0.00419	CAS1	*
CIMG_02294	1.32	0.03814	2.27	0.00004	UPD-GlcNAc transporter	
CIMG_02296	5.42	8.18E-26	1.71	0.00197	ELMO/CED-12 family protein	
CIMG_02307	1.44	0.00679	2.37	0.00003	translation machinery-associated protein 22	
CIMG_02314	2.57	2.82E-10	4.22	4.28E-13	polysaccharide deacetylase	
CIMG_02315	23.90	3.50E-103	6.46	2.22E-26	conserved hypothetical protein	
CIMG_02322	2.98	1.74E-14	2.03	1.32E-06	repressor protein	
CIMG_02325	2.85	5.40E-16	2.02	3.52E-06	exocyst complex component Sec10	
CIMG_02329	2.60	1.32E-14	1.76	0.00058	ran GTPase-activating protein 1	
CIMG_02359	2.15	8.07E-10	2.83	7.85E-10	rho-GTPase-activating protein	
CIMG_02360	1.97	0.00001	1.54	0.03513	conserved hypothetical protein (DUF2455)	
CIMG_02374	190.32	6.31E-191	21.48	9.87E-46	acetamidase	
CIMG_02390	34.59	1.84E-123	26.88	2.18E-63	conserved hypothetical protein (helix-loop-helix DNA-binding domain)	
CIMG_02397	1.63	0.03282	5.16	6.12E-12	conserved hypothetical protein (putative pyridine nucleotide-disulphide oxidoreductase)	
CIMG_02399	4.83	0.00043	5.68	9.18E-08	conserved hypothetical protein	*
CIMG_02401	1.80	0.00006	2.17	0.00036	RfeF protein	+
CIMG_02413	2.40	3.22E-11	1.80	0.01030	calmodulin A	
CIMG_02422	2.11	8.31E-09	1.39	0.01501	alpha-1,2-mannosyltransferase	*
CIMG_02424	1.77	0.00001	1.62	0.01174	ATP-dependent permease MDL1	
CIMG_02434	15.13	1.16E-77	7.66	3.17E-32	conserved hypothetical protein (putative inositol-pentakisphosphate 2-kinase)	
CIMG_02454	1.74	0.00056	2.11	4.44E-06	conserved hypothetical protein (C2H2 type zinc finger)	
CIMG_02455	5.24	8.12E-42	16.18	8.71E-56	conserved hypothetical protein (glutathione S-transferase N-terminal domain)	
CIMG_02463	1.65	0.00010	2.23	3.05E-06	conserved hypothetical protein (Chs3p superfamily, putative chitin synthase export chaperone)	
CIMG_02466	2.66	3.34E-14	2.91	3.06E-09	ubiquitin C-terminal hydrolase	
CIMG_02468	6.24	1.04E-48	4.42	3.42E-16	Delta(24(24(1)))-sterol reductase	
CIMG_02479	2.45	1.59E-12	2.24	3.96E-06	conserved hypothetical protein	
CIMG_02492	1.65	0.01429	9.71	3.75E-26	proline-rich antigen 3	*
CIMG_02505	2.63	0.00002	2.04	0.00003	conserved hypothetical protein	
CIMG_02517	2.57	4.22E-14	2.59	2.58E-07	STR2 (ergosterol biosynthesis ERG4/ERG24 family)	
CIMG_02519	3.95	1.19E-17	7.94	7.94E-31	mRNA cleavage factor complex component Pcf11	
CIMG_02520	1.47	0.00126	1.51	0.01141	dimeric dihydrodiol dehydrogenase	
CIMG_02535	3.39	1.52E-20	1.70	0.00373	ribonuclease HI large subunit	
CIMG_02536	1.49	0.00129	1.53	0.00579	peroxisomal membrane protein pex16	
CIMG_02538	3.21	1.07E-12	1.81	0.00735	conserved hypothetical protein (putative DNA repair protein SAE2/CtIP)	
CIMG_02541	1.58	0.00237	1.85	0.00765	zinc knuckle domain-containing protein	
CIMG_02547	3.21	1.48E-22	12.87	5.62E-43	conserved hypothetical protein	
CIMG_02549	3.24	1.89E-21	3.02	3.35E-11	putative GTP-binding protein ypt4	
CIMG_02551	3.28	2.86E-22	3.67	4.04E-13	MFS monosaccharide transporter	
CIMG_02575	1.44	0.00650	2.01	0.00011	conserved hypothetical protein	
CIMG_02603	1.85	0.00011	8.48	1.46E-32	C2H2 transcription factor	
CIMG_02610	2.21	3.75E-10	3.83	1.12E-11	C-5 sterol desaturase	
CIMG_02636	1.65	0.00050	1.58	0.02525	amino acid transporter	
CIMG_02657	2.36	2.10E-11	2.40	0.00002	conserved hypothetical protein	
CIMG_02665	2.96	0.00083	2.40	0.00004	conserved hypothetical protein	
CIMG_02699	3.20	2.53E-18	2.92	4.26E-08	elongation factor 1-gamma	
CIMG_02712	1.71	0.00005	1.51	0.00471	hypercellular protein HypA	
CIMG_02713	2.15	5.93E-08	1.23	0.03341	HATPase_c domain-containing protein	
CIMG_02714	2.45	6.53E-07	1.87	0.00469	conserved hypothetical protein	
CIMG_02795	48.27	2.99E-159	9.15	8.34E-45	endochitinase 1	*
CIMG_02812	1.69	0.01185	15.13	1.13E-52	conserved hypothetical protein (Cytochrome P450)	*
CIMG_02813	1.62	0.00014	5.09	6.34E-20	conserved hypothetical protein	
CIMG_02824	3.44	7.39E-09	4.99	3.20E-13	conserved hypothetical protein	
CIMG_02825	1.50	0.00508	1.83	0.00061	tetratricopeptide repeat domain-containing protein	
CIMG_02827	1.45	0.00599	1.75	0.00069	conserved hypothetical protein	

*: Predicted secreted;
+: Gene under positive selection [19]; G: Gene gained in Coccidioides [19]
H, S: Up-regulated in Hyphae or Spherule by microarray [18]

Table S1: All genes up-regulated in saprobic phase

CIMG_02829	2.77	6.89E-17	2.78	1.03E-08	inner centromere protein	
CIMG_02836	3.92	3.76E-29	5.82	2.18E-24	DNA topoisomerase II	
CIMG_02838	2.48	2.49E-13	3.34	2.46E-12	GTPase activating protein	
CIMG_02860	71.67	3.00E-190	10.36	7.93E-27	chitinase 3	* H
CIMG_02862	1.40	0.02100	1.60	0.00628	conserved hypothetical protein	
CIMG_02880	4.73	8.57E-36	2.36	0.00002	mitochondrial carnitine:acyl carnitine carrier	
CIMG_02896	2.71	9.22E-15	1.58	0.00318	conserved hypothetical protein	
CIMG_02987	3.69	3.69E-27	1.58	0.00605	long chain fatty alcohol oxidase	
CIMG_03014	4.81	7.14E-36	5.09	1.36E-18	iterative type I polyketide synthase	
CIMG_03016	1.50	0.01028	2.45	0.00003	conserved hypothetical protein	
CIMG_03031	2.46	0.00012	3.99	0.00018	conserved hypothetical protein	G
CIMG_03040	3.44	2.34E-22	6.98	3.89E-28	conserved hypothetical protein (MIF domain-containing protein)	
CIMG_03042	2.68	2.92E-14	1.85	0.00050	conserved hypothetical protein (fungal Zn(2)-Cys(6) binuclear cluster domain)	
CIMG_03069	2.45	4.68E-13	2.53	3.94E-09	putative membrane protein (DUF803)	
CIMG_03081	4.21	2.53E-29	3.06	4.35E-10	conserved hypothetical protein (fungal Zn(2)-Cys(6) binuclear cluster domain)	
CIMG_03086	3.17	1.57E-20	1.62	0.00140	dual-specificity MAP kinase kinase dSOR1	
CIMG_03094	1.65	0.00012	1.36	0.02030	conserved hypothetical protein	
CIMG_03099	1.35	0.03062	2.83	7.60E-06	inositolphosphorylceramide-B C-26 hydroxylase	
CIMG_03100	3.89	7.18E-28	2.62	7.55E-12	RNA splicing factor	
CIMG_03105	3.70	5.60E-11	2.33	0.00005	TAM domain methyltransferase	
CIMG_03112	5.36	8.15E-21	1.89	0.00150	phosphoglycerate mutase	
CIMG_03136	1.40	0.04686	6.15	4.84E-18	MFS transporter	
CIMG_03152	3.61	6.91E-14	6.59	2.38E-16	class II aldolase/adducin domain-containing protein	
CIMG_03167	2.36	0.00001	3.74	6.62E-13	conserved hypothetical protein (putative FMN-dependant dehydrogenase)	
CIMG_03169	4.88	2.11E-23	2.67	7.52E-10	conserved hypothetical protein	
CIMG_03173	7.96	9.21E-64	4.09	3.84E-20	conserved hypothetical protein	
CIMG_03175	14.85	1.03E-88	8.58	2.72E-27	glucanase	*
CIMG_03181	1.87	0.00292	21.49	1.66E-61	conserved hypothetical protein	*
CIMG_03182	1.54	0.00095	3.49	6.95E-12	Conserved hypothetical protein (DUF504, endonuclease/exonuclease/phosphatase family)	
CIMG_03210	1.62	0.00095	1.58	0.00041	Poly	
CIMG_03219	1.80	0.00004	4.33	5.54E-15	fungal specific transcription factor domain-containing protein	
CIMG_03226	1.71	0.00007	2.46	0.00017	EBP domain-containing protein	
CIMG_03229	2.12	9.90E-09	3.96	2.71E-14	conserved hypothetical protein	
CIMG_03244	1.53	0.00173	2.03	0.00007	DNA-directed RNA polymerase II largest subunit	
CIMG_03252	6.89	1.20E-56	2.61	9.83E-06	aldehyde dehydrogenase	
CIMG_03253	1.50	0.00203	1.83	0.00035	ubiquitin-protein ligase Sel1/Ubx2	*
CIMG_03259	1.41	0.00984	2.05	0.00033	tRNA-dihydrouridine synthase 3	
CIMG_03286	1.83	0.00822	2.05	0.00003	conserved hypothetical protein (Ankyrin repeat, protein kinase domain)	
CIMG_03287	4.40	1.67E-10	1.70	0.02640	conserved hypothetical protein	
CIMG_03288	1.59	0.00066	1.47	0.04058	translocation complex componenet	
CIMG_03303	1.82	1.35E-06	1.57	0.00370	AMP binding domain-containing protein	
CIMG_03320	6.41	6.92E-35	3.59	2.60E-11	DNA replication initiation factor Cdc45	*
CIMG_03322	3.24	1.35E-12	6.02	3.78E-23	conserved hypothetical protein (F-box domain)	
CIMG_03323	2.62	3.89E-15	3.37	3.55E-12	conserved hypothetical protein (DUF1014)	
CIMG_03328	7.65	2.22E-63	4.81	9.58E-24	homeobox and C2H2 transcription factor	
CIMG_03339	3.04	7.40E-16	2.44	0.00003	ADP-ribosylglycohydrolase	
CIMG_03341	2.70	1.35E-13	1.62	0.00819	class II aldolase/adducin domain-containing protein	
CIMG_03345	4.65	3.42E-09	7.44	1.14E-11	conserved hypothetical protein	
CIMG_03360	2.81	2.48E-15	1.76	0.00044	conserved hypothetical protein (inhibitor of apoptosis domain)	
CIMG_03364	1.67	0.00061	1.58	0.00363	diphthine synthase	
CIMG_03374	1.86	4.32E-06	4.52	4.73E-21	conserved hypothetical protein (SUN-family beta-glucosidase)	*
CIMG_03375	1.53	0.00106	1.91	0.00007	leucine carboxyl methyltransferase superfamily	
CIMG_03387	5.82	9.56E-32	7.62	4.37E-18	histone H4	
CIMG_03388	3.33	1.27E-12	3.88	2.70E-08	histone H3	
CIMG_03389	2.41	4.12E-08	1.78	0.00284	integral membrane protein (mvp17 /PMP22 family)	
CIMG_03392	1.68	0.00013	1.67	0.00896	5'-3' exoribonculease Dhp1	
CIMG_03393	2.81	2.03E-16	2.12	3.33E-07	intracellular protein transporter UsoA	
CIMG_03394	2.01	1.84E-08	2.58	1.66E-08	isoleucyl-tRNA synthetase, cytoplasmic	
CIMG_03403	1.88	1.42E-06	9.42	5.21E-38	conserved hypothetical protein (peptidase family M20/M25/M40)	
CIMG_03406	1.74	0.00004	1.93	0.00121	argininosuccinate synthetase	
CIMG_03408	7.12	3.26E-57	2.71	4.07E-11	low temperature essential protein 1	
CIMG_03411	1.57	0.00246	3.35	3.86E-11	conserved hypothetical protein	
CIMG_03419	1.46	0.00438	1.64	0.00553	vesicle-fusing ATPase	
CIMG_03431	2.00	0.00020	1.62	0.03370	conserved hypothetical protein	
CIMG_03445	1.55	0.00183	1.66	0.01334	hypothetical protein (triose-phosphate transporter family)	
CIMG_03448	1.47	0.02665	1.60	0.00123	transcription factor tfiiib complex subunit brf1	
CIMG_03450	2.47	7.02E-07	3.99	6.59E-13	copper resistance-associated P-type ATPase	
CIMG_03451	2.96	2.29E-13	1.66	0.00540	crossover junction endonuclease mus81	
CIMG_03459	1.45	0.01786	2.79	4.08E-07	Zn-dependent hydrolase/oxidoreductase	

*: Predicted secreted;
+: Gene under positive selection [19]; G: Gene gained in Coccidioides [19]
H, S: Up-regulated in Hyphae or Spherule by microarray [18]

Table S1: All genes up-regulated in saprobic phase

CIMG_03464	1.82	3.77E-06	1.90	0.00043	signal peptidase I	
CIMG_03466	2.61	5.12E-12	1.46	0.04719	conserved hypothetical protein (BTB/POZ domain)	
CIMG_03472	4.44	2.22E-27	1.56	0.03607	extracellular serine-threonine rich protein	*
CIMG_03476	1.88	7.88E-07	1.70	0.00517	conserved hypothetical protein	
CIMG_03487	2.78	6.81E-13	2.24	1.02E-06	conserved hypothetical protein	
CIMG_03489	2.05	2.66E-06	2.66	1.42E-09	aldose 1-epimerase	
CIMG_03490	10.53	1.05E-18	2.60	2.06E-06	conserved hypothetical protein	
CIMG_03496	1.44	0.01849	1.78	0.00191	ATP-dependent rRNA helicase SPB4	
CIMG_03512	2.02	6.47E-08	1.54	0.00791	short-chain dehydrogenase/reductase	
CIMG_03517	12.66	2.02E-92	12.23	4.79E-42	conserved hypothetical protein	
CIMG_03529	2.22	7.79E-11	4.00	7.47E-16	N-acetylglucosamine-phosphate mutase	
CIMG_03530	1.59	0.00154	1.49	0.01380	Mn2+ homeostasis protein	*
CIMG_03540	6.51	1.26E-28	8.86	2.85E-29	conserved hypothetical protein	
CIMG_03564	1.93	6.49E-06	1.56	0.01284	HEC/Ndc80p family protein	
CIMG_03574	1.60	0.00040	1.78	0.00248	tubulin-tyrosine ligase	
CIMG_03576	1.64	0.00030	1.88	0.00013	Ccr4-Not transcription complex subunit	**S**
CIMG_03579	2.24	5.82E-06	2.81	5.09E-08	DASH complex subunit Duo1	
CIMG_03582	2.52	6.38E-08	1.55	0.01399	conserved hypothetical protein (Ydr279p protein family)	**+**
CIMG_03584	1.84	0.00002	2.29	0.00003	acetolactate synthase	
CIMG_03589	1.33	0.03475	1.62	0.00472	stress response protein nst1	
CIMG_03597	1.66	0.00149	1.57	0.01097	conserved hypothetical protein	
CIMG_03602	3.33	1.66E-19	2.69	8.45E-09	protein serine/threonine kinase	
CIMG_03615	2.07	1.45E-08	1.40	0.04007	conserved hypothetical protein	
CIMG_03620	1.41	0.01760	1.67	0.01909	nuclear transport factor 2 domain	
CIMG_03623	1.67	0.00008	1.50	0.01359	uncharacterized conserved protein CG6151-P (putative clathrin-coated vesicle protein)	
CIMG_03628	1.57	0.00047	1.38	0.02523	glutamate carboxypeptidase Tre2	
CIMG_03635	3.98	6.70E-28	4.41	3.33E-12	hydroxymethylglutaryl-CoA synthase Erg13	
CIMG_03637	2.80	7.12E-06	2.02	0.00175	conserved hypothetical protein	
CIMG_03644	3.73	2.55E-26	36.15	2.08E-58	galactose-proton symporter	
CIMG_03659	2.14	1.74E-07	1.84	0.00013	conserved hypothetical protein	
CIMG_03661	1.58	0.00200	1.71	0.01034	conserved hypothetical protein	
CIMG_03671	1.77	0.00035	1.70	0.00359	conserved hypothetical protein	
CIMG_03672	3.74	7.68E-23	2.34	1.14E-06	lipase/esterase	
CIMG_03680	3.14	3.14E-11	4.08	8.59E-17	conserved hypothetical protein	
CIMG_03690	2.02	2.10E-07	1.83	0.00049	Conserved hypothetical protein (ARID/BRIGHT DNA binding domain)	
CIMG_03694	2.53	2.22E-11	3.54	3.15E-10	conserved hypothetical protein	
CIMG_03710	1.46	0.00239	2.08	0.00002	ankyrin repeat protein	
CIMG_03716	8.89	7.20E-73	9.34	3.20E-25	heat shock protein 30	
CIMG_03723	1.91	8.70E-07	1.42	0.02234	C6 transcription factor	
CIMG_03725	2.06	6.06E-08	3.61	8.51E-12	zinc finger transcription factor pacC	
CIMG_03744	1.83	7.56E-06	3.68	4.59E-13	conserved hypothetical protein	
CIMG_03763	3.30	1.80E-11	1.96	0.00003	conserved hypothetical protein	
CIMG_03772	4.11	7.07E-19	1.85	0.00302	conserved hypothetical protein	
CIMG_03776	1.31	0.04773	2.56	7.30E-08	glutamyl-tRNA synthetase	
CIMG_03777	2.74	2.18E-13	1.90	0.00003	glutaminase	*
CIMG_03783	1.84	1.69E-06	2.41	1.10E-06	peroxin 19	
CIMG_03786	3.07	1.49E-19	5.15	1.22E-16	conserved hypothetical protein	*
CIMG_03793	1.44	0.00421	1.57	0.00112	SNARE-dependent exocytosis protein	**S**
CIMG_03795	2.14	1.09E-08	1.87	0.00005	mRNA 3'-end-processing protein rna14	
CIMG_03798	1.53	0.00116	1.33	0.01417	transcriptional activator spt7	
CIMG_03813	1.45	0.02194	2.47	9.06E-07	conserved hypothetical protein	*
CIMG_03820	4.71	1.57E-20	2.81	3.01E-06	conserved hypothetical protein (zinc finger)	
CIMG_03824	6.75	8.61E-56	2.94	6.12E-10	MFS peptide transporter Ptr2	
CIMG_03830	2.19	2.23E-09	1.51	0.03943	conserved hypothetical protein (DUF2373)	
CIMG_03840	17.57	3.82E-89	3.39	4.86E-07	beta-lactamase	
CIMG_03841	2.22	8.97E-11	2.13	7.42E-06	integral membrane protein	*
CIMG_03846	2.73	2.69E-10	4.06	1.79E-15	conserved hypothetical protein (MYND finger)	**G**
CIMG_03848	1.66	0.00005	10.14	7.06E-32	conserved hypothetical protein (DnaJ domain)	
CIMG_03870	20.47	3.12E-80	180.55	3.06E-69	hypothetical protein	*
CIMG_03879	3.80	1.12E-21	3.37	9.24E-12	conserved hypothetical protein	
CIMG_03884	5.58	1.52E-12	3.46	1.26E-08	conserved hypothetical protein	
CIMG_03887	1.70	0.00004	6.82	8.38E-32	tripeptidyl peptidase SED3	*
CIMG_03888	4.37	9.54E-29	16.28	2.78E-38	beta-glucosidase	*
CIMG_03906	3.07	7.17E-12	2.54	3.80E-07	conserved hypothetical protein (putative ferric reductase-like transmembrane component)	
CIMG_03910	1.88	0.00001	1.63	0.00768	capsule-associated protein CAP1	*
CIMG_03912	1.79	8.94E-06	2.86	3.78E-10	cysteine dioxygenase	
CIMG_03917	1.83	0.00508	7.64	2.07E-13	conserved hypothetical protein (CENP-B N-terminal DNA-binding domain, putative centromere binding protein B)	

*: Predicted secreted;
+: Gene under positive selection [19]; G: Gene gained in Coccidioides [19]
H, S: Up-regulated in Hyphae or Spherule by microarray [18]

Table S1: All genes up-regulated in saprobic phase

CIMG_03953	1.69	0.00011	2.04	0.00087	aureobasidin resistance protein Aur1	
CIMG_03955	15.38	8.55E-94	2.46	0.00251	conserved hypothetical protein (WW domain)	
CIMG_03981	2.84	1.47E-15	4.99	5.45E-25	neutral/alkaline nonlysosomal ceramidase	*
CIMG_03987	2.69	4.44E-10	2.81	0.00019	hypothetical protein	
CIMG_03989	3.71	3.09E-06	1.61	0.01054	uncleaved alkaline protease	*
CIMG_04010	1.60	0.00025	2.84	9.37E-10	metacaspase CasA	
CIMG_04011	1.76	0.00006	1.51	0.00842	leucine Rich Repeat domain-containing protein	
CIMG_04013	2.17	3.15E-10	2.53	1.87E-07	CBS and PB1 domain-containing protein	
CIMG_04014	1.38	0.02179	1.87	0.00062	importin beta-3 subunit	*
CIMG_04015	4.98	2.38E-40	3.54	3.40E-13	isoflavone reductase	
CIMG_04027	1.89	7.87E-07	2.15	4.80E-07	serine/threonine-protein kinase ssp1	+
CIMG_04028	3.46	2.48E-17	2.09	6.23E-06	conserved hypothetical protein	
CIMG_04032	1.44	0.02209	2.06	0.00004	conserved hypothetical protein (DUF2349)	
CIMG_04038	1.75	0.00002	2.24	0.00007	casein kinase I isoform gamma-1	
CIMG_04062	2.36	1.81E-07	2.76	0.00005	5-methyltetrahydropteroyltriglutamate-homocysteine methyltransferase	
CIMG_04070	10.78	2.97E-42	7.19	4.66E-17	conserved hypothetical protein	*
CIMG_04072	2.57	1.86E-09	2.23	0.00004	cAMP-mediated signaling protein Sok1	
CIMG_04075	3.86	4.84E-11	7.62	9.89E-14	conserved hypothetical protein	
CIMG_04084	2.39	1.32E-11	2.25	0.00002	ornithine carbamoyltransferase	
CIMG_04099	1.38	0.01585	2.18	0.00004	conserved hypothetical protein	*
CIMG_04101	2.35	1.23E-08	2.47	1.63E-06	conserved hypothetical protein	
CIMG_04102	2.09	8.52E-08	1.40	0.02616	dihydrofolate synthetase Fol3	
CIMG_04103	3.28	7.53E-20	2.58	2.02E-08	serine/threonine protein kinase 3 (cell division control protein 15)	
CIMG_04117	1.81	3.52E-06	1.82	0.00004	conserved hypothetical protein (YCII-related domain)	
CIMG_04126	1.40	0.03762	2.14	0.00023	methionine aminopeptidase 2B	
CIMG_04135	1.35	0.03430	1.77	0.00090	amino acid transporter	
CIMG_04157	1.94	2.22E-07	2.33	0.00016	killer toxin sensitivity protein	
CIMG_04163	13.05	4.04E-64	24.54	1.83E-64	ras guanine-nucleotide exchange protein	
CIMG_04166	10.87	6.45E-83	8.92	2.63E-32	conserved hypothetical protein	
CIMG_04173	1.51	0.00471	2.02	1.03E-06	pH signal transduction protein PalA	
CIMG_04186	6.34	2.33E-28	15.68	3.52E-48	conserved hypothetical protein	
CIMG_04188	3.52	1.08E-23	13.65	2.85E-39	GABA permease	H
CIMG_04189	2.27	0.00991	3.90	0.00002	conserved hypothetical protein	
CIMG_04202	2.41	8.68E-10	2.32	1.68E-08	protein kinase Scy1	
CIMG_04211	1.91	1.16E-06	1.70	0.00108	RSC complex subunit Sfh1	
CIMG_04218	1.47	0.00685	1.67	0.00127	GCN20 (ABC transporter)	
CIMG_04220	2.01	1.42E-06	1.98	0.01252	MFS multidrug transporter	
CIMG_04222	1.84	3.83E-06	4.20	3.19E-13	conserved hypothetical protein	
CIMG_04230	1.33	0.04958	1.27	0.03793	JmjC domain-containing protein	
CIMG_04239	1.94	7.85E-06	1.97	0.00080	hypothetical protein (RNA recognition motif)	
CIMG_04272	1.63	0.00011	1.60	0.00172	peroxisomal targeting signal-1 receptor	
CIMG_04305	1.50	0.00255	1.43	0.01235	2-hydroxyphytanoyl-CoA lyase	
CIMG_04307	1.61	0.00035	1.39	0.03856	RNA polymerase II subunit 7	
CIMG_04308	2.06	6.41E-07	3.30	1.60E-11	Conserved hypothetical protein (DUF962)	
CIMG_04311	1.91	1.50E-06	1.43	0.03429	signal peptidase complex component (microsomal signal peptidase 25 kDa subunit)	
CIMG_04317	13.21	2.55E-92	7.54	4.02E-27	extracellular matrix protein	* H
CIMG_04319	3.18	1.29E-13	1.64	0.00713	ribosome biogenesis protein Noc4	
CIMG_04335	2.11	0.00005	7.09	1.81E-15	conserved hypothetical protein	
CIMG_04343	1.38	0.01334	1.62	0.00288	cytosolic regulator Pianissimo	S
CIMG_04371	3.83	7.41E-15	3.28	7.56E-09	conserved hypothetical protein	
CIMG_04372	2.23	1.54E-06	4.02	2.50E-12	conserved hypothetical protein	
CIMG_04381	2.32	2.95E-09	1.87	0.00015	conserved hypothetical protein	
CIMG_04401	4.67	8.09E-38	4.58	7.45E-18	hexokinase-1	
CIMG_04414	1.60	0.00050	2.28	0.00007	n-acetylglucosamine-6-phosphate deacetylase	
CIMG_04416	2.38	7.80E-13	10.42	1.89E-36	carboxypeptidase 2	*
CIMG_04417	6.20	1.29E-51	4.79	4.07E-21	cutinase trasnscription factor 1 alpha	
CIMG_04443	2.31	9.92E-10	1.96	0.00004	alpha-1,2-mannosyltransferase	*
CIMG_04451	2.02	8.30E-09	3.44	1.12E-14	cystathionine gamma-synthase	
CIMG_04460	12.32	2.73E-85	2.99	1.13E-10	acetamidase	
CIMG_04462	1.75	0.00003	1.46	0.04420	splicing factor U2AF 35 kDa subunit	
CIMG_04464	2.75	7.67E-12	8.66	3.56E-29	conserved hypothetical protein	*
CIMG_04468	9.57	5.40E-75	2.84	4.07E-09	conserved hypothetical protein (putative short chain dehydrogenase)	
CIMG_04470	1.89	0.00003	1.97	0.00293	membrane bound C2 domain-containing protein	S
CIMG_04476	2.76	2.23E-11	3.22	6.76E-08	histone deacetylase phd1	
CIMG_04479	4.68	1.58E-35	2.13	0.00003	isochorismatase family hydrolase	
CIMG_04481	3.50	1.15E-16	2.28	2.07E-06	transcription/RNA-processing factor Ssu72	
CIMG_04487	2.88	1.42E-17	3.36	4.86E-13	cell polarity protein	
CIMG_04488	2.01	1.50E-07	2.20	0.00015	HMG-CoA reductase	
CIMG_04495	4.00	2.18E-30	2.27	2.15E-06	ras GTPase activating protein	

*: Predicted secreted;
+: Gene under positive selection [19]; G: Gene gained in Coccidioides [19]
H, S: Up-regulated in Hyphae or Spherule by microarray [18]

Table S1: All genes up-regulated in saprobic phase

CIMG_04497	2.28	1.14E-06	12.44	1.75E-40	conserved hypothetical protein	*
CIMG_04498	7.71	8.38E-47	2.31	0.00003	NUDIX family hydrolase	
CIMG_04500	1.40	0.01308	1.55	0.02020	phenylalanyl-tRNA synthetase, subunit alpha	
CIMG_04501	2.82	8.56E-16	2.75	1.99E-10	sugar transporter	
CIMG_04506	1.98	0.00007	2.48	4.04E-08	amino acid permease	
CIMG_04518	4.10	1.99E-13	18.78	4.90E-55	leucine aminopeptidase 2	* H
CIMG_04530	1.53	0.00478	3.55	1.39E-13	conserved hypothetical protein	*
CIMG_04554	2.96	1.09E-18	2.17	0.00001	kinesin family protein	
CIMG_04573	1.54	0.00079	1.70	0.00107	pre-mRNA splicing factor	
CIMG_04597	5.47	1.67E-44	1.61	0.00391	putative GPI-anchored serine-threonine rich protein	*
CIMG_04608	1.80	5.07E-06	6.22	1.54E-30	MFS transporter	
CIMG_04618	4.53	5.56E-22	1.50	0.00248	conserved hypothetical protein	
CIMG_04622	3.11	2.11E-20	2.98	3.47E-09	CCAAT binding protein HAPC	
CIMG_04631	1.74	0.00002	3.50	9.35E-11	calmodulin-dependent protein kinase	
CIMG_04642	7.13	2.96E-32	27.77	1.48E-62	C2H2 zinc finger domain-containing protein FlbC	
CIMG_04649	4.40	1.82E-35	2.55	6.71E-08	succinate-semialdehyde dehydrogenase Uga2	
CIMG_04651	1.98	1.01E-07	3.84	5.90E-08	short chain dehydrogenase	
CIMG_04655	2.34	6.62E-09	5.74	3.31E-21	conserved hypothetical protein	
CIMG_04660	3.49	4.29E-22	3.93	1.14E-11	cellular morphogenesis protein	*
CIMG_04665	5.11	8.02E-40	2.52	9.20E-07	conserved hypothetical protein	*
CIMG_04672	3.26	0.00013	2.84	0.00003	conserved hypothetical protein (SET domain)	
CIMG_04675	2.11	6.09E-09	2.71	7.55E-11	conserved hypothetical protein	
CIMG_04701	7.19	1.42E-11	1.86	0.00603	conserved hypothetical protein	
CIMG_04706	1.90	0.00151	1.85	0.00021	conserved hypothetical protein	G
CIMG_04712	1.97	1.45E-06	2.41	2.89E-06	conserved hypothetical protein (peptidase family C50)	
CIMG_04720	1.65	0.00071	2.33	7.10E-07	conserved hypothetical protein	
CIMG_04729	1.97	0.00003	2.12	0.00111	heat shock protein	
CIMG_04735	1.83	3.61E-06	6.32	2.12E-21	conserved hypothetical protein	
CIMG_04747	1.36	0.03273	1.74	0.00137	cell cycle inhibitor Nif1	
CIMG_04749	4.50	2.84E-34	4.37	1.73E-20	conserved hypothetical protein	
CIMG_04750	1.89	1.02E-06	1.73	0.00103	class V chitinase	
CIMG_04769	1.86	4.33E-07	1.68	0.00190	F-actin capping protein beta subunit	
CIMG_04782	1.46	0.00547	3.38	2.02E-13	conserved hypothetical protein	
CIMG_04790	2.04	2.17E-06	1.82	0.00125	phosphoribosylglycinamide formyltransferase	
CIMG_04792	2.37	4.30E-10	2.07	0.00002	dUTPase	
CIMG_04797	1.97	3.20E-08	6.78	6.88E-24	cystinosin	
CIMG_04798	1.96	5.63E-08	3.83	1.35E-17	conserved hypothetical protein	
CIMG_04804	1.85	4.13E-06	1.62	0.00018	MYB family conidiophore development protein FlbD	
CIMG_04844	2.79	2.34E-11	2.69	1.53E-07	endo-1,3(4)-beta-glucanase	*
CIMG_04846	1.55	0.00086	1.73	0.00025	golgi complex component	
CIMG_04887	2.70	3.24E-14	2.39	1.25E-06	mitochondrial protein import protein MAS5	
CIMG_04899	1.74	0.00018	3.62	3.89E-13	leucine Rich Repeat domain-containing protein	
CIMG_04900	11.35	1.63E-71	13.70	6.99E-45	conserved hypothetical protein	
CIMG_04906	1.60	0.00040	1.82	0.00034	protein phosphatase type 1b	
CIMG_04911	1.92	2.61E-06	3.14	2.64E-11	conserved hypothetical protein	*
CIMG_04918	2.50	3.00E-13	2.27	1.77E-06	phosphoprotein phosphatase	
CIMG_04934	6.88	4.11E-44	4.32	1.51E-12	conserved hypothetical protein (HMG box)	
CIMG_04945	6.13	1.04E-34	11.10	1.52E-20	alcohol dehydrogenase	
CIMG_04966	1.84	0.00290	1.48	0.01375	conserved hypothetical protein	*
CIMG_04967	4.68	3.88E-31	14.66	1.99E-43	conserved hypothetical protein	*
CIMG_04968	3.55	3.55E-11	2.76	2.21E-06	short-chain dehydrogenase	
CIMG_04969	1.83	0.00096	1.74	0.00453	conserved hypothetical protein	
CIMG_04972	1.91	3.98E-07	1.29	0.00718	cytochrome P450 monooxygenase	*
CIMG_04973	2.89	1.02E-14	1.74	0.00201	F-box domain-containing protein	
CIMG_04976	2.60	8.69E-11	2.92	1.24E-10	hypothetical protein (SH3 domain)	S
CIMG_04978	1.76	0.00005	5.06	1.62E-22	chitobiase	* H
CIMG_04998	1.62	0.00444	1.53	0.04529	conserved hypothetical protein	
CIMG_05030	1.53	0.00117	1.87	0.00011	dynactin	
CIMG_05057	4.00	3.71E-28	2.31	0.00083	conserved hypothetical protein	*
CIMG_05088	1.57	0.00083	3.23	3.19E-13	tryptophanyl-tRNA synthetase	
CIMG_05092	5.58	1.68E-31	4.86	8.28E-15	conserved hypothetical protein	+
CIMG_05093	2.65	1.24E-15	2.71	2.38E-07	meiosis induction protein kinase	
CIMG_05097	3.73	5.28E-26	4.73	4.74E-17	conserved hypothetical protein	
CIMG_05099	2.45	3.56E-10	5.59	6.11E-22	conserved hypothetical protein	*
CIMG_05106	1.90	7.29E-07	2.89	4.32E-12	conserved hypothetical protein (NCS1 nucleoside transporter family)	
CIMG_05121	3.39	4.45E-18	2.06	0.00007	peroxin 14/17	
CIMG_05168	6.65	1.41E-08	2.43	0.00901	hemolysin-III channel protein Izh2	
CIMG_05173	5.06	5.19E-41	4.54	5.42E-19	conserved hypothetical protein	
CIMG_05196	3.88	3.55E-24	2.07	0.00087	carnitine acetyl transferase	
CIMG_05201	2.87	2.25E-17	1.59	0.00456	BTN1 (major facilitator superfamily)	

*: Predicted secreted;
+: Gene under positive selection [19]; G: Gene gained in Coccidioides [19]
H, S: Up-regulated in Hyphae or Spherule by microarray [18]

Table S1: All genes up-regulated in saprobic phase

CIMG_05219	1.93	2.83E-06	1.65	0.00375	conserved hypothetical protein (WD domain)	
CIMG_05224	1.79	4.34E-06	2.89	2.83E-10	integral membrane protein	
CIMG_05233	5.98	8.51E-21	7.68	7.56E-21	conserved hypothetical protein	
CIMG_05234	5.97	1.44E-43	5.13	1.09E-13	septin AspB	
CIMG_05239	7.89	3.17E-45	6.51	1.44E-19	conserved hypothetical protein	
CIMG_05251	2.21	3.66E-06	1.85	0.00397	transcription factor Tos4	
CIMG_05254	34.15	6.56E-139	48.09	2.83E-56	cell wall synthesis protein	*
CIMG_05274	2.20	7.08E-09	9.43	3.09E-44	conserved hypothetical protein	*
CIMG_05276	2.10	1.70E-08	2.07	3.59E-06	serine/threonine-protein kinase RIO1	S
CIMG_05279	1.55	0.00126	1.64	0.00442	NADH-cytochrome b5 reductase 1	
CIMG_05280	1.54	0.00077	2.03	0.00015	arginyl-tRNA synthetase	
CIMG_05282	3.69	1.05E-16	10.34	3.12E-30	conserved hypothetical protein	
CIMG_05283	4.88	4.96E-32	11.51	8.66E-44	conserved hypothetical protein	
CIMG_05284	6.22	5.43E-51	10.09	7.40E-40	conserved hypothetical protein	
CIMG_05286	1.62	0.04644	1.52	0.02780	conserved hypothetical protein	
CIMG_05363	1.69	0.00077	7.46	3.25E-22	conserved hypothetical protein	
CIMG_05372	2.59	5.03E-13	1.62	0.02288	EBP domain-containing protein	
CIMG_05404	2.40	3.25E-08	2.91	1.98E-08	conserved hypothetical protein	*
CIMG_05412	1.74	0.00002	4.74	9.71E-21	conserved hypothetical protein (phosphotransferase enzyme family)	
CIMG_05420	2.25	7.82E-10	2.32	8.45E-06	conserved hypothetical protein	*
CIMG_05427	1.85	1.13E-06	4.10	1.54E-17	transcriptional corepressor Cyc8	
CIMG_05428	2.64	8.87E-07	3.62	6.25E-09	conserved hypothetical protein	
CIMG_05429	4.08	6.48E-31	5.87	4.72E-28	conserved hypothetical protein	
CIMG_05433	2.06	9.19E-08	1.80	0.00003	dicer-like protein 2	
CIMG_05439	1.73	0.00041	2.33	1.34E-06	conserved hypothetical protein (fungal specific transcription factor domain)	
CIMG_05458	1.42	0.00856	1.56	0.01055	guanyl-nucleotide exchange factor	
CIMG_05472	1.59	0.00112	1.35	0.04737	APSES transcription factor	
CIMG_05486	3.12	1.20E-15	1.52	0.00713	HEAT repeat protein	
CIMG_05497	2.65	5.86E-14	1.95	0.00007	conserved hypothetical protein	
CIMG_05502	2.21	6.88E-10	2.20	0.00002	kinesin motor domain-containing protein	
CIMG_05518	5.42	3.45E-43	2.91	6.61E-11	nuclear condensin complex subunit Smc4	
CIMG_05523	8.02	6.97E-31	7.15	1.16E-21	conserved hypothetical protein	*
CIMG_05525	2.98	7.12E-15	2.69	2.64E-08	exonuclease	
CIMG_05528	2.04	0.00001	2.46	5.70E-06	X-Pro dipeptidyl-peptidase	+
CIMG_05536	1.86	8.89E-07	1.33	0.04468	oxidoreductase	
CIMG_05544	2.03	2.97E-07	1.66	0.00605	conserved hypothetical protein	
CIMG_05558	1.55	0.00174	1.99	0.00020	conserved hypothetical protein	
CIMG_05570	15.46	4.31E-102	3.70	5.54E-10	FAD binding domain-containing protein	
CIMG_05586	1.77	0.00004	6.31	1.15E-24	methylated-DNA-protein-cysteine methyltransferase	
CIMG_05593	2.57	6.33E-13	2.33	6.46E-07	conserved hypothetical protein (MRC1-like domain)	
CIMG_05597	9.86	2.29E-78	4.18	2.74E-17	FAD-dependent oxygenase	*
CIMG_05604	1.47	0.00441	1.73	0.00161	phytanoyl-CoA dioxygenase	
CIMG_05619	11.12	2.17E-61	6.34	2.53E-16	conserved hypothetical protein	
CIMG_05620	1.92	4.97E-07	5.38	1.94E-18	conserved hypothetical protein	
CIMG_05635	2.11	3.38E-09	2.59	4.48E-08	HORMA domain-containing protein	
CIMG_05637	3.79	1.90E-28	2.77	1.88E-07	conserved hypothetical protein	
CIMG_05638	2.35	7.77E-12	2.41	3.86E-07	conserved hypothetical protein	
CIMG_05642	1.52	0.01227	3.13	3.28E-08	pyridoxal-phosphate dependent enzyme	
CIMG_05647	4.02	3.05E-29	4.18	1.01E-12	class III chitin synthase	
CIMG_05734	11.94	7.83E-79	123.67	4.29E-88	conserved hypothetical protein	
CIMG_05771	7.05	1.01E-33	2.65	1.96E-07	MFS multidrug transporter	
CIMG_05794	5.17	1.72E-41	10.50	2.72E-36	conserved hypothetical protein	
CIMG_05797	1.74	0.00002	1.49	0.01182	tRNA-splicing endonuclease	
CIMG_05801	1.35	0.04093	2.03	0.00002	DUF803 domain-containing protein	
CIMG_05818	1.44	0.01216	8.51	1.77E-25	GNAT family acetyltransferase	
CIMG_05832	1.42	0.01025	1.71	0.00104	conserved hypothetical protein (putative C2HC5 type zinc finger motif)	
CIMG_05833	1.69	0.00066	2.41	5.80E-07	conserved hypothetical protein	
CIMG_05862	1.41	0.00965	1.64	0.00426	ubiquitin C-terminal hydrolase	
CIMG_05879	1.72	0.00020	4.70	8.85E-14	conserved hypothetical protein	
CIMG_05881	1.74	0.00048	7.06	1.85E-16	conserved hypothetical protein	*
CIMG_05886	1.35	0.02646	1.62	0.01701	tubulin gamma chain	
CIMG_05888	1.68	0.00005	4.02	3.46E-14	mannose-6-phosphate isomerase	
CIMG_05895	1.35	0.02515	1.40	0.04813	actin	
CIMG_05946	1.90	3.07E-06	9.00	3.18E-33	methyltransferase small domain-containing protein	
CIMG_05947	7.64	1.96E-46	6.80	2.47E-21	cell division control protein 14	
CIMG_05949	6.66	5.50E-25	14.76	3.88E-50	sugar transporter	
CIMG_05950	1.52	0.00104	4.50	1.03E-16	geranylgeranyl pyrophosphate synthetase	
CIMG_05954	4.83	1.98E-12	1.77	0.00244	tRNA a64-2'-O-ribosylphosphate transferase	
CIMG_05955	10.53	4.22E-68	3.88	5.57E-15	HAD superfamily hydrolase	
CIMG_05956	2.06	8.47E-06	5.74	3.59E-25	conserved hypothetical protein	*

*: Predicted secreted;
+: Gene under positive selection [19]; G: Gene gained in Coccidioides [19]
H, S: Up-regulated in Hyphae or Spherule by microarray [18]

Table S1: All genes up-regulated in saprobic phase

CIMG_05958	1.36	0.02539	2.04	0.00009	MutT/nudix family protein	
CIMG_05962	3.08	5.02E-18	13.79	2.30E-31	conserved hypothetical protein	
CIMG_05968	2.81	1.80E-15	1.84	0.00012	dolichyl-phosphate beta-glucosyltransferase	
CIMG_05975	12.99	9.89E-08	2.32	0.04669	conserved hypothetical protein (putative hydantoinase B/oxoprolinase)	
CIMG_05981	2.89	5.14E-17	2.08	0.00005	K+ homeostasis protein Kha1	
CIMG_05982	2.32	1.71E-11	1.65	0.00695	peroxin 3	
CIMG_05985	2.83	5.67E-13	3.11	4.28E-10	RfxA	
CIMG_05992	1.58	0.00990	1.53	0.00793	conserved hypothetical protein	
CIMG_05993	1.78	0.00007	2.52	1.57E-07	DUF89 domain-containing protein	
CIMG_05994	2.36	9.14E-12	2.80	1.73E-09	serine/threonine protein phosphatase PPT1	
CIMG_06003	1.73	0.00061	1.54	0.00542	SNF2 family helicase/ATPase	
CIMG_06012	1.78	0.00005	1.71	0.00028	C2H2 transcription factor	
CIMG_06031	1.55	0.00086	1.51	0.03416	adenosine kinase	
CIMG_06036	13.32	3.81E-94	4.87	4.22E-24	DNA repair and transcription factor Ada	**H**
CIMG_06043	1.92	1.84E-06	1.91	0.00006	RSC complex subunit	
CIMG_06051	1.59	0.00048	1.49	0.01243	sucrose transporter	
CIMG_06070	2.44	1.77E-12	1.54	0.00870	homoserine O-acetyltransferase	
CIMG_06092	4.08	1.99E-17	2.31	0.00012	O-methyltransferase	**+ G H**
CIMG_06109	2.03	1.40E-08	1.78	0.00040	CCR4-NOT transcription complex subunit 7	
CIMG_06113	13.10	1.90E-95	2.41	8.53E-06	phenylacetyl-CoA ligase	
CIMG_06116	2.08	9.78E-09	2.17	1.21E-06	GTPase activating protein	
CIMG_06133	1.49	0.02062	1.84	0.00073	conserved hypothetical protein (DUF2439)	
CIMG_06134	2.56	5.51E-14	2.69	2.87E-08	conserved hypothetical protein (methyltransferase domain)	
CIMG_06135	7.47	1.17E-59	4.41	6.23E-14	UMTA methyltransferase	
CIMG_06137	19.73	5.10E-117	5.51	8.05E-26	conserved hypothetical protein (Cytochrome P450)	*
CIMG_06153	1.98	2.34E-07	2.77	1.11E-08	putative membrane protein (DUF914)	
CIMG_06164	2.87	1.45E-10	5.15	6.09E-14	conserved hypothetical protein	
CIMG_06167	1.64	0.00021	2.10	0.00005	cadmium factor	
CIMG_06170	8.39	1.75E-67	8.74	1.12E-33	conserved hypothetical protein	
CIMG_06181	3.58	2.55E-22	3.41	5.31E-13	pyridoxine biosynthesis protein PDX1	
CIMG_06186	10.76	5.94E-83	43.14	1.25E-67	lysine amidinotransferase	
CIMG_06188	10.48	2.31E-50	3.13	4.06E-07	cytochrome P450 monooxygenase	
CIMG_06190	1.72	0.00004	1.52	0.02227	arginine biosynthesis protein ArgJ	
CIMG_06201	1.78	0.00012	2.61	1.05E-08	alpha 1,2-mannosyltransferase Smp3	*
CIMG_06209	2.00	9.96E-08	2.04	0.00205	translation initiation factor eIF-6	
CIMG_06213	1.91	3.44E-07	1.73	0.00586	conserved hypothetical protein	
CIMG_06224	3.92	3.64E-23	1.50	0.01268	RNA processing factor 1	
CIMG_06225	1.85	2.03E-06	1.55	0.02791	glycyl-tRNA synthetase	
CIMG_06227	1.36	0.02665	1.70	0.00176	serine/threonine-protein kinase CLA4	**S**
CIMG_06245	1.51	0.00257	3.67	7.26E-14	conserved hypothetical protein	
CIMG_06255	2.05	2.70E-08	1.68	0.00312	RuvB-like helicase 2	
CIMG_06267	4.83	4.26E-32	6.84	5.60E-32	conserved hypothetical protein	
CIMG_06288	1.85	0.00095	1.94	0.04795	plasma membrane ATPase	
CIMG_06293	2.04	1.01E-07	1.87	3.02E-06	conserved hypothetical protein (RNA recognition motif)	
CIMG_06301	9.99	2.08E-66	2.91	3.09E-08	conserved hypothetical protein (DUF2503)	
CIMG_06323	1.91	0.00078	2.32	3.38E-06	pumilio-family RNA binding repeat protein	
CIMG_06324	2.07	1.95E-07	1.90	0.00003	conserved hypothetical protein	
CIMG_06335	4.13	3.28E-14	2.15	0.00078	conserved hypothetical protein	
CIMG_06344	34.21	2.08E-139	71.53	2.08E-89	conserved hypothetical protein	
CIMG_06347	1.69	0.00028	2.13	2.49E-07	GTP cyclohydrolase II	
CIMG_06348	1.57	0.00135	1.45	0.03992	transcription elongation factor 1	
CIMG_06352	1.55	0.00039	1.96	0.00005	seryl-tRNA synthetase	
CIMG_06381	1.42	0.00905	1.63	0.00101	conserved hypothetical protein	
CIMG_06386	1.52	0.00159	2.24	1.35E-06	conserved hypothetical protein (Myb-like DNA-binding domain)	
CIMG_06397	2.02	8.06E-08	2.09	0.00003	Conserved hypothetical protein (RNA recognition motif, R3H domain)	
CIMG_06408	9.72	4.00E-74	2.08	0.00014	monosaccharide transporter	
CIMG_06422	4.65	8.91E-38	1.66	0.01015	AMP dependent CoA ligase	
CIMG_06431	1.92	2.63E-07	1.58	0.01119	nuclear condensin complex subunit 3	
CIMG_06436	5.24	6.89E-24	3.04	2.03E-10	conserved hypothetical protein	
CIMG_06453	1.93	1.63E-07	2.02	0.00011	farnesyl-pyrophosphate synthetase	
CIMG_06457	7.83	8.38E-25	2.58	2.95E-06	conserved hypothetical protein	
CIMG_06465	1.51	0.00167	1.74	0.00125	histone acetylase complex subunit Paf400	
CIMG_06469	11.30	4.60E-73	1.65	0.00346	MFS multidrug transporter	
CIMG_06480	2.10	7.75E-07	1.63	0.01098	conserved hypothetical protein (DUF1996)	*
CIMG_06488	1.54	0.00228	1.51	0.04059	NAP family protein	
CIMG_06501	3.52	0.00156	3.79	0.03583	hypothetical protein	
CIMG_06502	3.16	4.67E-22	1.58	0.02360	conserved hypothetical protein	*
CIMG_06506	3.30	6.21E-08	4.10	3.44E-11	conserved hypothetical protein	
CIMG_06510	4.19	5.41E-31	3.26	1.17E-09	Sep5a (septin)	
CIMG_06514	2.11	1.54E-09	2.11	3.67E-06	exonuclease Kem1	

*: Predicted secreted;
+: Gene under positive selection [19]; G: Gene gained in Coccidioides [19]
H, S: Up-regulated in Hyphae or Spherule by microarray [18]

<u>Table S1</u>: All genes up-regulated in saprobic phase

CIMG_06524	1.82	0.00001	5.63	1.09E-26	conserved hypothetical protein (Dynamin family)	
CIMG_06535	1.54	0.00431	1.82	0.00039	conserved hypothetical protein	
CIMG_06537	1.46	0.00667	1.56	0.04733	conserved hypothetical protein (emp24/gp25L/p24 family)	*
CIMG_06540	3.37	7.73E-15	2.56	2.00E-08	conserved hypothetical protein	
CIMG_06543	2.86	1.03E-13	2.52	5.80E-08	microsomal signal peptidase subunit	*
CIMG_06553	4.77	1.20E-32	4.46	6.34E-20	putative SH3 domain protein Cyk3	
CIMG_06555	1.58	0.00059	1.49	0.03293	PHD transcription factor	
CIMG_06558	1.87	0.00014	15.46	1.50E-48	conserved hypothetical protein (major facilitator superfamily)	
CIMG_06560	1.64	0.00028	1.49	0.01308	methylenetetrahydrofolate reductase	
CIMG_06563	1.86	1.19E-06	1.69	0.00099	alpha glucosidase II	*
CIMG_06566	7.47	7.94E-16	4.39	3.74E-08	endo-1,3(4)-beta-glucanase	*
CIMG_06576	1.51	0.01252	2.34	3.87E-06	conserved hypothetical protein	
CIMG_06582	3.16	2.12E-18	2.69	3.44E-10	phosphatidylinositol transfer protein SFH5	
CIMG_06584	3.80	9.70E-29	4.64	1.36E-17	conserved hypothetical protein	*
CIMG_06593	4.97	5.24E-30	2.82	1.56E-08	conserved hypothetical protein	
CIMG_06597	1.55	0.00070	1.86	0.00007	signal recognition particle protein SRP54	
CIMG_06601	3.08	6.66E-21	3.31	3.24E-12	bZIP transcription factor HapX	
CIMG_06602	4.60	1.01E-30	8.51	3.47E-22	O-methyltransferase	
CIMG_06611	1.61	0.00024	1.94	0.00008	ceramide glucosyltransferase	
CIMG_06615	60.20	1.37E-141	127.92	5.63E-60	conserved hypothetical protein (putative fungal hydrophobin)	*
CIMG_06623	3.18	2.24E-20	2.42	1.97E-06	ABC transporter	
CIMG_06625	5.91	1.74E-13	2.81	2.36E-06	conserved hypothetical protein	
CIMG_06631	4.63	6.54E-14	15.30	5.59E-35	zinc binding enoyl reductase	
CIMG_06637	2.11	2.74E-09	2.18	4.41E-06	aspartyl aminopeptidase	
CIMG_06654	1.69	0.00011	3.95	3.77E-17	DUF1688 domain-containing protein	
CIMG_06655	1.81	0.00004	1.53	0.00240	small monomeric GTPase	
CIMG_06667	2.19	1.23E-09	3.37	3.11E-12	serine/threonine protein phosphatase 2B catalytic subunit	
CIMG_06674	1.45	0.00871	1.51	0.00249	beige/BEACH domain-containing protein	
CIMG_06681	2.34	9.49E-12	2.39	7.94E-11	DUF1620 domain-containing protein	*
CIMG_06685	1.87	7.05E-07	1.54	0.00853	Transmembrane amino acid transporter	
CIMG_06690	1.52	0.00198	1.58	0.00235	ATP-dependent bile acid permease	
CIMG_06700	3.86	2.12E-10	6.11	7.37E-14	conserved hypothetical protein	*
CIMG_06716	6.75	4.08E-54	6.68	6.40E-19	conserved hypothetical protein	
CIMG_06724	2.35	1.21E-12	2.66	7.66E-11	isovaleryl-CoA dehydrogenase	
CIMG_06727	1.58	0.00158	4.52	8.52E-12	D-arabinitol dehydrogenase ArbD	
CIMG_06738	20.54	2.00E-81	5.39	3.86E-11	woronin body major protein	H
CIMG_06741	2.01	3.93E-08	1.97	0.00002	LAP2 (peptidase family M28)	*
CIMG_06772	1.47	0.00331	1.61	0.00303	pre-mRNA-splicing factor isy1	
CIMG_06776	1.36	0.02810	1.61	0.00531	vacuolar-type H+-ATPase subunit C	
CIMG_06778	3.96	3.04E-26	4.78	3.33E-15	septin-1	
CIMG_06793	5.85	1.90E-19	2.08	0.00317	conserved hypothetical protein	
CIMG_06818	2.24	1.77E-10	1.86	0.00219	G-alpha subunit	
CIMG_06821	3.23	2.59E-16	1.59	0.03043	4-coumarate-CoA ligase	
CIMG_06823	1.42	0.00412	1.60	0.00396	nucleoporin	
CIMG_06848	1.92	1.12E-07	1.91	0.00013	STU1	
CIMG_06851	1.33	0.02802	1.75	0.00606	siderophore biosynthesis acetylase AceI	
CIMG_06852	1.47	0.00322	1.51	0.02903	ABC multidrug transporter Mdr4	
CIMG_06859	3.19	2.34E-22	1.65	0.00141	zinc metalloproteinase	
CIMG_06868	1.61	0.00877	1.70	0.00170	copper amine oxidase 1	
CIMG_06880	2.93	3.04E-14	2.22	0.00005	conserved hypothetical protein	
CIMG_06892	8.00	1.34E-24	3.24	9.67E-10	amidohydrolase	
CIMG_06928	6.13	1.68E-35	4.54	1.73E-09	tubulin beta chain	
CIMG_06932	1.84	1.45E-06	1.54	0.01046	MAP kinase kinase kinase SskB	
CIMG_06933	1.63	0.00070	2.34	7.72E-06	vesicle-mediated transporter Vid24 (vacuolar import and degradation protein)	
CIMG_06940	2.14	4.84E-09	2.05	8.27E-06	serine/threonine protein kinase Kin1	
CIMG_06966	2.69	1.00E-11	1.85	0.00116	3-ketosphinganine reductase	
CIMG_06971	2.01	5.21E-08	1.88	0.00049	conserved hypothetical protein (transmembrane protein precursor)	*
CIMG_06977	2.44	4.46E-12	1.91	0.00011	RNA export mediator Gle1	
CIMG_06978	1.45	0.00565	2.30	6.19E-07	myosin class II heavy chain	
CIMG_06981	2.00	2.60E-07	1.59	0.00834	endosomal cargo receptor (emp24/gp25L/p24 family)	*
CIMG_06985	1.95	7.72E-06	1.95	0.00138	conserved hypothetical protein	
CIMG_06988	1.42	0.01757	1.63	0.00179	conserved hypothetical protein (fungal specific transcription factor domain)	
CIMG_07006	1.83	2.25E-06	1.59	0.01279	COPII-coated vesicle protein SurF4/Erv29 (SURF4 family)	
CIMG_07037	4.90	1.23E-27	10.34	4.33E-25	conserved hypothetical protein	
CIMG_07042	2.25	8.68E-10	3.76	2.59E-10	HMG box protein	
CIMG_07049	2.72	4.22E-16	4.42	5.87E-16	NAD(P) transhydrogenase	
CIMG_07066	2.23	0.00180	10.62	8.82E-22	conserved hypothetical protein	*
CIMG_07071	2.26	2.55E-11	2.10	8.11E-06	conserved hypothetical protein	
CIMG_07077	1.65	0.01822	1.44	0.02993	conserved hypothetical protein	
CIMG_07093	2.79	1.94E-16	1.70	0.00496	exosome complex exonuclease exoribonuclease	

*: Predicted secreted;
+: Gene under positive selection [19]; G: Gene gained in Coccidioides [19]
H, S: Up-regulated in Hyphae or Spherule by microarray [18]

Table S1: All genes up-regulated in saprobic phase

CIMG_07100	1.72	0.00011	2.32	3.72E-06	PAB1 binding protein	
CIMG_07103	25.53	7.08E-68	5.44	5.60E-12	stress responsive A/B barrel domain-containing protein	
CIMG_07109	1.90	2.84E-06	1.65	0.01327	conserved hypothetical protein (putative SUR7 protein)	
CIMG_07132	2.27	1.02E-07	1.44	0.00694	conserved hypothetical protein (PPR repeat)	
CIMG_07138	1.81	7.58E-06	2.15	0.00050	conserved hypothetical protein	
CIMG_07163	1.49	0.00555	1.64	0.00321	hydrolase	
CIMG_07170	1.89	0.00004	2.61	5.42E-06	conserved hypothetical protein	
CIMG_07174	3.53	6.47E-25	3.82	1.14E-15	methionyl-tRNA synthetase	
CIMG_07183	1.55	0.00077	1.96	0.00108	conserved hypothetical protein	
CIMG_07194	2.65	2.26E-09	3.01	1.75E-08	conserved hypothetical protein (putative chromosome segregation protein Spc25)	
CIMG_07196	3.60	7.99E-25	3.07	1.52E-08	septin 3	
CIMG_07197	2.80	4.99E-17	1.88	0.00022	guanylate kinase	
CIMG_07209	7.45	1.18E-61	21.62	7.77E-59	conserved hypothetical protein	
CIMG_07215	1.45	0.00772	1.80	0.00060	exportin KapK	
CIMG_07221	1.69	0.00008	1.89	0.00125	DRE2 (Anamorsin family)	
CIMG_07222	1.78	7.01E-06	1.68	0.00029	conserved hypothetical protein (Nup133-like)	
CIMG_07226	2.03	8.30E-08	1.52	0.00392	imidazole glycerol phosphate synthase hisHF	
CIMG_07257	1.72	0.00003	1.77	0.00010	histone-lysine N-methyltransferase	
CIMG_07258	1.45	0.02001	3.07	5.57E-07	glucosamine-fructose-6-phosphate aminotransferase	
CIMG_07261	2.74	8.27E-08	8.10	7.82E-26	conserved hypothetical protein	
CIMG_07266	1.63	0.00010	2.80	6.65E-10	Putative ral1 binding protein 1	
CIMG_07268	1.60	0.00039	1.52	0.03943	pre-rRNA-processing protein PNO1	
CIMG_07271	1.41	0.00890	1.53	0.03156	proteasome component Pre4	
CIMG_07277	1.65	0.00024	4.02	5.28E-19	extracellular triacylglycerol lipase	*
CIMG_07285	5.74	1.33E-37	8.19	2.75E-28	conserved hypothetical protein	
CIMG_07289	1.65	0.00037	4.93	4.69E-09	major facilitator superfamily transporter	
CIMG_07290	5.58	2.78E-40	6.51	1.56E-17	eukaryotic phosphomannomutase	
CIMG_07298	3.15	1.05E-21	2.81	5.31E-09	peptide synthetase	
CIMG_07299	43.69	5.61E-137	1.48	0.03559	conserved hypothetical protein (putative FAD dependent oxidoreductase)	
CIMG_07303	22.35	2.01E-120	25.51	5.27E-60	prp 4 (CRoW domain-containing protein)	*
CIMG_07313	7.32	1.27E-30	15.79	2.32E-50	conserved hypothetical protein (type IV conjugative transfer system protein TraL)	
CIMG_07314	2.75	2.99E-10	7.23	4.27E-24	tyrosinase	*
CIMG_07326	2.45	7.47E-13	2.83	7.07E-09	peptidase (peptidase family M20/M25/M40)	*
CIMG_07327	1.56	0.00116	1.47	0.04327	translocon-associated protein	*
CIMG_07346	3.63	7.76E-20	2.15	0.00001	conserved hypothetical protein	
CIMG_07348	1.95	2.31E-06	6.46	5.50E-32	chitinase 4	*
CIMG_07460	1.45	0.00714	2.27	3.74E-08	kinesin family protein	
CIMG_07461	1.56	0.01269	1.40	0.00849	conserved hypothetical protein	
CIMG_07494	3.13	2.64E-20	1.94	0.00003	condensin complex component cnd2	
CIMG_07495	1.49	0.00182	2.92	3.74E-09	conserved hypothetical protein	
CIMG_07502	2.99	7.83E-19	2.19	4.00E-06	conserved hypothetical protein	*
CIMG_07520	2.21	1.57E-09	3.30	8.27E-13	serine/threonine-protein kinase chk2	
CIMG_07523	2.32	2.26E-06	1.97	0.00060	cell division control protein Cdc6	
CIMG_07524	1.85	3.06E-06	4.48	1.93E-19	Putative rho-GTPase-activating protein 8	
CIMG_07527	2.45	7.89E-09	1.79	0.00270	peroxin 10	
CIMG_07531	1.39	0.01700	1.96	0.00002	cyclin-dependent protein kinase complex component	+
CIMG_07552	8.59	5.36E-65	1.73	0.00391	phenylacetyl-CoA ligase	
CIMG_07554	7.52	7.43E-48	5.45	1.11E-22	conserved hypothetical protein	
CIMG_07556	256.26	6.94E-230	75.50	6.37E-84	conserved hypothetical protein (Acetyltransferase (GNAT) family)	
CIMG_07557	35.37	1.06E-148	22.83	1.39E-51	conserved hypothetical protein (Pyridine nucleotide-disulphide oxidoreductase)	
CIMG_07558	3.35	3.69E-24	3.67	1.30E-14	beta-N-acetylglucosaminidase	
CIMG_07569	4.01	2.19E-14	1.72	0.00508	allantoate permease	
CIMG_07577	1.79	1.27E-06	5.48	9.28E-21	conserved hypothetical protein (methyltransferase domain)	
CIMG_07578	2.34	6.52E-07	10.98	1.08E-35	conserved hypothetical protein	
CIMG_07580	4.27	4.82E-23	5.64	5.15E-22	conserved hypothetical protein (DUF202 superfamily)	
CIMG_07581	2.64	1.06E-13	2.40	6.53E-06	zinc homeostasis factor 1	*
CIMG_07582	1.48	0.00322	1.76	0.00062	manganese ion homeostasis	
CIMG_07588	5.75	2.24E-40	4.07	1.04E-15	checkpoint protein kinase	
CIMG_07599	1.41	0.01953	1.63	0.01013	MRS7 family protein	
CIMG_07616	1.95	2.66E-07	1.79	0.00014	4-nitrophenylphosphatase	
CIMG_07618	2.10	1.31E-09	1.63	0.00736	ATP-dependent Clp protease proteolytic subunit	
CIMG_07622	5.90	1.92E-16	2.55	0.00076	conserved hypothetical protein	
CIMG_07624	1.97	0.00002	1.78	0.00156	protein transport membrane glycoprotein Sec20	
CIMG_07634	2.15	9.00E-07	2.14	0.00069	conserved hypothetical protein (VMA21-like domain)	
CIMG_07649	1.38	0.02425	2.14	2.08E-07	tubulin-specific chaperone c	
CIMG_07653	9.15	4.04E-69	93.26	7.07E-73	conserved hypothetical protein (RNA recognition motif)	
CIMG_07658	5.72	7.64E-34	2.83	7.60E-08	MFS drug transporter	

*: Predicted secreted;
+: Gene under positive selection [19]; G: Gene gained in Coccidioides [19]
H, S: Up-regulated in Hyphae or Spherule by microarray [18]

Table S1: All genes up-regulated in saprobic phase

CIMG_07667	3.46	1.80E-21	4.71	1.22E-19	morphogenesis protein	
CIMG_07686	1.31	0.04105	1.41	0.03416	proteasome regulatory particle subunit	
CIMG_07689	2.34	1.42E-08	1.66	0.00030	translation repressor/antiviral protein Ski3	
CIMG_07690	4.03	6.79E-29	2.98	2.96E-12	glycosyl hydrolase	
CIMG_07697	1.48	0.00905	1.58	0.00276	transcriptional regulator	
CIMG_07708	2.00	2.12E-06	3.49	4.15E-08	Heterokaryon incompatibility protein Het-C	*
CIMG_07732	1.81	0.00006	4.07	9.10E-13	conserved hypothetical protein	
CIMG_07738	9.27	2.07E-71	21.32	7.58E-42	conserved hypothetical protein	* +
CIMG_07739	1.57	0.02138	2.54	5.01E-10	multicopper oxidase	
CIMG_07761	1.88	8.62E-07	1.58	0.01391	UDP-N-acetylglucosamine pyrophosphorylase	
CIMG_07775	3.44	3.01E-16	7.17	6.46E-35	conserved hypothetical protein (Glycerophosphoryl diester phosphodiesterase family)	
CIMG_07808	1.62	0.00033	1.49	0.03050	nuclear pore complex subunit Nup85	
CIMG_07809	3.04	1.26E-07	4.04	4.24E-09	conserved hypothetical protein	
CIMG_07811	17.27	1.53E-106	6.21	1.98E-14	ESDC	
CIMG_07819	2.23	2.06E-09	1.77	0.02190	fatty acid activator Faa4	
CIMG_07821	3.61	3.92E-12	7.11	2.17E-23	conserved hypothetical protein (NDT80 / PhoG like DNA-binding family)	
CIMG_07827	2.13	2.90E-09	1.77	0.00097	arf GTPase-activating protein	
CIMG_07839	14.42	7.29E-93	53.92	4.29E-58	conserved hypothetical protein	*
CIMG_07855	3.65	7.92E-24	11.68	1.88E-25	siderochrome-iron transporter Sit1	
CIMG_07859	2.09	2.03E-08	1.82	0.00013	Conserved hypothetical protein (DUF833)	
CIMG_07863	1.77	0.00001	2.90	1.52E-07	conserved hypothetical protein (acyltransferase family)	
CIMG_07868	1.56	0.00051	2.34	9.22E-07	NADPH oxidase regulator NoxR	
CIMG_07883	1.66	0.00010	1.95	2.44E-06	DNA repair and recombination protein RAD26	
CIMG_07889	3.43	1.31E-23	2.31	4.96E-07	DNA polymerase alpha catalytic subunit	
CIMG_07897	2.52	8.32E-13	1.84	0.00088	C2H2 zinc finger transcription factor	
CIMG_07907	1.96	3.50E-07	4.74	5.21E-16	mannitol-1-phosphate dehydrogenase	+
CIMG_07914	1.63	0.00034	1.72	0.00168	SAGA complex subunit	
CIMG_07925	2.87	2.69E-08	8.41	1.08E-21	conserved hypothetical protein	
CIMG_07934	1.42	0.00824	2.25	9.63E-08	U-box domain-containing protein	
CIMG_07935	1.79	0.00008	3.34	1.72E-08	conserved hypothetical protein (Acyl CoA binding protein)	
CIMG_07940	8.22	6.79E-63	3.84	1.83E-13	pH response protein PalF	
CIMG_07942	1.97	4.87E-08	2.21	0.00933	conserved hypothetical protein	
CIMG_07950	2.30	7.78E-10	1.95	0.00187	methyltransferase	
CIMG_07963	1.36	0.02214	2.38	1.45E-06	GPI mannosyltransferase 3	
CIMG_07967	2.82	5.00E-07	3.77	5.95E-13	conserved hypothetical protein	
CIMG_07980	1.96	3.90E-08	1.93	0.00007	nuclear condensin complex subunit Smc2	
CIMG_07984	1.31	0.03991	3.51	7.58E-12	conserved hypothetical protein (Tetratricopeptide repeat)	
CIMG_07993	1.88	5.50E-06	1.94	0.00009	actin binding protein	
CIMG_07994	1.69	0.00012	2.39	3.44E-07	conserved hypothetical protein	
CIMG_08011	2.27	3.36E-08	8.79	4.02E-31	conserved hypothetical protein	
CIMG_08012	1.86	0.00001	1.90	0.00016	protein kinase	
CIMG_08030	10.98	2.71E-57	11.80	9.80E-45	amine oxidase	*
CIMG_08031	1.76	0.00011	1.91	0.00101	conserved hypothetical protein	
CIMG_08039	4.73	1.07E-34	5.80	5.69E-18	integral membrane protein (SUR7 protein)	*
CIMG_08043	1.58	0.00082	1.69	0.00032	DNA excision repair protein Rad2	
CIMG_08045	2.18	5.73E-10	1.95	0.00017	chromosome segregation protein Spc105	
CIMG_08062	1.93	1.94E-06	3.72	1.99E-11	png1	
CIMG_08067	4.76	3.10E-37	2.77	1.01E-09	G1/S-specific cyclin Cln1	
CIMG_08071	3.21	6.86E-12	3.07	2.37E-08	TAM domain methyltransferase	
CIMG_08075	2.67	1.08E-15	1.82	0.00309	FK506-binding protein	*
CIMG_08076	2.85	7.48E-13	2.12	2.90E-06	conserved hypothetical protein (Cytochrome b5-like Heme/Steroid binding domain)	
CIMG_08090	2.04	1.10E-07	1.57	0.01701	SSOII	
CIMG_08092	2.28	3.07E-10	1.74	0.00244	eukaryotic translation initiation factor 4E-1	
CIMG_08102	1.50	0.00179	1.54	0.00544	isoleucyl-tRNA synthetase	
CIMG_08109	4.17	1.07E-14	1.85	0.00007	GPI mannosyltransferase 1	*
CIMG_08113	2.76	2.29E-12	2.81	5.52E-08	conserved hypothetical protein	
CIMG_08115	2.58	4.98E-14	2.17	0.00015	squalene synthetase	
CIMG_08120	1.45	0.02212	1.38	0.04837	DNA damage response protein RcaA	
CIMG_08129	5.23	1.13E-39	1.82	0.00346	conserved hypothetical protein	
CIMG_08160	1.89	3.54E-06	2.96	1.22E-08	metallopeptidase MepB	
CIMG_08162	3.31	2.38E-21	3.10	3.46E-09	calmodulin-beta	
CIMG_08164	2.56	7.35E-07	2.27	0.00021	conserved hypothetical protein	
CIMG_08173	2.21	2.14E-08	2.08	3.50E-06	conserved hypothetical protein (Arv1-like family)	
CIMG_08176	3.82	1.70E-15	2.20	0.00031	conserved hypothetical protein	
CIMG_08179	1.87	0.00006	2.20	0.00114	conserved hypothetical protein	
CIMG_08182	1.43	0.00701	1.81	0.00133	proteasome component PUP2	
CIMG_08190	1.49	0.01855	6.34	1.03E-16	galactose-1-phosphate uridylyltransferase	
CIMG_08192	2.22	2.62E-09	2.62	3.20E-07	conserved hypothetical protein (fungal Zn(2)-Cys(6) binuclear cluster domain)	

*: Predicted secreted;
+: Gene under positive selection [19]; G: Gene gained in Coccidioides [19]
H, S: Up-regulated in Hyphae or Spherule by microarray [18]

<u>Table S1</u>: All genes up-regulated in saprobic phase

CIMG_08198	1.51	0.00222	2.56	1.75E-06	ML domain-containing protein	*
CIMG_08199	2.03	4.99E-08	2.95	3.17E-10	vacuolar ATP synthase subunit F	
CIMG_08207	2.59	5.44E-10	2.50	8.62E-06	nuclear pore protein	
CIMG_08215	1.33	0.04319	2.83	5.96E-06	conserved hypothetical protein (C2H2 type zinc finger)	
CIMG_08216	1.56	0.00120	1.96	0.00006	ATP binding L-PSP endoribonuclease	
CIMG_08220	1.36	0.02247	2.54	9.56E-08	serine/threonine protein kinase	
CIMG_08288	1.33	0.04315	1.96	0.00115	aminopeptidase	
CIMG_08301	3.77	1.35E-19	2.64	9.31E-08	conserved hypothetical protein	
CIMG_08309	3.01	3.36E-19	1.67	0.00460	DUF757 domain-containing protein	
CIMG_08312	2.07	0.00001	5.27	1.81E-14	ABC a-pheromone efflux pump AtrD	
CIMG_08326	3.88	1.07E-23	4.55	2.88E-18	conserved hypothetical protein	
CIMG_08338	1.39	0.02746	1.89	0.00067	conserved hypothetical protein	G *
CIMG_08341	9.96	3.29E-49	13.05	4.70E-45	conserved hypothetical protein (Basic region leucine zipper)	
CIMG_08444	1.42	0.02201	2.26	0.00010	sterol 24-C-methyltransferase	
CIMG_08452	1.44	0.00957	2.03	0.00064	glycine dehydrogenase	
CIMG_08453	2.43	2.85E-12	3.62	1.53E-14	aromatic-L-amino acid decarboxylase	
CIMG_08455	1.51	0.01090	3.91	3.91E-13	bZIP transcription factor HacA	+
CIMG_08465	1.36	0.03194	2.89	8.84E-15	acid trehalase	*
CIMG_08473	5.79	1.06E-11	4.12	8.66E-06	DUF1881 domain-containing protein	
CIMG_08485	1.84	0.00211	10.05	1.52E-38	UMTA (methyltransferase)	
CIMG_08488	1.92	1.32E-06	2.17	3.54E-07	lipase/serine esterase	
CIMG_08497	2.31	8.09E-10	11.89	1.30E-31	conserved hypothetical protein	
CIMG_08498	6.61	1.63E-47	11.41	8.41E-41	conserved hypothetical protein (putative heterokaryon incompatibility protein – HET)	
CIMG_08499	14.68	5.01E-10	12.04	8.75E-11	hypothetical protein	
CIMG_08500	3.15	4.66E-12	1.83	0.00154	conserved hypothetical protein	
CIMG_08510	1.61	0.00017	2.40	3.42E-08	AAA family ATPase	
CIMG_08517	6.04	1.25E-49	2.42	8.00E-06	conserved hypothetical protein	
CIMG_08519	1.54	0.00087	1.81	0.00041	nuclear pore protein (Nup93/Nic96)	
CIMG_08522	4.26	6.76E-28	2.58	3.76E-06	protein tyrosine phosphatase	+
CIMG_08525	1.63	0.00027	1.37	0.02331	conserved hypothetical protein	*
CIMG_08528	1.95	8.27E-08	2.27	4.19E-08	calcium permease family membrane transporter	
CIMG_08532	2.07	1.42E-06	1.80	0.00257	ubiquitin fusion degradation protein	
CIMG_08541	2.60	6.98E-08	1.82	0.00030	conserved hypothetical protein (putative FAD dependent oxidoreductase)	
CIMG_08542	3.48	2.38E-12	5.12	4.54E-14	MFS multidrug transporter	
CIMG_08552	5.91	7.50E-21	1.96	0.00138	conserved hypothetical protein	
CIMG_08622	1.71	0.00030	1.87	0.00004	conserved hypothetical protein	
CIMG_08629	2.96	2.99E-19	5.50	1.21E-20	conserved hypothetical protein (phenazine biosynthesis protein PhzF family)	
CIMG_08630	11.20	6.82E-48	5.62	9.93E-21	conserved hypothetical protein	
CIMG_08646	3.14	2.17E-15	2.52	1.47E-06	thymidylate synthase	
CIMG_08649	1.68	0.00004	3.30	9.79E-14	histone deacetylase HosB	
CIMG_08650	1.68	0.00587	12.91	1.15E-35	leucine aminopeptidase 2	*
CIMG_08655	1.68	0.00008	5.69	2.79E-22	chitin synthase 2	
CIMG_08656	2.18	1.55E-10	3.31	6.20E-14	mitochondrial ATPase	
CIMG_08657	1.82	1.96E-06	2.64	5.08E-08	protein kinase activator Bem1	
CIMG_08659	1.64	0.00038	1.81	0.00041	conserved hypothetical protein	
CIMG_08665	9.90	7.39E-69	7.72	4.42E-21	conserved hypothetical protein	*
CIMG_08666	6.66	7.78E-52	6.51	1.61E-16	hypothetical protein (putative FKBP-type peptidyl-prolyl cis-trans isomerase)	
CIMG_08670	1.61	0.00029	1.67	0.00509	conserved hypothetical protein (hosphotransferase enzyme family)	
CIMG_08697	1.51	0.01300	1.41	0.01418	DEAD/DEAH box helicase	
CIMG_08714	2.35	1.19E-11	1.71	0.00071	COP9 signalosome subunit CsnD	
CIMG_08725	2.76	2.06E-16	1.43	0.04261	conserved hypothetical protein (alpha/beta hydrolase fold)	
CIMG_08761	1.62	0.01600	4.46	2.95E-16	conserved hypothetical protein (hosphotransferase enzyme family)	
CIMG_08762	1.64	0.00034	3.06	1.57E-07	conserved hypothetical protein	
CIMG_08769	1.88	1.55E-06	1.88	0.00063	chitin synthase activator	+
CIMG_08817	1.57	0.00537	1.73	0.00625	GNAT family acetyltransferase Nat4	
CIMG_08818	1.53	0.02094	1.54	0.01184	GPI transamidase component PIG-U	G *
CIMG_08827	2.35	0.00042	2.95	3.44E-06	conserved hypothetical protein	
CIMG_08829	1.66	0.00016	1.46	0.01327	glutaminyl cyclase	*
CIMG_08833	13.45	1.32E-95	12.88	8.36E-37	ABC bile acid transporter	
CIMG_08842	2.47	0.00184	4.64	3.57E-11	conserved hypothetical protein	
CIMG_08845	1.76	0.01858	18.28	1.45E-48	conserved hypothetical protein	
CIMG_08860	1.68	0.00005	1.81	0.00059	regulatory protein Ral2	
CIMG_08862	1.53	0.00396	2.33	4.92E-07	aldo/keto reductase	
CIMG_08863	1.40	0.03323	2.35	5.27E-08	conserved hypothetical protein (WD domain)	
CIMG_08881	2.37	1.75E-08	1.35	0.01921	RNA polymerase II mediator complex component SRB4	
CIMG_08892	1.59	0.00071	1.61	0.01042	bZIP transcription factor	+
CIMG_08916	2.23	7.19E-10	1.63	0.00233	conserved hypothetical protein	
CIMG_08922	140.97	2.90E-210	1.33	0.01456	GABA permease	
CIMG_08970	3.45	2.06E-20	2.37	5.14E-06	S-adenosylmethionine synthetase	

*: Predicted secreted;
+: Gene under positive selection [19]; G: Gene gained in Coccidioides [19]
H, S: Up-regulated in Hyphae or Spherule by microarray [18]

Table S1: **All genes up-regulated in saprobic phase**

CIMG_08972	3.19	1.17E-18	2.53	4.87E-07	conserved hypothetical protein	
CIMG_08973	3.52	3.98E-25	1.92	0.00071	FRE family ferric-chelate reductase	*
CIMG_08975	1.38	0.02496	3.41	1.51E-11	conserved hypothetical protein	
CIMG_08981	3.83	5.77E-16	2.36	0.00002	origin recognition complex subunit Orc1	
CIMG_08996	3.46	5.35E-24	1.90	0.00076	conserved hypothetical protein	
CIMG_08998	20.51	1.55E-74	1.68	0.03269	conserved hypothetical protein	
CIMG_09006	4.55	6.32E-19	1.65	0.00432	translation initiation factor eIF4E	
CIMG_09007	2.20	2.90E-09	1.51	0.00845	conserved hypothetical protein	
CIMG_09010	1.50	0.00460	2.54	4.61E-07	cation efflux family protein	
CIMG_09011	1.40	0.01408	1.93	0.00007	RING finger ubiquitin ligase	*
CIMG_09023	2.37	1.21E-11	2.90	4.30E-07	nuclear and cytoplasmic polyadenylated RNA-binding protein pub1	
CIMG_09033	3.64	2.64E-25	4.89	1.09E-22	acetyltransferase	**H**
CIMG_09034	1.86	0.00008	3.86	2.10E-14	conserved hypothetical protein	
CIMG_09037	3.09	0.00157	2.85	0.00258	conserved hypothetical protein	
CIMG_09050	1.69	0.00703	3.92	0.00512	conserved hypothetical protein (CVNH domain)	*
CIMG_09055	1.93	4.23E-07	4.21	2.73E-17	PQ loop repeat protein	
CIMG_09063	5.53	1.20E-40	2.39	4.97E-07	conserved hypothetical protein (Phospholipase/Carboxylesterase)	
CIMG_09071	1.48	0.02413	9.66	7.39E-23	homeobox domain-containing protein	
CIMG_09088	1.54	0.02062	1.93	4.57E-07	CND11p	
CIMG_09089	1.91	0.00026	5.05	1.19E-22	geranylgeranyl pyrophosphate synthase	
CIMG_09113	2.10	5.76E-09	1.56	0.03328	Endoplasmic reticulum vessicle protein ERV25	*
CIMG_09118	1.88	0.00001	2.84	1.18E-11	conserved hypothetical protein	
CIMG_09123	2.18	4.34E-10	2.57	2.48E-07	MFS transporter	
CIMG_09141	2.20	3.27E-10	2.16	8.15E-06	Ser/Thr protein phosphatase	
CIMG_09142	1.96	8.27E-08	1.66	0.00217	rho-gdp dissociation inhibitor	
CIMG_09167	3.09	2.18E-14	2.80	1.15E-07	secretory pathway protein Ssp120	*
CIMG_09170	1.77	0.00002	2.28	1.98E-07	protein-vacuolar targeting protein Atg18	
CIMG_09183	4.01	1.01E-30	4.37	3.98E-17	dynein heavy chain	
CIMG_09185	2.19	3.96E-08	3.79	2.25E-16	acetyltransferase	
CIMG_09196	3.10	9.13E-16	2.11	2.89E-06	rho guanyl-nucleotide exchange factor	
CIMG_09200	1.71	0.00004	1.48	0.03894	pre-mRNA splicing factor syf-1	
CIMG_09215	3.49	4.34E-16	2.58	1.54E-08	ubiquitin C-terminal hydrolase	
CIMG_09222	1.95	5.25E-07	1.56	0.00499	integral membrane protein	
CIMG_09273	3.12	2.30E-18	1.99	0.00002	kinesin family protein	
CIMG_09291	3.07	3.00E-19	5.12	2.45E-17	cell wall glucanase	*
CIMG_09301	7.33	7.73E-58	2.37	4.37E-07	conserved hypothetical protein	
CIMG_09304	64.11	3.15E-150	21.11	1.58E-34	wall-associated proteinase	
CIMG_09306	3.35	5.51E-22	6.67	3.82E-32	conserved hypothetical protein	
CIMG_09318	4.32	2.88E-21	2.17	0.00001	DNA repair protein rad5	
CIMG_09326	2.48	3.08E-11	1.69	0.00046	decapping enzyme Dcp2	
CIMG_09328	3.22	4.53E-21	5.88	4.58E-28	conserved hypothetical protein	
CIMG_09348	2.08	0.00098	1.93	0.00002	conserved hypothetical protein	
CIMG_09360	1.45	0.02033	1.89	0.00017	conserved hypothetical protein	
CIMG_09364	3.45	6.64E-07	4.05	1.57E-08	MFS sugar transporter	*
CIMG_09377	2.25	7.59E-11	2.68	6.90E-08	C6 transcription factor	
CIMG_09378	2.34	3.38E-06	3.01	5.46E-09	conserved hypothetical protein (putative O-methyltransferase)	
CIMG_09395	2.58	8.12E-12	1.63	0.00485	conserved hypothetical protein	
CIMG_09396	2.03	5.16E-08	2.00	0.00004	C2H2 transcription factor	
CIMG_09401	2.07	9.22E-09	1.97	0.00026	Na(+)/H(+) antiporter	
CIMG_09402	3.36	1.99E-22	2.26	6.50E-06	integral membrane protein (Mpv17 / PMP22 family)	
CIMG_09425	2.53	5.74E-11	1.75	0.00047	CAF1 family ribonuclease	
CIMG_09427	7.57	1.48E-39	2.61	1.39E-07	peroxin 26	
CIMG_09438	1.32	0.03049	1.64	0.00147	bimD protein	
CIMG_09443	1.54	0.00092	1.86	0.00006	SWI-SNF complex subunit	
CIMG_09448	2.07	2.14E-07	1.89	0.00208	peroxisome biosynthesis protein	
CIMG_09452	1.64	0.00029	1.50	0.01193	Conserved hypothetical protein (DUF1237)	*
CIMG_09465	2.34	6.30E-06	2.53	3.13E-06	conserved hypothetical protein	
CIMG_09482	1.49	0.00456	1.81	0.01747	phospholipase D active site-containing protein	
CIMG_09488	2.88	8.83E-17	4.40	1.98E-19	glutathione S-transferase	
CIMG_09490	1.98	4.19E-07	2.19	6.40E-07	palmitoyltransferase pfa3	
CIMG_09512	2.93	8.05E-19	4.72	2.52E-17	uracil phosphoribosyltransferase	
CIMG_09513	3.01	2.21E-17	2.34	3.83E-06	C2H2 zinc finger domain-containing protein	
CIMG_09533	2.45	1.71E-12	2.47	5.32E-07	conserved hypothetical protein (putative sensor histidine kinase/response regulator)	
CIMG_09555	5.72	2.63E-47	5.07	2.26E-22	zinc metalloprotease	
CIMG_09560	13.94	2.49E-96	1.74	0.03896	proline rich antigen 2	*
CIMG_09564	4.75	1.55E-34	7.69	1.12E-29	conserved hypothetical protein	
CIMG_09565	4.28	3.39E-26	4.63	2.38E-13	conserved hypothetical protein	
CIMG_09566	3.24	3.53E-21	2.60	7.45E-06	ornithine decarboxylase	
CIMG_09567	5.84	1.13E-47	14.28	2.94E-40	conserved hypothetical protein	

*: Predicted secreted;

+: Gene under positive selection [19]; G: Gene gained in Coccidioides [19]

H, S: Up-regulated in Hyphae or Spherule by microarray [18]

Table S1: All genes up-regulated in saprobic phase

CIMG_09578	2.86	5.48E-13	7.88	1.42E-26	phospholipase D	*
CIMG_09589	2.08	3.38E-09	1.58	0.00073	cation chloride cotransporter	
CIMG_09617	3.08	1.83E-11	7.92	1.80E-18	RNA exonuclease Rex3	
CIMG_09624	1.61	0.00113	2.59	5.96E-08	conserved hypothetical protein (putative ATPase)	
CIMG_09625	2.82	4.22E-14	3.22	6.17E-11	conserved hypothetical protein (Transcription factor Pcc1)	
CIMG_09628	5.19	1.40E-39	4.01	1.35E-17	protein-tyrosine phosphatase	
CIMG_09634	2.10	3.61E-06	2.27	0.00014	homeobox transcription factor	
CIMG_09636	1.86	2.97E-06	3.08	1.53E-10	conserved hypothetical protein	
CIMG_09663	1.61	0.00037	2.03	0.00006	serine/threonine protein phosphatase PP2A catalytic subunit	
CIMG_09664	4.49	2.41E-33	5.52	1.63E-17	conserved hypothetical protein	
CIMG_09666	1.67	0.00007	1.60	0.00708	HAD superfamily hydrolase	
CIMG_09676	3.23	1.45E-17	1.65	0.01188	conserved hypothetical protein (twin-arginine translocation (Tat) pathway signal sequence)	
CIMG_09701	1.90	2.97E-06	3.21	8.54E-13	DNA polymerase delta subunit 4	
CIMG_09713	1.57	0.00122	1.61	0.00930	zinc finger protein ZPR1	
CIMG_09740	1.49	0.01695	17.07	3.28E-51	conserved hypothetical protein	
CIMG_09756	1.31	0.04137	1.48	0.03540	conserved hypothetical protein	
CIMG_09759	1.91	1.41E-06	3.12	9.12E-12	conserved hypothetical protein	
CIMG_09760	1.45	0.01343	1.92	0.00141	DUF1275 domain-containing protein	
CIMG_09762	3.39	2.69E-13	1.97	0.00041	acyl-CoA dehydrogenase	
CIMG_09773	2.97	8.21E-09	9.41	8.50E-19	fungal chitosanase	*
CIMG_09781	8.56	3.29E-68	15.25	3.89E-45	hypothetical protein	
CIMG_09786	2.20	2.49E-09	2.39	5.93E-07	RNA binding domain-containing protein	
CIMG_09790	1.56	0.00080	1.54	0.00690	conserved hypothetical protein (Zinc finger)	
CIMG_09802	1.44	0.00633	1.99	0.00001	F-box and WD domain-containing protein	
CIMG_09805	20.47	2.65E-61	9.04	1.93E-17	aldehyde dehydrogenase	
CIMG_09811	2.62	2.23E-13	3.14	8.86E-12	proline utilization protein PrnX	
CIMG_09812	2.42	0.00008	8.65	3.45E-15	conserved hypothetical protein	
CIMG_09817	5.22	5.51E-31	1.88	0.02434	high affinity copper transporter	
CIMG_09823	3.66	8.11E-14	6.16	6.83E-23	benzoate 4-monooxygenase cytochrome P450	*
CIMG_09824	52.37	1.70E-163	97.31	6.33E-67	conserved hypothetical protein (PAN domain)	*
CIMG_09826	10.80	4.92E-63	11.80	9.26E-38	conserved hypothetical protein	
CIMG_09828	3.71	1.19E-26	8.07	2.82E-31	conserved hypothetical protein (PAN domain)	G *
CIMG_09911	4.92	1.05E-39	5.54	3.32E-17	amino-acid permease inda1	
CIMG_09916	1.50	0.00274	1.23	0.03348	protein kinase and ribonuclease Ire1	*
CIMG_09961	1.50	0.00172	3.69	3.09E-12	C6 finger domain-containing protein	
CIMG_09962	2.66	8.89E-15	3.10	5.98E-12	VelB	
CIMG_09977	1.43	0.01277	1.99	0.00030	conserved hypothetical protein	
CIMG_09984	2.79	7.76E-17	2.31	0.00003	glycerol-3-phosphate dehydrogenase	
CIMG_09985	1.63	0.00020	1.62	0.01754	riboflavin aldehyde-forming enzyme	
CIMG_10016	2.50	1.58E-12	2.35	3.08E-07	dynamin GTPase	
CIMG_10029	1.87	9.58E-07	2.60	1.03E-07	glucose N-acetyltransferase 1	*
CIMG_10038	3.17	5.11E-21	1.80	0.00333	metalloreductase	
CIMG_10049	4.04	7.43E-23	1.87	0.00342	C2H2 finger domain-containing protein	
CIMG_10051	2.86	1.47E-11	2.82	1.48E-07	G2-specific protein kinase nimA	
CIMG_10073	1.56	0.00065	1.73	0.00300	conserved hypothetical protein	
CIMG_10075	2.81	1.57E-12	2.03	5.81E-06	glutamine synthetase	
CIMG_10096	1.94	0.00014	2.21	0.00004	choline transporter	
CIMG_10105	1.61	0.00715	7.17	3.04E-19	glycerol kinase	*
CIMG_10129	5.04	1.16E-27	2.08	0.00002	oligopeptide transporter	
CIMG_10157	3.21	1.95E-06	18.61	1.13E-58	conserved hypothetical protein (RNA exonuclease 3)	
CIMG_10165	1.77	0.00046	2.62	6.52E-06	aflatoxin efflux pump protein	
CIMG_10171	1.42	0.00780	1.37	0.04825	phospholipid-translocating P-type ATPase	S
CIMG_10179	1.54	0.00190	1.93	0.00023	malate/L-lactate dehydrogenase	
CIMG_10191	7.69	3.48E-57	8.78	2.65E-42	extracellular elastinolytic metalloproteinase	*
CIMG_10207	1.35	0.04611	1.63	0.00502	cell division cycle protein 123	
CIMG_10212	2.33	6.52E-08	2.22	4.41E-07	hypothetical protein (RF-1 domain)	
CIMG_10213	1.79	5.15E-06	2.17	0.00003	chromosome segregation protein Cse1	
CIMG_10217	11.92	1.45E-82	24.19	3.14E-64	conserved hypothetical protein	
CIMG_10229	1.93	0.03640	3.28	0.00001	conserved hypothetical protein	
CIMG_10251	3.06	2.58E-19	1.71	0.00283	mitochondrial 2-oxodicarboxylate carrier protein	
CIMG_10254	1.62	0.00060	1.29	0.04068	HLH transcription factor	
CIMG_10258	2.13	4.74E-08	2.83	8.72E-11	putative golgi traffic protein SFT2	
CIMG_10262	4.74	1.34E-23	7.92	4.03E-26	PAP2 domain-containing protein	
CIMG_10263	3.62	2.42E-09	3.21	1.84E-06	conserved hypothetical protein (AIG2-like family)	
CIMG_10292	2.17	2.23E-09	2.20	0.00212	cation transport ATPase	
CIMG_10298	1.82	9.74E-06	4.09	1.58E-12	conserved hypothetical protein (YD repeat motif)	
CIMG_10308	2.32	9.66E-12	2.09	0.00010	conserved hypothetical protein	
CIMG_10310	1.73	0.00004	1.51	0.00336	pre-mRNA splicing factor	
CIMG_10311	2.58	3.90E-10	2.98	2.60E-06	adenosylhomocysteinase	

*: Predicted secreted;
+: Gene under positive selection [19]; G: Gene gained in Coccidioides [19]
H, S: Up-regulated in Hyphae or Spherule by microarray [18]

Table S1: All genes up-regulated in saprobic phase

CIMG_10314	1.82	1.84E-06	2.13	2.42E-06	ATP-binding cassette sub-family E member 1	
CIMG_10316	2.28	2.13E-07	1.51	0.00929	conserved hypothetical protein	
CIMG_10332	1.84	2.25E-06	2.30	3.07E-08	conserved hypothetical protein	
CIMG_10468	4.98	1.43E-07	50.17	5.70E-18	hypothetical protein	
CIMG_10473	22.19	1.86E-50	8.13	2.23E-19	GNAT family N-acetyltransferase	
CIMG_10563	1.44	0.02396	3.46	1.12E-13	intracellular protease/amidase	
CIMG_10564	4.88	1.27E-15	4.79	8.00E-23	conserved hypothetical protein	
CIMG_10567	7.25	2.68E-56	35.66	1.51E-42	conserved hypothetical protein	*
CIMG_10574	15.58	5.15E-33	2.82	4.68E-07	conserved hypothetical protein	
CIMG_10658	1.33	0.03600	1.31	0.01603	conserved hypothetical protein	
CIMG_10667	1.32	0.04675	1.49	0.01916	SUA5	
CIMG_10671	2.26	1.23E-08	3.48	3.13E-15	conserved hypothetical protein (putative ER protein Pkr1)	*
CIMG_10681	2.61	6.99E-06	7.51	7.67E-20	hypothetical protein	
CIMG_10707	1.89	7.88E-06	2.47	0.00004	conserved hypothetical protein	
CIMG_10708	1.54	0.00122	1.38	0.04734	hypothetical protein (Smr domain, DUF1771, CUE domain)	
CIMG_10765	2.09	5.81E-09	2.03	0.00001	Sit4-associated protein	
CIMG_10772	1.79	0.00046	3.51	1.55E-11	hypothetical protein	*
CIMG_10814	2.97	4.72E-10	1.98	0.00098	hypothetical protein	
CIMG_10817	11.59	1.19E-07	2.45	0.03195	hypothetical protein	
CIMG_10821	4.91	1.80E-15	2.83	1.04E-06	conserved hypothetical protein	
CIMG_10851	12.60	1.31E-84	84.79	4.69E-73	conserved hypothetical protein	*
CIMG_10854	6.28	2.97E-08	4.98	3.53E-09	amidase	
CIMG_10937	4.65	4.02E-10	18.60	1.34E-15	conserved hypothetical protein	
CIMG_10951	1.71	0.00040	2.68	2.68E-09	nucleoside transporter	
CIMG_10952	2.04	0.00012	2.65	0.00001	conserved hypothetical protein (metallo-beta-lactamase superfamily)	
CIMG_11124	2.51	1.23E-09	2.58	9.31E-07	kinesin family protein	
CIMG_11148	2.62	9.11E-14	3.35	2.48E-12	TATA-box binding protein	
CIMG_11158	5.40	1.50E-44	2.30	1.01E-06	hypothetical protein (putative translation machinery-associated TMA7)	
CIMG_11171	1.90	0.00001	1.84	0.00076	conserved hypothetical protein (MAGE family)	
CIMG_11248	4.08	2.94E-31	2.19	0.00601	conserved hypothetical protein	
CIMG_11269	2.71	0.00030	3.47	0.00009	diphosphomevalonate decarboxylase	
CIMG_11271	9.38	3.53E-27	12.11	1.09E-21	hypothetical protein	
CIMG_11316	7.54	1.97E-26	4.89	7.21E-13	secretory phospholipase A2	* H
CIMG_11329	1.65	0.00011	2.87	7.89E-12	conserved hypothetical protein (putative FAD dependent oxidoreductase)	*
CIMG_11333	1.40	0.01396	3.37	1.05E-09	hypothetical protein	
CIMG_11365	2.63	1.66E-10	2.47	4.14E-06	MFS nicotinic acid transporter Tna1	
CIMG_11378	24.96	3.75E-133	20.72	2.25E-51	conserved hypothetical protein	
CIMG_11421	5.25	2.16E-40	3.22	1.89E-12	Gpg1	
CIMG_11540	7.55	9.02E-39	7.18	1.12E-18	conserved hypothetical protein	
CIMG_11570	1.97	1.34E-06	1.57	0.00850	hypothetical protein (G-beta repeat, WD domain)	
CIMG_11575	6.36	4.30E-33	3.81	2.18E-14	phosphotyrosine protein phosphatase	
CIMG_11576	3.39	1.86E-18	2.89	6.34E-11	conserved hypothetical protein	
CIMG_11581	2.31	7.06E-06	2.00	0.04477	hypothetical protein (FAD binding domain)	
CIMG_11656	1.76	0.00003	1.72	0.00592	Spo12 family protein	
CIMG_11666	3.43	4.47E-19	3.23	3.77E-10	carboxypeptidase S1	*
CIMG_11667	1.70	0.00009	3.68	1.77E-10	polymerase	
CIMG_11693	3.35	6.57E-14	2.20	4.52E-06	conserved hypothetical protein	
CIMG_11720	2.63	1.34E-07	3.11	0.00311	hypothetical protein	
CIMG_11736	1.54	0.00180	1.95	0.00006	DNA repair protein Rad14	
CIMG_11744	19.42	5.69E-84	1.49	0.01929	conserved hypothetical protein (Enoyl-CoA hydratase/isomerase family)	
CIMG_11746	1.94	8.19E-06	1.74	0.00443	zinc finger protein	
CIMG_11749	2.20	8.71E-07	2.77	0.00005	serine/threonine-protein kinase 24	
CIMG_11772	2.46	1.12E-06	2.55	0.00001	hypothetical protein	
CIMG_11792	1.56	0.00046	1.78	0.00027	amino acid permease Dip5	
CIMG_11826	2.48	3.13E-06	5.65	2.48E-18	hypothetical protein	
CIMG_11829	3.54	1.13E-25	2.84	2.59E-11	myosin type II heavy chain	
CIMG_11835	3.91	9.77E-22	11.29	7.45E-35	ubiquitin C-terminal hydrolase L3	
CIMG_11881	2.35	2.26E-08	3.32	8.35E-09	cross-pathway control protein A (bZIP transcription factor)	
CIMG_11930	1.44	0.00434	2.21	3.14E-06	proteasome component	
CIMG_11934	13.68	3.86E-89	24.54	2.64E-60	conserved hypothetical protein (DMATS type aromatic prenyltransferase)	
CIMG_11945	12.94	2.41E-93	4.53	3.95E-06	hypothetical protein	
CIMG_12056	2.18	1.80E-07	1.69	0.00462	glucosyltransferase	*
CIMG_12070	2.15	5.19E-07	1.73	0.00031	aminotransferase	
CIMG_12080	1.73	0.00037	3.37	1.49E-09	hypothetical protein	
CIMG_12094	28.11	1.26E-133	2.18	7.88E-06	hypothetical protein	
CIMG_12139	1.82	2.55E-06	1.91	0.00002	mRNA splicing protein	
CIMG_12175	1.60	0.00030	1.59	0.01824	chromatin remodeling complex subunit (SWI/SNF and RSC complex subunit Arp9)	
CIMG_12238	2.06	0.00012	3.84	4.92E-10	conserved hypothetical protein	
CIMG_12261	12.23	3.99E-30	2.77	6.60E-07	conserved hypothetical protein	*

*: Predicted secreted;
+: Gene under positive selection [19]; G: Gene gained in Coccidioides [19]
H, S: Up-regulated in Hyphae or Spherule by microarray [18]

Table S1: All genes up-regulated in saprobic phase

CIMG_12262	4.48	1.26E-06	3.14	1.43E-08	hypothetical protein	*
CIMG_12293	1.66	0.00051	2.23	0.00002	conserved hypothetical protein (putative multi-copper polyphenol oxidoreductase laccase)	
CIMG_12306	3.90	3.61E-29	2.85	2.37E-09	conserved hypothetical protein	
CIMG_12323	1.80	7.92E-06	1.48	0.00781	nuclear pore complex subunit Nup192	
CIMG_12324	1.56	0.00061	1.40	0.00363	ataxia telangiectasia mutated	
CIMG_12338	1.56	0.00064	2.05	8.27E-06	mitochondrial DNA helicase	
CIMG_12351	2.17	2.56E-06	1.42	0.01289	class I alpha-mannosidase	*
CIMG_12402	7.88	4.89E-18	3.10	1.95E-06	conserved hypothetical protein	
CIMG_12416	2.11	0.00020	1.44	0.03943	conserved hypothetical protein (ERCC4 domain)	
CIMG_12417	2.54	1.85E-14	2.65	1.72E-08	kinesin family protein	
CIMG_12429	2.80	2.86E-15	14.88	2.99E-54	conserved hypothetical protein (Dynamin family)	
CIMG_12440	3.32	4.70E-23	3.67	2.96E-12	pyridoxamine phosphate oxidase	
CIMG_12504	1.42	0.01169	2.22	0.00002	hypothetical protein (DASH complex subunit Hsk3 like)	
CIMG_12678	1.92	1.01E-06	1.62	0.00486	RNase H domain-containing protein	
CIMG_12682	6.22	2.02E-12	5.46	1.13E-08	hypothetical protein	
CIMG_12754	1.68	0.00003	1.96	0.00013	hypothetical protein (kelch motif)	
CIMG_12755	5.26	1.42E-42	2.41	5.99E-07	conserved hypothetical protein	
CIMG_12768	6.94	7.87E-26	4.94	6.54E-13	hypothetical protein	
CIMG_12777	3.22	1.45E-09	8.20	4.97E-12	hypothetical protein	*
CIMG_12815	11.05	1.93E-44	12.29	1.11E-26	serine/threonine-protein kinase Eg2	
CIMG_12857	1.95	1.32E-07	1.88	0.00037	conserved hypothetical protein	
CIMG_12859	1.32	0.04569	1.50	0.00299	calcium-transporting ATPase 3	
CIMG_12875	1.78	6.39E-06	3.29	2.31E-13	urea transporter	
CIMG_12876	4.77	1.43E-35	2.60	0.00009	conserved hypothetical protein	
CIMG_13010	2.73	5.74E-15	4.19	6.93E-13	serine/threonine protein kinase	
CIMG_13024	1.37	0.01459	1.60	0.00629	conserved hypothetical protein (SAC3/GANP/Nin1/mts3/eIF-3 p25 family)	
CIMG_13039	1.50	0.00678	1.69	0.00160	conserved hypothetical protein	
CIMG_13043	2.12	8.57E-09	1.84	0.00019	conserved hypothetical protein	*
CIMG_13055	4.81	1.33E-18	17.22	5.53E-31	hypothetical protein	*
CIMG_13059	3.84	9.92E-27	11.82	1.92E-47	nitrogen metabolite repression regulator NmrA	
CIMG_13081	4.38	4.00E-06	3.37	0.00010	hypothetical protein	
CIMG_13089	3.69	4.32E-26	3.09	4.16E-07	hypothetical protein	
CIMG_13108	3.48	0.01145	2.72	0.00212	conserved hypothetical protein	
CIMG_13109	7.57	8.48E-36	2.41	0.00147	ThiJ/PfpI family protein	
CIMG_13117	2.48	0.00108	2.03	0.01041	hypothetical protein	
CIMG_13165	3.18	4.29E-12	7.37	6.20E-22	serine/threonine protein kinase	
CIMG_13166	2.14	0.00384	2.98	0.00061	conserved hypothetical protein	
CIMG_13167	6.81	6.01E-51	3.62	4.17E-10	beta-glucosidase 5	
CIMG_13253	1.50	0.02582	3.08	3.89E-08	hypothetical protein	
CIMG_13268	2.54	2.72E-14	2.66	5.62E-09	hypothetical protein (Kinesin motor domain)	
CIMG_13285	2.89	1.99E-06	8.48	4.52E-25	hypothetical protein	
CIMG_13368	2.01	1.95E-07	4.16	1.06E-14	hypothetical protein	
CIMG_13374	13.79	9.41E-82	49.09	1.11E-81	hypothetical protein	
CIMG_13400	3.26	6.42E-13	2.74	4.87E-07	calmodulin-binding protein Sha1	
CIMG_13415	2.74	5.03E-13	6.04	9.06E-13	hypothetical protein	
CIMG_13481	2.82	1.15E-15	2.21	3.40E-07	conserved hypothetical protein (Zinc finger)	
CIMG_13501	2.53	5.01E-13	5.04	2.37E-19	hypothetical protein	
CIMG_13545	9.99	2.39E-13	1.60	0.03389	conserved hypothetical protein	
CIMG_13549	2.96	0.01541	2.62	1.17E-06	hypothetical protein	
CIMG_13558	3.59	1.84E-17	2.40	3.57E-06	DNA replication helicase Dna2	
CIMG_13567	1.83	0.00090	2.65	6.34E-10	conserved hypothetical protein	*
CIMG_13583	1.58	0.00093	1.69	0.00153	endocytosis-associated NECAP 1	
CIMG_13585	1.35	0.03449	2.22	1.63E-06	kinetochore protein nuf2	
CIMG_13593	2.54	1.67E-13	2.03	0.00002	GPI-anchored cell surface glycoprotein	
CIMG_13670	2.55	1.04E-13	1.94	0.00004	dihydrouridine synthase	
CIMG_13686	4.72	5.46E-18	2.26	2.88E-07	hypothetical protein	
CIMG_13702	1.84	0.00145	4.08	1.32E-09	conserved hypothetical protein	
CIMG_13704	1.88	0.00968	2.20	0.00032	conserved hypothetical protein	
CIMG_13710	3.06	0.00092	2.75	0.00021	conserved hypothetical protein	
CIMG_13722	3.81	7.38E-24	3.91	1.79E-14	hypothetical protein (SH3 domain)	
CIMG_13742	1.91	0.00094	4.07	2.68E-09	hypothetical protein	
CIMG_13753	1.76	0.00017	8.02	8.02E-34	conserved hypothetical protein (major facillitator superfamily)	
CIMG_13762	1.64	0.00719	2.25	0.00017	hypothetical protein	

*: Predicted secreted;
+: Gene under positive selection [19]; G: Gene gained in Coccidioides [19]
H, S: Up-regulated in Hyphae or Spherule by microarray [18]
