# Supplementary material for: Comparative Transcriptomics of the Saprobic and Parasitic Growth Phases in Coccidioides spp
Source: PLoS One. 2012 Jul 20;7(7):e41034. doi: 10.1371/journal.pone.0041034 (PMC3401177; doi:10.1371/journal.pone.0041034)
Supplement: Table S2 — All genes with significantly higher expression (up-regulated) in the parasitic phase in both C. immitis and C. posadasii. *: Predicted secreted; +: Gene under positive selection [19]; G: Gene gained in Coccidioides [19]; H, S: Previously found to be up-regulated in saprobic-phase Hyphae or parasitic-phase Spherules by microarray [18]. (PDF) [file pone.0041034.s002.pdf]

<u>Table S2</u>: All genes up-regulated in parasitic phase

BROAD ID	*C. immitis* S/H	*C. immitis p*-value	*C. posadasii* S/H	*C. posadasii* p-value	Gene description	Previous studies
CIMG_00037	2.11	6.90E-07	1.80	0.00228	conserved hypothetical protein	
CIMG_00048	1.65	0.00160	1.81	0.00108	metaphase-anaphase transition protein	
CIMG_00063	1.62	0.00147	1.75	0.00283	serine/threonine-protein kinase ssn3	
CIMG_00073	3.40	1.72E-10	4.52	7.54E-10	conserved hypothetical protein	
CIMG_00082	3.89	4.56E-09	1.87	0.01104	conserved hypothetical protein	
CIMG_00086	6.00	1.90E-11	1.84	0.02498	conserved hypothetical protein	*
CIMG_00092	2.44	1.62E-08	3.09	7.77E-10	PH domain-containing protein	
CIMG_00094	2.08	0.00003	1.59	0.01096	acc synthase	
CIMG_00124	1.92	0.00006	1.37	0.00346	glutathione S-transferase	
CIMG_00131	2.20	0.00001	2.24	0.00229	gamma-cysteine synthetase regulatory subunit	
CIMG_00134	1.43	0.01486	2.62	2.38E-07	NADP-dependent alcohol dehydrogenase	
CIMG_00137	1.90	0.00005	1.99	0.00032	peroxin 24	
CIMG_00138	3.84	4.30E-12	3.92	3.77E-10	conserved hypothetical protein	G
CIMG_00148	3.86	2.49E-09	1.73	0.00029	conserved hypothetical protein	
CIMG_00151	3.02	8.11E-13	2.38	0.00002	dihydrodipicolinate synthetase	
CIMG_00159	5.87	2.90E-12	4.25	1.74E-06	conserved hypothetical protein	*
CIMG_00161	2.28	0.00001	4.23	2.58E-10	HLH transcription factor	+
CIMG_00165	2.59	0.01466	2.84	0.00333	conserved hypothetical protein	
CIMG_00168	2.29	0.00748	3.21	0.00004	conserved hypothetical protein	
CIMG_00180	1.64	0.00322	4.63	6.73E-11	conserved hypothetical protein	
CIMG_00186	1.76	0.00273	1.48	0.01909	conserved hypothetical protein	
CIMG_00190	1.79	0.00025	1.83	0.00007	glycerophosphoryl diester phosphodiesterase	
CIMG_00194	3.19	7.32E-07	5.60	1.82E-13	conserved hypothetical protein	
CIMG_00195	1.71	0.00354	2.91	9.72E-07	conserved hypothetical protein	*
CIMG_00205	2.14	3.05E-06	2.62	3.83E-06	putative DNA helicase	
CIMG_00206	2.04	4.70E-07	3.38	1.32E-13	sensor histidine kinase/response regulator Fos-1	S
CIMG_00230	3.08	1.66E-09	1.75	0.02449	conserved hypothetical protein (Fungal Zn(2)-Cys(6) binuclear cluster domain)	
CIMG_00242	1.53	0.00341	2.38	6.62E-06	TBC domain-containing protein	
CIMG_00252	1.44	0.01108	1.62	0.04927	sphingosine-1-phosphate phosphohydrolase	
CIMG_00259	2.61	9.39E-10	1.88	0.00017	DUF866 domain-containing protein	
CIMG_00270	1.58	0.00184	1.81	0.00069	conserved hypothetical protein	
CIMG_00273	1.51	0.00450	1.60	0.00427	DUF92 domain-containing protein	*
CIMG_00277	5.33	1.80E-12	4.19	1.41E-11	conserved hypothetical protein	
CIMG_00281	2.10	0.00004	20.82	1.26E-44	conserved hypothetical protein	
CIMG_00287	1.41	0.02256	1.57	0.01568	conserved hypothetical protein (Gamma interferon inducible lysosomal thiol reductase (GILT))	
CIMG_00294	2.81	6.69E-11	4.07	1.40E-14	calcium dependent mitochondrial carrier protein	
CIMG_00295	3.07	5.82E-13	5.24	2.19E-19	C-type cyclin	
CIMG_00296	2.52	3.66E-06	4.46	1.67E-08	conserved hypothetical protein	
CIMG_00299	2.69	1.98E-11	13.24	1.03E-36	conserved hypothetical protein	
CIMG_00308	2.43	4.58E-06	2.02	0.00382	sister chromatid cohesion protein Dcc1	
CIMG_00311	2.38	0.00587	8.62	8.72E-19	aspartyl protease 3	*
CIMG_00325	1.89	0.02557	3.04	0.01865	conserved hypothetical protein	
CIMG_00326	2.22	0.00528	1.69	0.03956	conserved hypothetical protein	
CIMG_00330	2.02	4.72E-06	1.61	0.02179	conserved hypothetical protein (Methyltransferase domain)	
CIMG_00336	2.55	0.00001	1.68	0.03396	conserved hypothetical protein	
CIMG_00341	5.06	5.88E-13	2.14	0.00133	DEAD/DEAH box RNA helicase	
CIMG_00342	1.89	0.00010	1.81	0.00390	glyoxal oxidase	
CIMG_00348	3.46	9.81E-12	6.55	1.79E-20	endochitinase 2	*
CIMG_00351	5.21	2.82E-15	3.42	2.22E-06	conserved hypothetical protein	*
CIMG_00356	1.86	0.00015	2.97	3.74E-09	conserved hypothetical protein (putative acetyltransferase)	
CIMG_00373	3.81	1.81E-06	2.79	0.00637	conserved hypothetical protein	
CIMG_00406	3.75	1.12E-11	1.41	0.00163	HMG domain-containing protein	
CIMG_00407	3.68	0.00076	3.14	8.11E-06	conserved hypothetical protein	
CIMG_00410	1.97	0.00001	2.01	0.00024	conserved hypothetical protein (putative integral membrane protein)	*
CIMG_00417	1.41	0.01744	1.76	0.00275	conserved hypothetical protein	
CIMG_00426	2.51	2.15E-07	2.27	0.00063	COX1 assembly protein	
CIMG_00431	1.58	0.00865	1.83	0.00134	2-dehydropantoate 2-reductase	
CIMG_00452	5.74	1.11E-06	4.32	2.72E-06	conserved hypothetical protein	
CIMG_00453	1.87	0.00003	1.54	0.02790	conserved hypothetical protein	
CIMG_00455	2.90	0.00195	3.55	0.00883	conserved hypothetical protein	
CIMG_00461	2.05	3.44E-06	2.60	9.86E-09	deoxyribose-phosphate aldolase 1	
CIMG_00466	4.07	1.38E-09	1.94	0.00278	conserved hypothetical protein	G
CIMG_00471	1.65	0.00041	4.16	8.81E-07	hypothetical protein	
CIMG_00477	1.89	0.00003	2.00	0.00008	conserved hypothetical protein	
CIMG_00478	5.26	3.07E-10	2.02	0.01839	conserved hypothetical protein	
CIMG_00490	1.69	0.02853	4.15	7.86E-08	conserved hypothetical protein (DUF1203)	
CIMG_00496	2.83	2.28E-11	3.20	6.59E-11	sister chromatid cohesion acetyltransferase Eco1	
CIMG_00497	3.48	2.14E-15	3.93	1.49E-14	nucleolar essential protein 1 (EMG1/NEP1 methyltransferase)	
CIMG_00502	1.90	0.00007	4.07	9.64E-15	glutaryl-CoA dehydrogenase	S
CIMG_00509	10.38	5.59E-37	37.44	1.15E-58	conserved hypothetical protein	G *
CIMG_00530	1.93	0.00002	1.67	0.01011	conserved hypothetical protein	
CIMG_00533	4.97	8.21E-19	12.76	1.37E-31	ABC transporter	
CIMG_00534	5.72	5.00E-20	33.02	2.17E-45	glycerate kinase	
CIMG_00535	1.80	0.00003	8.97	1.12E-29	MFS multidrug transporter	
CIMG_00539	1.94	0.00002	2.13	4.17E-07	IFRD domain-containing protein	

*: Predicted secreted;
+: Gene under positive selection [19]; G: Gene gained in Coccidioides [19]
H, S: Up-regulated in Hyphae or Spherule by microarray [18]

Table S2: All genes up-regulated in parasitic phase

CIMG_00549	3.98	0.00010	3.52	0.00093	conserved hypothetical protein	+
CIMG_00551	1.98	0.00001	2.96	1.31E-07	conserved hypothetical protein (Glycosyltransferase family 28 C-terminal domain)	
CIMG_00553	3.48	2.66E-09	2.58	1.68E-06	conserved hypothetical protein (Tat pathway signal sequence)	
CIMG_00554	4.01	0.00001	6.40	6.81E-12	conserved hypothetical protein	
CIMG_00577	2.92	4.11E-10	3.75	1.33E-12	hypothetical protein	
CIMG_00580	2.31	3.93E-08	3.41	4.04E-10	conserved hypothetical protein (COM1)	
CIMG_00596	1.57	0.00193	1.59	0.00997	conserved hypothetical protein (DUF1686)	
CIMG_00601	5.29	1.66E-06	11.76	1.63E-14	conserved hypothetical protein	
CIMG_00612	5.07	0.00009	7.15	9.95E-06	conserved hypothetical protein	
CIMG_00618	2.65	2.56E-06	2.10	0.00098	ubiquitin carboxyl-terminal hydrolase 12	
CIMG_00622	2.46	1.87E-08	1.59	0.00623	ubiquinone biosynthesis monooxgenase	
CIMG_00628	1.69	0.01665	2.21	0.00417	conserved hypothetical protein (MYND finger)	
CIMG_00630	2.00	0.00001	2.09	0.00417	lysine-specific histone demethylase Aof2	
CIMG_00631	1.38	0.01886	2.02	0.00039	golgi apparatus membrane protein tvp38	
CIMG_00633	1.91	0.00004	3.74	2.51E-13	citrate lyase beta subunit	
CIMG_00637	2.67	1.31E-09	1.65	0.02417	conserved hypothetical protein	
CIMG_00638	11.12	2.57E-34	3.44	3.44E-10	zinc knuckle transcription factor	
CIMG_00641	2.41	2.37E-08	19.85	3.45E-35	conserved hypothetical protein	
CIMG_00643	2.81	1.19E-11	5.91	4.61E-22	SreP protein	
CIMG_00646	12.17	8.57E-36	15.42	5.06E-40	conserved hypothetical protein	
CIMG_00648	2.26	0.00561	4.54	6.26E-07	hypothetical protein	
CIMG_00673	3.30	1.97E-09	2.35	0.00004	WD repeat protein	
CIMG_00682	3.21	0.00006	7.20	1.43E-11	conserved hypothetical protein	
CIMG_00683	3.41	9.48E-13	3.80	2.21E-12	conserved hypothetical protein	
CIMG_00686	3.18	1.91E-07	2.09	0.00030	conserved hypothetical protein	
CIMG_00687	4.49	3.56E-10	2.67	0.00038	conserved hypothetical protein	
CIMG_00700	1.41	0.00976	2.73	1.40E-09	conserved hypothetical protein (histone-like transcription factor)	
CIMG_00702	5.00	3.47E-09	5.34	1.15E-14	pyridoxamine phosphate oxidase	
CIMG_00703	2.56	0.00013	2.29	2.85E-06	pentatricopeptide repeat protein	
CIMG_00704	4.23	4.03E-15	5.57	1.39E-19	vacuolar membrane-associated protein IML1	
CIMG_00708	2.40	3.03E-06	2.31	1.87E-07	conserved hypothetical protein	
CIMG_00712	4.96	6.63E-16	2.13	0.00087	conserved hypothetical protein (Helicase conserved C-terminal domain, SNF2 family N-terminal domain)	
CIMG_00715	1.75	0.00022	4.08	8.01E-18	conserved hypothetical protein	
CIMG_00722	2.85	0.00001	1.83	0.03304	oxidoreductase	
CIMG_00724	1.94	0.00003	1.72	0.00131	Sec1 family superfamily	
CIMG_00760	4.37	2.99E-19	2.18	0.00019	extracellular phytase	*
CIMG_00776	1.98	0.00517	2.87	4.19E-06	conserved hypothetical protein	
CIMG_00794	2.07	4.04E-06	2.50	3.06E-06	conserved hypothetical protein	
CIMG_00801	3.01	1.67E-12	14.51	1.34E-39	GDSL Lipase/Acylhydrolase	
CIMG_00815	2.70	2.71E-11	2.49	0.00003	high-affinity nickel transporter nic1	
CIMG_00816	2.26	0.00001	3.05	2.79E-06	conserved hypothetical protein	
CIMG_00827	1.42	0.04451	1.61	0.00005	acetyl-CoA carboxylase	
CIMG_00834	1.97	3.00E-06	1.48	0.00135	1-acylglycerol-3-phosphate O-acyltransferase	
CIMG_00849	1.67	0.00066	1.51	0.01650	transcription factor TFIIE complex, alpha subunit	
CIMG_00861	1.79	0.00007	3.32	1.97E-10	glutathione S-transferase	
CIMG_00867	1.89	0.00002	3.05	7.32E-10	U3 small nucleolar ribonucleoprotein IMP3	
CIMG_00869	2.30	0.00001	3.21	1.73E-08	conserved hypothetical protein	
CIMG_00877	1.97	0.01693	2.24	0.00244	di/tri peptide transporter 2	
CIMG_00899	4.15	4.08E-16	2.21	5.84E-06	CorA family metal ion transporter	
CIMG_00921	1.98	0.00429	1.98	0.01017	conserved hypothetical protein	
CIMG_00922	2.23	4.56E-08	3.13	4.17E-11	conserved hypothetical protein (Patatin-like phospholipase)	
CIMG_00928	1.52	0.00689	1.72	0.00011	Ras2	
CIMG_00937	4.64	2.89E-12	2.54	0.00003	conserved hypothetical protein	
CIMG_00942	2.24	0.00027	2.26	0.00067	conserved hypothetical protein	
CIMG_00945	1.63	0.00447	1.71	0.01397	cyclin-dependent protein kinase complex component	
CIMG_00962	1.95	0.00002	1.55	0.01118	RNA polymerase II transcriptional coactivator	
CIMG_00963	3.04	5.46E-11	1.98	0.00036	conserved hypothetical protein (dual specificity phosphatase catalytic domain)	
CIMG_00973	1.66	0.01346	1.78	0.01690	autophagy protein Atg22	
CIMG_00977	2.05	0.00001	1.89	0.00027	conserved hypothetical protein (TFIIIC subunit)	
CIMG_00982	1.62	0.00113	2.43	9.98E-06	conserved hypothetical protein (pentatricopeptide repeat domain)	
CIMG_00994	2.93	2.02E-06	2.45	0.01159	conserved hypothetical protein	
CIMG_01005	4.41	6.84E-13	1.77	0.02097	conserved hypothetical protein	
CIMG_01007	3.04	4.54E-13	3.19	1.36E-09	MFS multidrug transporter (benomyl/methotrexate resistance protein)	
CIMG_01019	1.35	0.04686	3.07	1.54E-12	arginine N-methyltransferase 2	
CIMG_01020	2.84	4.61E-07	2.52	0.00002	conserved hypothetical protein	
CIMG_01021	3.81	5.30E-13	2.80	2.26E-07	conserved hypothetical protein	*
CIMG_01025	1.34	0.02874	2.80	3.52E-10	NADH-ubiquinone oxidoreductase 24 kDa subunit	
CIMG_01026	3.89	1.18E-17	6.89	4.25E-22	conserved hypothetical protein	
CIMG_01034	1.75	0.00043	1.44	0.02169	conserved hypothetical protein	
CIMG_01036	2.09	1.91E-06	5.33	3.16E-16	taurine catabolism dioxygenase TauD	
CIMG_01037	1.77	0.00025	2.90	5.10E-10	acetate non-utilizing protein 9	
CIMG_01053	2.81	2.00E-11	29.69	1.16E-52	oxidoreductase	
CIMG_01055	3.29	1.83E-06	2.69	0.00047	HIT finger domain-containing protein	
CIMG_01056	1.41	0.02699	1.73	0.00046	conserved hypothetical protein	
CIMG_01058	2.33	2.95E-08	1.95	0.00073	vacuolar fusion protein MON1	
CIMG_01062	2.12	2.33E-06	2.95	2.21E-07	prolyl peptidase	
CIMG_01063	3.33	4.13E-11	5.23	1.89E-17	conserved hypothetical protein	

*: Predicted secreted;
+: Gene under positive selection [19]; G: Gene gained in Coccididioides [19]
H, S: Up-regulated in Hyphae or Spherule by microarray [18]

Table S2: All genes up-regulated in parasitic phase

CIMG_01065	1.64	0.00100	1.96	7.09E-06	conserved hypothetical protein	
CIMG_01067	7.96	2.17E-30	6.24	1.58E-23	conserved hypothetical protein (Acetyltransferase (GNAT) family)	
CIMG_01069	2.67	1.40E-09	2.17	0.00022	fungal-specific transcription factor domain-containing protein	
CIMG_01074	1.83	0.00003	1.80	0.00268	conserved hypothetical protein	
CIMG_01075	3.91	1.85E-14	3.24	0.00010	conserved hypothetical protein (TAP42-like family)	
CIMG_01086	2.17	0.00116	2.28	0.00331	golgi reassembly stacking protein	
CIMG_01087	1.90	0.00023	2.93	3.66E-10	RNA methylase	
CIMG_01093	1.69	0.00136	1.78	0.00670	DNA damage repair protein Mus42	
CIMG_01110	2.32	1.04E-07	1.86	0.00017	conserved hypothetical protein	
CIMG_01117	1.81	0.00040	3.04	3.84E-08	conserved hypothetical protein	
CIMG_01120	2.42	0.00001	51.24	3.45E-48	putative sphingolipid long chain base-responsive protein LSP1	
CIMG_01126	2.25	6.93E-07	1.94	0.00030	conserved hypothetical protein (DUF571)	
CIMG_01128	2.75	1.95E-10	2.59	7.24E-10	conserved hypothetical protein	
CIMG_01138	5.25	0.00001	2.11	0.02252	conserved hypothetical protein	
CIMG_01170	2.36	0.00026	2.74	0.04083	conserved hypothetical protein	
CIMG_01176	2.17	7.33E-07	1.60	0.00241	conserved hypothetical protein (3-hydroxyacyl-CoA dehydrogenase)	
CIMG_01177	5.15	8.88E-16	1.91	0.00008	conserved hypothetical protein (putative flavoprotein)	
CIMG_01195	2.42	6.24E-09	2.69	3.41E-08	xaa-Pro dipeptidase app	
CIMG_01198	1.93	0.00070	3.63	2.12E-09	acetyltransferase (GNAT family)	
CIMG_01207	1.95	0.00058	2.42	0.00010	Fum8p	
CIMG_01209	11.92	3.37E-28	23.03	3.33E-32	SUR2 (fatty acid hydroxylase superfamily)	
CIMG_01210	8.86	2.74E-26	6.55	1.34E-26	conserved hypothetical protein (Alkaline phytoceramidase (aPHC))	
CIMG_01211	9.37	6.01E-32	64.31	1.61E-65	conserved hypothetical protein (Major Facilitator Superfamily)	
CIMG_01213	2.68	3.54E-11	2.07	0.00002	conserved hypothetical protein	
CIMG_01214	2.33	0.00012	1.85	0.00555	conserved hypothetical protein	
CIMG_01227	3.93	2.52E-13	1.84	0.00846	ubiquitin C-terminal hydrolase	
CIMG_01257	5.12	6.49E-21	6.76	2.94E-24	methionine-R-sulfoxide reductase	
CIMG_01259	4.32	0.00005	1.91	0.03834	conserved hypothetical protein	*
CIMG_01260	2.77	0.00001	8.65	8.68E-15	conserved hypothetical protein (developmentally regulated MAPK interacting protein)	*
CIMG_01262	2.47	0.00001	2.91	2.38E-06	conserved hypothetical protein	
CIMG_01273	1.67	0.00063	5.80	1.16E-20	sulfate permease II	
CIMG_01274	1.60	0.00192	2.63	1.14E-07	mRNA cap methyltransferase	
CIMG_01276	4.98	0.00001	5.94	1.51E-13	cyclohexanone monooxygenase	
CIMG_01277	3.98	0.00002	6.10	1.79E-09	conserved hypothetical protein	
CIMG_01287	2.08	0.00001	1.95	0.00204	conserved hypothetical protein (Rap1 Myb domain)	
CIMG_01297	2.67	1.94E-07	1.65	0.00814	CAIB/BAIF family enzyme	
CIMG_01300	2.82	6.98E-11	2.51	9.88E-07	catechol dioxygenase	
CIMG_01301	1.51	0.01149	2.24	0.00007	amidohydrolase	
CIMG_01306	3.12	4.00E-12	2.44	1.32E-06	dynamin family protein	
CIMG_01309	1.64	0.00212	2.63	1.39E-07	cytochrome c oxidase assembly protein	
CIMG_01311	3.37	7.92E-11	4.29	1.02E-09	conserved hypothetical protein	
CIMG_01316	1.36	0.04529	1.91	7.52E-06	biotin synthase	
CIMG_01324	3.83	1.16E-13	10.45	7.92E-35	conserved hypothetical protein	
CIMG_01332	2.38	1.00E-07	4.27	2.38E-13	FMP52	
CIMG_01333	5.05	1.34E-21	13.33	2.78E-40	conserved hypothetical protein	
CIMG_01359	1.40	0.02440	3.32	1.67E-10	NADH-ubiquinone oxidoreductase subunit	
CIMG_01377	3.06	1.53E-09	6.70	5.28E-19	lipase/esterase	
CIMG_01391	2.10	0.00004	2.73	0.00004	conserved hypothetical protein (Major Facilitator Superfamily)	
CIMG_01392	3.50	7.89E-07	4.30	0.00008	conserved hypothetical protein	
CIMG_01393	2.16	1.66E-07	1.73	0.00190	conserved hypothetical protein	
CIMG_01398	2.02	0.00017	1.81	0.00268	extracellular lipase	
CIMG_01402	1.82	0.00005	3.12	3.28E-10	6-hydroxy-D-nicotine oxidase	
CIMG_01411	6.77	6.99E-15	3.92	7.22E-09	conserved hypothetical protein	
CIMG_01424	1.77	0.00032	3.02	9.59E-11	conserved hypothetical protein (Ankyrin repeats, F-box domain)	
CIMG_01439	2.06	0.00002	1.42	0.04155	l-allo-threonine aldolase	
CIMG_01444	15.04	1.32E-40	8.53	4.93E-33	MFS peptide transporter Ptr2 (POT family)	
CIMG_01456	2.81	8.08E-10	3.25	1.31E-10	conserved hypothetical protein	
CIMG_01457	2.39	1.19E-07	2.66	4.73E-07	mitochondrial chaperone Frataxin	
CIMG_01464	1.73	0.00049	2.75	2.23E-08	prenyltransferase alpha subunit repeat protein	
CIMG_01466	3.53	3.75E-13	4.89	2.85E-16	4-hydroxyphenylpyruvate dioxygenase	
CIMG_01467	1.75	0.00187	1.74	0.03616	sister chromatid cohesion protein Ctf8	
CIMG_01472	3.02	6.85E-12	6.34	1.45E-27	iron sulfur assembly protein 1	
CIMG_01476	1.55	0.00182	1.92	0.00010	50S ribosomal protein L24	
CIMG_01478	2.37	1.30E-07	1.55	0.00878	steroid alpha reductase (3-oxo-5-alpha-steroid 4-dehydrogenase)	
CIMG_01485	3.13	0.00001	2.59	0.00054	conserved hypothetical protein	
CIMG_01488	2.62	2.93E-10	4.05	8.68E-15	MFS multidrug transporter	
CIMG_01489	2.10	7.72E-07	2.89	3.58E-08	SNF2 family helicase	
CIMG_01499	3.14	7.04E-09	3.53	1.17E-09	AAA family ATPase	
CIMG_01508	4.26	9.24E-17	16.66	1.36E-35	C2 domain-containing protein	
CIMG_01513	1.69	0.00108	2.59	8.42E-08	conserved hypothetical protein (SET domain-containing protein)	
CIMG_01527	3.89	7.89E-09	2.03	0.00119	hypothetical protein	
CIMG_01556	1.77	0.00004	2.36	6.59E-08	prenyltransferase	
CIMG_01559	3.10	1.61E-08	2.00	0.02190	conserved hypothetical protein	
CIMG_01560	3.19	1.54E-12	1.33	0.01843	conserved hypothetical protein (WW-domain ligand protein)	
CIMG_01562	2.28	3.18E-08	3.91	1.83E-15	cell surface receptor/MFS transporter	
CIMG_01564	1.35	0.03512	2.64	1.71E-07	involucrin repeat protein	
CIMG_01569	2.68	1.67E-09	4.29	1.30E-13	D-isomer specific 2-hydroxyacid dehydrogenase	
CIMG_01577	1.47	0.00780	2.42	6.19E-08	conserved hypothetical protein	
CIMG_01603	2.25	0.00001	4.39	1.85E-10	phosphotransferase enzyme family protein	

*: Predicted secreted;
+: Gene under positive selection [19]; G: Gene gained in Coccididioides [19]
H, S: Up-regulated in Hyphae or Spherule by microarray [18]

Table S2: All genes up-regulated in parasitic phase

CIMG_01609	2.94	4.50E-12	5.10	7.67E-21	glyoxylate reductase	
CIMG_01610	1.49	0.00668	1.72	0.00164	cell cycle control protein	
CIMG_01615	4.32	1.31E-16	2.90	6.08E-08	conserved hypothetical protein	
CIMG_01618	5.85	2.86E-12	4.93	0.00002	conserved hypothetical protein	
CIMG_01623	2.12	9.94E-07	1.82	0.00522	caffeine-induced death protein Cid2	
CIMG_01624	4.91	4.15E-13	3.80	4.19E-08	conserved hypothetical protein	
CIMG_01634	1.45	0.01068	1.43	0.04210	vacuolar protein sorting-associated protein 26	
CIMG_01642	1.64	0.00242	1.41	0.04953	integral membrane protein	
CIMG_01656	1.81	0.00238	2.00	0.02943	conserved hypothetical protein	
CIMG_01657	4.76	1.12E-20	2.48	0.00025	conserved hypothetical protein	
CIMG_01675	2.40	0.00001	2.19	0.00037	conserved hypothetical protein	
CIMG_01702	6.88	1.70E-15	2.86	1.33E-08	CRAL/TRIO domain-containing protein	
CIMG_01703	5.58	1.01E-19	4.76	2.02E-18	conserved hypothetical protein (DASH complex subunit Dam1)	
CIMG_01704	5.32	7.81E-24	1.71	0.00234	ubiquitin C-terminal hydrolase Ubp8	
CIMG_01716	2.10	6.59E-07	1.54	0.01429	uroporphyrinogen-III synthase	
CIMG_01720	1.51	0.04364	2.11	0.00041	conserved hypothetical protein (mitochondrial protein Fmp26)	
CIMG_01725	1.75	0.00018	1.95	0.00095	mitochondrial carrier protein	
CIMG_01732	1.61	0.00052	5.96	1.35E-23	conserved hypothetical protein	
CIMG_01733	3.25	2.02E-10	2.99	2.79E-09	vacuolar sorting protein 4b	
CIMG_01738	1.58	0.00400	3.47	1.39E-12	conserved hypothetical protein	
CIMG_01739	3.86	2.98E-15	2.63	1.65E-08	stress response transcription factor SrrA/Skn7	
CIMG_01742	4.48	9.32E-12	2.03	0.00043	MFS multidrug transporter	
CIMG_01743	1.34	0.04037	1.58	0.00899	NGG1 interacting factor Nif3	
CIMG_01744	1.72	0.00691	1.86	0.02502	golgi transport complex subunit Cog6	
CIMG_01745	2.73	4.50E-09	4.84	6.13E-14	conserved hypothetical protein	
CIMG_01749	10.74	4.56E-10	124.65	1.46E-43	heat shock protein 30	
CIMG_01750	6.59	1.09E-25	5.81	1.70E-17	secreted serine protease (Subtilase family)	*
CIMG_01754	5.41	3.24E-13	5.85	3.99E-15	serine palmitoyltransferase 1	
CIMG_01762	5.80	6.20E-25	4.12	2.95E-16	conserved hypothetical protein (BAH domain, C-5 cytosine-specific DNA methylase)	
CIMG_01769	4.96	1.26E-09	3.22	0.00045	hypothetical protein	*
CIMG_01775	3.06	0.00023	2.89	0.00394	conserved hypothetical protein	
CIMG_01776	3.98	3.73E-13	4.79	5.22E-20	acetamidase/formamidase	
CIMG_01781	4.27	1.23E-19	5.57	7.47E-24	PH domain-containing protein	
CIMG_01783	1.43	0.02015	1.64	0.00229	ubiquitin C-terminal hydrolase	
CIMG_01791	1.74	0.00012	1.47	0.01182	phosphatidylserine synthase	
CIMG_01800	3.84	0.00179	4.21	0.00054	conserved hypothetical protein	
CIMG_01801	4.08	1.28E-13	3.61	1.58E-08	sugar transporter	
CIMG_01803	2.21	5.44E-07	3.01	4.16E-09	conserved hypothetical protein	
CIMG_01809	2.55	6.01E-10	6.32	1.97E-27	glycerol dehydrogenase	
CIMG_01811	6.93	1.13E-27	2.11	0.00077	conserved hypothetical protein (F-box domain-containing protein)	
CIMG_01813	1.49	0.00678	1.66	0.02256	phosducin family protein	
CIMG_01815	1.72	0.01048	2.61	0.00015	conserved hypothetical protein	*
CIMG_01817	4.36	1.47E-11	3.57	1.21E-09	casein kinase I isoform epsilon	
CIMG_01818	3.48	0.00114	4.10	0.01275	conserved hypothetical protein	
CIMG_01823	2.58	0.00287	2.26	0.00825	conserved hypothetical protein	
CIMG_01826	3.43	4.96E-11	3.79	5.89E-09	conserved hypothetical protein	
CIMG_01828	1.74	0.00022	4.81	3.12E-19	conserved hypothetical protein	
CIMG_01837	1.50	0.00881	1.78	0.00348	mitochondrial genome maintenance protein Mgr2	
CIMG_01843	1.80	0.00051	1.62	0.02253	mitotic spindle biogenesis protein Spc19	
CIMG_01845	2.21	1.24E-07	2.72	3.32E-08	conserved hypothetical protein	
CIMG_01846	1.85	0.00064	1.58	0.02088	conserved hypothetical protein	*
CIMG_01852	1.66	0.00043	1.31	0.03128	conserved hypothetical protein (Phenazine biosynthesis-like protein)	
CIMG_01853	3.51	0.00002	2.95	0.00124	hypothetical protein	*
CIMG_01856	2.88	2.73E-08	1.88	0.00078	iron-regulated transporter	
CIMG_01873	1.71	0.00009	4.74	5.32E-13	conserved hypothetical protein	*
CIMG_01881	2.48	4.35E-10	6.55	4.90E-24	conserved hypothetical protein	
CIMG_01888	1.42	0.02045	2.01	0.00005	prefoldin subunit 6	
CIMG_01895	1.36	0.03415	1.57	0.02359	conserved hypothetical protein	
CIMG_01896	2.45	2.59E-06	1.79	0.03239	conserved hypothetical protein	
CIMG_01898	1.88	0.00222	2.16	0.00009	conserved hypothetical protein	
CIMG_01901	4.39	8.94E-14	1.54	0.03685	conserved hypothetical protein (RNA recognition motif.)	
CIMG_01907	7.03	0.00001	9.95	9.37E-10	conserved hypothetical protein	
CIMG_01908	2.77	0.00029	5.48	3.97E-12	conserved hypothetical protein	
CIMG_01910	1.39	0.04658	2.40	0.00002	conserved hypothetical protein (DUF2434)	
CIMG_01914	5.30	0.00006	2.93	0.01096	conserved hypothetical protein	
CIMG_01916	1.55	0.01307	2.03	0.00397	ABC efflux transporter	
CIMG_01920	2.60	0.00897	3.57	0.00240	conserved hypothetical protein	
CIMG_01927	1.53	0.00751	4.33	8.67E-12	hypothetical protein	
CIMG_01932	1.53	0.00230	1.79	0.00247	peptidyl-tRNA hydrolase domain-containing protein	
CIMG_01935	3.92	1.26E-11	10.36	1.05E-25	conserved hypothetical protein	*
CIMG_01943	1.44	0.00456	2.51	3.48E-08	exosome complex exonuclease Rrp6	
CIMG_01951	1.34	0.03725	1.70	0.00710	meiotically up-regulated 65 protein	
CIMG_01959	3.26	4.03E-07	2.64	0.00010	conserved hypothetical protein	
CIMG_01964	4.17	6.46E-13	2.01	0.00354	conserved hypothetical protein (Hemopexin)	
CIMG_01980	1.76	0.00008	1.77	0.00081	conserved hypothetical protein (putative chorismate mutase type II)	*
CIMG_01994	2.36	4.55E-07	3.29	1.82E-10	alpha,alpha-trehalase	
CIMG_02040	1.55	0.00810	1.51	0.04166	conserved hypothetical protein	
CIMG_02047	4.72	6.85E-13	4.43	2.62E-15	trehalose-phosphatase	**S**
CIMG_02063	1.91	4.50E-06	2.34	4.00E-06	aldose 1-epimerase	

*: Predicted secreted;
+: Gene under positive selection [19]; G: Gene gained in Coccidioides [19]
H, S: Up-regulated in Hyphae or Spherule by microarray [18]

Table S2: All genes up-regulated in parasitic phase

CIMG_02065	2.77	8.50E-07	2.15	0.00005	nicotinate-nucleotide pyrophosphorylase	
CIMG_02069	4.05	5.59E-08	4.38	2.21E-08	conserved hypothetical protein	
CIMG_02073	2.68	3.80E-07	1.65	0.01173	prp 8 CRoW domain-containing protein	**G** *
CIMG_02106	1.57	0.01657	1.61	0.00494	conserved hypothetical protein	
CIMG_02125	1.82	0.00009	1.69	0.00334	conserved hypothetical protein	
CIMG_02137	2.03	0.00004	1.91	0.00078	ubiquitin carboxyl-terminal hydrolase, family 1 protein	
CIMG_02159	1.58	0.00188	1.90	0.00102	ribonuclease P complex subunit Pop2	+
CIMG_02178	1.89	0.00233	3.48	5.88E-11	ureidoglycolate hydrolase	**G**
CIMG_02181	1.78	0.00013	1.62	0.01018	serine/threonine protein kinase	
CIMG_02188	3.36	1.31E-09	4.05	1.27E-10	tripeptidyl peptidase SED3	*
CIMG_02189	8.63	1.75E-09	3.85	0.00027	hypothetical protein	
CIMG_02190	1.96	1.65E-06	10.78	1.10E-36	sulfonate biosynthesis enzyme	
CIMG_02195	3.71	0.00010	4.45	5.26E-07	conserved hypothetical protein	
CIMG_02209	2.69	0.00890	6.05	0.00006	conserved hypothetical protein	
CIMG_02212	1.90	0.00001	1.98	0.00152	conserved hypothetical protein (C-x8-C-x5-C-x3-H type zinc finger)	
CIMG_02220	2.40	0.00001	2.14	0.00021	YjeF domain-containing protein (carbohydrate kinase, YjeF homolog)	
CIMG_02228	3.23	0.00100	2.94	0.00102	conserved hypothetical protein	
CIMG_02255	3.19	3.17E-09	2.11	0.00123	conserved hypothetical protein	
CIMG_02256	3.87	6.28E-11	3.15	8.28E-09	conserved hypothetical protein	
CIMG_02272	1.34	0.02853	1.80	0.00171	conserved hypothetical protein	
CIMG_02292	3.55	6.23E-11	13.00	5.30E-32	D2 protein (phosphatidylethanolamine-binding protein)	
CIMG_02305	1.49	0.03775	12.08	1.97E-20	conserved hypothetical protein	
CIMG_02339	1.88	0.00027	5.68	3.31E-18	succinate dehydrogenase flavoprotein subunit	
CIMG_02344	1.60	0.01933	8.52	4.40E-21	conserved hypothetical protein	
CIMG_02367	1.50	0.04058	2.83	6.01E-06	conserved hypothetical protein	
CIMG_02371	1.85	0.00003	1.83	0.00291	conserved hypothetical protein	
CIMG_02373	2.91	1.64E-12	4.80	3.07E-15	protein kinase	
CIMG_02376	1.63	0.00362	1.99	0.00012	conserved hypothetical protein	+
CIMG_02379	1.69	0.00048	1.91	0.00021	3-demethylubiquinone-9 3-methyltransferase	
CIMG_02380	1.77	0.00109	1.73	0.01859	arsenite resistance protein Ars2	
CIMG_02388	5.58	2.70E-06	11.06	5.35E-09	conserved hypothetical protein	
CIMG_02400	15.16	1.17E-36	14.96	8.47E-45	endo 1,5-alpha-arabinase	*
CIMG_02403	5.27	2.77E-09	3.64	7.15E-06	conserved hypothetical protein	
CIMG_02404	2.68	2.57E-06	4.42	8.56E-10	conserved hypothetical protein	
CIMG_02409	9.43	4.66E-26	5.61	3.54E-15	conserved hypothetical protein	
CIMG_02433	1.83	0.00100	2.91	3.48E-07	conserved hypothetical protein (DUF77)	
CIMG_02437	1.81	0.00012	1.58	0.03462	SYF2 splicing factor domain-containing protein	
CIMG_02439	1.53	0.04871	4.40	1.45E-08	conserved hypothetical protein	
CIMG_02469	3.59	2.24E-14	8.49	9.67E-28	conserved hypothetical protein	
CIMG_02470	3.14	1.27E-09	11.72	9.96E-37	NAD binding Rossmann fold oxidoreductase	
CIMG_02481	1.82	0.00013	2.27	0.00001	conserved hypothetical protein	
CIMG_02483	2.34	3.48E-08	3.12	7.21E-10	mitochondrial escape protein 2	
CIMG_02486	1.47	0.01426	2.74	1.37E-06	glutamate synthase	
CIMG_02491	3.63	4.18E-17	124.83	2.08E-89	phosphatidylserine decarboxylase	*
CIMG_02496	5.91	1.44E-22	1.94	0.00063	conserved hypothetical protein (Glyoxalase/Bleomycin resistance protein/Dioxygenase superfamily)	
CIMG_02498	1.66	0.00093	2.05	0.00004	conserved hypothetical protein	
CIMG_02527	2.38	0.00654	2.31	0.00208	conserved hypothetical protein (Ubiquitin carboxyl-terminal hydrolase)	
CIMG_02529	4.08	2.29E-10	6.65	1.98E-18	conserved hypothetical protein	
CIMG_02532	3.05	3.77E-11	2.64	5.41E-08	tRNA methyltransferase	
CIMG_02567	2.09	6.35E-07	3.05	7.26E-11	rRNA processing protein Fcf2	
CIMG_02601	2.02	0.00001	2.99	2.20E-11	mitochondrial folate carrier protein Flx1	
CIMG_02602	4.30	9.90E-16	3.52	1.04E-12	conserved hypothetical protein	
CIMG_02604	3.74	3.64E-11	3.06	3.57E-06	conserved hypothetical protein	
CIMG_02620	2.11	0.00020	1.56	0.04948	conserved hypothetical protein (Ubiquitin related modifier Urm1)	
CIMG_02621	1.47	0.01195	1.54	0.02388	RNA polymerase II Elongator subunit	
CIMG_02623	1.65	0.01213	2.35	0.00006	tRNA isopentenyltransferase	
CIMG_02627	1.97	0.00027	2.00	0.00124	acetamidase	
CIMG_02628	15.76	4.82E-44	36.23	5.58E-56	Arp2/3 complex subunit Arc16	
CIMG_02629	7.94	2.25E-30	3.95	1.98E-12	conserved hypothetical protein (Histidine acid phosphatase)	*
CIMG_02639	1.43	0.01167	2.25	3.16E-06	homoaconitate hydratase	
CIMG_02651	1.62	0.00175	56.84	3.90E-69	conserved hypothetical protein	
CIMG_02660	1.46	0.00439	3.51	1.13E-11	flavin-containing amine oxidasedehydrogenase	
CIMG_02669	6.93	1.30E-27	6.90	6.64E-16	conserved hypothetical protein	
CIMG_02674	2.66	1.42E-08	1.68	0.00414	conserved hypothetical protein (Phosphotransferase enzyme family)	*
CIMG_02682	2.63	8.34E-08	1.98	0.00166	conserved hypothetical protein (K+ channel tetramerisation domain)	
CIMG_02686	2.10	0.00044	1.85	0.02605	conserved hypothetical protein	
CIMG_02687	1.94	0.00001	2.63	1.87E-07	molecular chaperone (ABC1 family, ubiquinone biosynthesis protein coq-8)	
CIMG_02688	3.41	2.17E-15	4.12	4.32E-15	alpha-1,2-mannosyltransferase	*
CIMG_02697	1.72	0.00009	2.83	4.19E-08	pre-rRNA processing protein	
CIMG_02700	1.40	0.03652	1.49	0.02898	40S ribosomal protein S9	
CIMG_02701	6.53	9.78E-15	9.92	2.26E-16	conserved hypothetical protein	
CIMG_02706	2.19	6.81E-07	2.32	3.28E-06	SET domain-containing protein	
CIMG_02711	2.21	5.42E-07	3.98	4.76E-19	hydrolase	
CIMG_02721	7.43	2.20E-30	3.64	2.35E-09	conserved hypothetical protein	
CIMG_02793	1.44	0.03987	5.24	1.68E-20	conserved hypothetical protein (GPR1/FUN34/yaaH family)	
CIMG_02797	1.74	0.00035	2.44	7.18E-08	mitochondrial mRNA processing protein PET127	
CIMG_02798	4.05	9.26E-16	4.76	1.17E-19	leucine rich repeat protein	
CIMG_02801	1.83	0.00019	4.34	1.44E-18	conserved hypothetical protein (alpha/beta hydrolase fold)	
CIMG_02804	4.55	6.42E-13	2.15	0.00091	conserved hypothetical protein	**G**

*: Predicted secreted;
+: Gene under positive selection [19]; G: Gene gained in Coccidioides [19]
H, S: Up-regulated in Hyphae or Spherule by microarray [18]

Table S2: All genes up-regulated in parasitic phase

CIMG_02805	3.77	1.99E-10	2.67	0.00080	conserved hypothetical protein	
CIMG_02810	2.28	0.00001	1.53	0.02246	conserved hypothetical protein	*
CIMG_02819	2.76	3.31E-08	2.36	4.32E-06	conserved hypothetical protein	
CIMG_02830	2.67	1.96E-10	2.49	3.65E-10	acyl-CoA dehydrogenase	
CIMG_02831	2.56	7.25E-08	2.40	0.00002	cwl1	
CIMG_02835	4.05	9.47E-07	9.83	5.14E-17	conserved hypothetical protein	*
CIMG_02847	1.88	0.00014	1.74	0.00442	conserved hypothetical protein	
CIMG_02866	1.74	0.00189	4.71	1.16E-11	conserved hypothetical protein	
CIMG_02878	1.49	0.02147	3.98	9.24E-15	conserved hypothetical protein	
CIMG_02885	1.98	1.16E-06	4.93	4.50E-18	conserved hypothetical protein (putative oxidoreductase)	
CIMG_02886	2.40	0.00006	10.16	2.20E-15	conserved hypothetical protein	
CIMG_02887	1.38	0.01522	1.49	0.02658	U3 small nucleolar ribonucleoprotein Lcp5	
CIMG_02923	2.89	9.01E-11	2.82	1.35E-08	SET domain-containing protein	
CIMG_02985	4.18	3.26E-12	2.37	0.00003	SUR2 (fatty acid hydroxylase superfamily)	
CIMG_02993	1.50	0.00995	1.67	0.00750	ATP-dependent RNA helicase dbp6	
CIMG_03001	5.95	2.33E-26	9.51	1.12E-29	sugar transporter hexose transporter	
CIMG_03002	5.56	9.78E-12	2.99	0.00091	conserved hypothetical protein	
CIMG_03007	1.75	0.00010	1.58	0.02088	protein kinase	
CIMG_03011	4.79	1.60E-07	2.85	0.01251	conserved hypothetical protein	
CIMG_03015	2.48	0.00014	1.64	0.01580	conserved hypothetical protein	
CIMG_03018	1.53	0.01481	2.92	1.72E-06	conserved hypothetical protein	
CIMG_03027	4.87	2.19E-18	9.07	4.40E-11	conserved hypothetical protein (Phosphotransferase enzyme family)	
CIMG_03048	2.39	9.36E-09	1.78	0.00331	GTP-binding protein GTR2	
CIMG_03049	1.77	0.00028	1.85	0.00141	heme/steroid binding domain-containing protein	
CIMG_03055	2.34	0.00008	2.67	0.00007	vacuolar protein sorting-associated protein Vps28	
CIMG_03061	1.72	0.00007	192.69	2.68E-80	conserved hypothetical protein	
CIMG_03067	2.22	8.76E-08	2.79	5.41E-08	CHIP6 (glycosyltransferase family 28)	
CIMG_03077	5.72	2.95E-08	4.45	4.54E-07	conserved hypothetical protein	
CIMG_03078	3.09	4.51E-07	6.33	1.81E-15	conserved hypothetical protein	
CIMG_03083	9.13	2.63E-25	8.41	8.77E-29	auxin efflux carrier superfamily	
CIMG_03084	2.50	0.00982	5.04	8.33E-06	conserved hypothetical protein	
CIMG_03085	1.92	0.00165	2.89	3.50E-07	conserved hypothetical protein	
CIMG_03098	5.17	6.26E-08	3.18	0.00011	conserved hypothetical protein	
CIMG_03101	2.08	0.00017	1.92	0.00548	conserved hypothetical protein	
CIMG_03103	2.26	3.10E-08	1.73	0.00915	protein phosphatase 2c	
CIMG_03110	2.52	1.24E-07	2.78	2.75E-07	phosphatidylserine decarboxylase	
CIMG_03116	1.58	0.00148	1.79	0.00363	conserved hypothetical protein	
CIMG_03119	1.95	0.04077	13.77	3.09E-21	conserved hypothetical protein	
CIMG_03125	2.16	0.00018	3.63	4.66E-08	conserved hypothetical protein	
CIMG_03131	2.79	7.64E-12	4.29	1.64E-15	conserved hypothetical protein	
CIMG_03134	1.53	0.00228	6.71	1.27E-23	succinate dehydrogenase subunit CybS	
CIMG_03135	2.00	0.00006	3.37	4.42E-10	oxidoreductase	
CIMG_03138	3.92	5.24E-12	8.93	2.21E-18	nucleoside-diphosphate-sugar epimerase	
CIMG_03140	1.91	0.00929	5.73	4.24E-10	conserved hypothetical protein (malate dehydrogenase)	* +
CIMG_03142	1.77	0.00391	2.60	0.00045	alpha-	
CIMG_03144	1.73	0.01803	2.04	0.00217	quinone oxidoreductase	
CIMG_03149	3.16	0.00006	4.15	2.82E-06	MFS multidrug transporter (benomyl/methotrexate resistance protein)	
CIMG_03178	2.81	1.02E-10	2.68	2.12E-10	conserved hypothetical protein	
CIMG_03179	3.86	2.52E-16	1.62	0.00047	conserved hypothetical protein	
CIMG_03184	2.97	1.66E-08	2.24	0.00021	conserved hypothetical protein (F-box domain)	
CIMG_03186	1.66	0.00104	17.69	1.13E-44	metalloreductase	
CIMG_03188	2.06	8.10E-07	3.15	4.11E-10	2-deoxy-D-gluconate 3-dehydrogenase	
CIMG_03192	1.42	0.00524	2.75	5.25E-09	zinc-dependent alcohol dehydrogenase	
CIMG_03193	1.46	0.01159	5.11	1.55E-18	hydroxyisocaproate dehydrogenase	
CIMG_03197	3.60	7.40E-14	5.11	1.63E-20	catechol dioxygenase	
CIMG_03204	1.93	0.00001	1.69	0.00138	UBX domain-containing protein	
CIMG_03207	2.49	9.11E-10	6.11	8.30E-23	conserved hypothetical protein	
CIMG_03209	1.46	0.03158	2.08	0.00015	homoserine O-acetyltransferase	
CIMG_03212	1.93	0.00919	12.31	1.03E-39	conserved hypothetical protein	+
CIMG_03215	1.74	0.00032	2.29	0.00001	UPF0586 protein	
CIMG_03216	1.83	0.00066	2.11	0.00002	conserved hypothetical protein	
CIMG_03221	1.73	0.00146	2.22	0.00003	prefoldin subunit 4	
CIMG_03222	3.10	2.68E-08	4.12	2.44E-18	conserved hypothetical protein	
CIMG_03223	1.70	0.00142	1.63	5.86E-06	heat shock protein CLPA	
CIMG_03227	32.95	8.19E-35	5.05	2.99E-06	conserved hypothetical protein	
CIMG_03233	2.14	2.64E-06	10.76	1.96E-30	conserved hypothetical protein (Phosphotransferase enzyme family)	
CIMG_03242	1.34	0.04936	1.74	0.00167	fibronectin type III domain-containing protein	
CIMG_03250	4.64	0.00027	2.63	0.04461	conserved hypothetical protein	
CIMG_03255	1.40	0.01947	2.89	9.67E-10	C2H2 transcription factor	
CIMG_03278	3.22	0.00010	3.66	7.46E-06	conserved hypothetical protein	
CIMG_03281	2.14	0.00002	1.47	0.03000	endosomal SPRY domain-containing protein	
CIMG_03282	2.32	4.28E-06	2.17	4.52E-06	peptidase D	
CIMG_03283	5.30	3.83E-13	8.52	3.58E-26	conserved hypothetical protein	
CIMG_03289	4.02	1.35E-12	1.50	0.03452	conserved hypothetical protein (Fungal specific transcription factor domain)	
CIMG_03300	4.36	0.00020	4.61	6.72E-08	conserved hypothetical protein	
CIMG_03329	1.91	0.00003	3.19	2.23E-09	JmjC domain-containing protein	
CIMG_03332	2.54	0.01355	4.65	0.00010	conserved hypothetical protein	
CIMG_03342	1.47	0.02033	2.23	4.45E-06	DUF614 domain-containing protein	
CIMG_03347	1.77	0.00134	1.58	0.04068	hypothetical protein	
CIMG_03352	3.95	2.84E-14	5.39	1.16E-14	short chain dehydrogenase	

*: Predicted secreted;
+: Gene under positive selection [19]; G: Gene gained in Coccididioides [19]
H, S: Up-regulated in Hyphae or Spherule by microarray [18]

Table S2: All genes up-regulated in parasitic phase

CIMG_03358	4.92	6.11E-13	2.88	8.91E-07	F-box domain-containing protein	**G**
CIMG_03372	2.88	2.02E-11	1.63	0.01156	conserved hypothetical protein (RING finger, C-x8-C-x5-C-x3-H type zinc finger)	
CIMG_03373	3.25	1.82E-10	4.39	1.10E-12	conserved hypothetical protein (putative ATP adenylyltransferase)	
CIMG_03380	3.20	1.20E-13	2.72	3.12E-10	snoRNP assembly factor Naf1	
CIMG_03383	1.63	0.00703	3.12	2.51E-14	C2H2 transcription factor	
CIMG_03396	1.94	0.00002	1.73	0.00090	WD repeat protein	
CIMG_03401	3.38	7.39E-14	3.41	1.68E-09	cell division protein kinase	
CIMG_03413	2.98	4.42E-13	8.86	1.05E-29	TBC domain-containing protein	
CIMG_03426	2.33	4.32E-06	7.87	3.84E-19	conserved hypothetical protein (DUF1799)	
CIMG_03427	1.58	0.00280	1.83	0.00074	sensor histidine kinase/response regulator	
CIMG_03428	2.50	1.03E-10	7.45	4.03E-23	allantoicase Alc	
CIMG_03429	3.14	5.80E-14	9.86	1.09E-29	cytochrome b561	
CIMG_03430	1.36	0.02990	1.85	0.00025	seryl-tRNA synthetase	
CIMG_03449	1.68	0.00050	4.68	6.75E-07	conserved hypothetical protein	
CIMG_03458	6.95	3.54E-15	3.75	1.38E-08	conserved hypothetical protein	
CIMG_03460	2.44	3.64E-08	2.56	3.66E-06	mitochondrial chaperone BCS1	
CIMG_03465	4.47	1.06E-21	3.05	1.85E-06	conserved hypothetical protein	
CIMG_03467	1.31	0.04069	2.47	1.15E-06	conserved hypothetical protein	
CIMG_03470	4.23	2.89E-10	2.15	0.00161	conserved hypothetical protein	
CIMG_03477	2.45	0.00017	2.66	0.00025	hypothetical protein	
CIMG_03510	1.77	0.00022	1.56	0.01542	conserved hypothetical protein	
CIMG_03520	1.41	0.03878	3.43	1.67E-08	pathogenesis associated protein Cap20	
CIMG_03524	3.48	1.08E-08	2.75	5.91E-06	CAIB/BAIF family enzyme	
CIMG_03547	2.53	4.15E-10	2.77	5.66E-09	phosphoglycerate mutase family domain-containing protein	
CIMG_03548	1.76	0.00019	1.90	0.00030	conserved hypothetical protein (Dor1-like family)	
CIMG_03551	3.66	2.46E-13	4.04	8.34E-10	conserved hypothetical protein	
CIMG_03569	8.71	9.80E-11	9.43	1.26E-12	conserved hypothetical protein	*
CIMG_03575	1.70	0.00044	2.03	0.00021	conserved hypothetical protein (Putative adipose-regulatory protein (Seipin))	
CIMG_03594	2.15	2.73E-07	1.56	0.02933	esterase	
CIMG_03609	1.37	0.03453	2.04	0.00110	DNA damage response protein	
CIMG_03612	1.71	0.00007	16.92	3.55E-47	conserved hypothetical protein	
CIMG_03613	2.11	2.99E-06	1.50	0.02832	PQ loop repeat protein	
CIMG_03614	2.24	0.00002	7.31	1.61E-23	conserved hypothetical protein (Putative stress-responsive nuclear envelope protein)	*
CIMG_03617	3.05	0.00001	6.15	3.95E-11	hypothetical protein	*
CIMG_03621	2.19	3.66E-06	2.51	2.25E-07	conserved hypothetical protein	
CIMG_03627	1.45	0.01261	1.97	0.01467	transcription initiation factor IIB	
CIMG_03630	2.67	1.42E-10	3.38	1.49E-11	conserved hypothetical protein	
CIMG_03636	1.47	0.03235	1.84	0.00040	conserved hypothetical protein (putative acetyltransferase)	
CIMG_03664	1.78	0.00570	4.33	1.37E-09	phosphotransferase enzyme family protein	
CIMG_03668	2.70	0.00233	8.51	2.25E-08	conserved hypothetical protein	
CIMG_03674	5.17	2.99E-13	27.89	2.65E-40	conserved hypothetical protein	
CIMG_03676	3.38	5.12E-07	2.61	0.00118	conserved hypothetical protein	
CIMG_03677	1.52	0.00857	1.63	0.01963	mitochondrial methylglutaconyl-CoA hydratase	
CIMG_03691	2.54	0.00006	4.30	6.75E-06	conserved hypothetical protein	
CIMG_03702	2.83	6.06E-10	3.88	4.63E-16	BAG domain-containing protein	
CIMG_03706	1.55	0.01491	1.58	0.04194	conserved hypothetical protein (putative chalcone-flavanone isomerase)	
CIMG_03707	1.39	0.04834	3.42	1.96E-09	conserved hypothetical protein	
CIMG_03717	1.37	0.03255	2.02	0.00017	RNA polymerase II mediator complex subunit Soh1	
CIMG_03730	2.09	0.00088	7.94	1.15E-23	hypothetical protein	
CIMG_03743	4.40	0.00002	3.02	0.00002	conserved hypothetical protein	
CIMG_03755	3.62	1.14E-16	7.26	4.25E-26	phosphoketolase	
CIMG_03756	2.77	1.63E-12	8.07	2.03E-26	acetate kinase	
CIMG_03758	2.68	0.00025	2.07	0.03685	conserved hypothetical protein	
CIMG_03760	1.43	0.01592	3.13	1.01E-10	conserved hypothetical protein	
CIMG_03762	1.51	0.01728	3.43	2.61E-11	conserved hypothetical protein	
CIMG_03764	7.12	1.34E-06	4.80	0.00001	conserved hypothetical protein	
CIMG_03766	3.54	8.20E-09	2.18	0.00079	ferric-chelate reductase	*
CIMG_03770	1.50	0.00660	2.05	7.02E-06	ATP-dependent RNA helicase DBP8	
CIMG_03771	3.84	0.00038	1.84	0.03124	conserved hypothetical protein	
CIMG_03781	10.31	1.83E-35	5.22	4.26E-11	conserved hypothetical protein	
CIMG_03789	4.43	1.09E-09	18.08	1.31E-26	pyruvate decarboxylase	
CIMG_03790	2.33	5.93E-09	5.20	3.66E-21	THUMP domain-containing protein	
CIMG_03796	3.82	0.00125	2.55	0.01065	conserved hypothetical protein	
CIMG_03802	2.75	1.53E-09	2.87	2.39E-09	glutamate decarboxylase	
CIMG_03803	1.81	0.00010	1.89	0.00033	RINT-1 family protein	
CIMG_03804	1.83	0.00177	2.30	0.00024	DNA ligase I	
CIMG_03805	6.65	3.01E-10	62.25	8.62E-41	macrophage binding protein	
CIMG_03817	1.81	0.00021	1.88	0.00304	vacuolar assembly protein	
CIMG_03819	5.78	1.38E-18	9.00	2.77E-21	conserved hypothetical protein	
CIMG_03822	3.08	4.25E-10	2.88	3.89E-08	chitinase 7	**S**
CIMG_03842	2.58	0.00003	2.69	0.00003	conserved hypothetical protein	
CIMG_03856	2.24	3.01E-07	1.41	0.04420	RNA polymerase II mediator complex protein Nut2	
CIMG_03860	8.19	5.54E-18	11.35	1.29E-20	conserved hypothetical protein	
CIMG_03862	9.08	3.95E-19	3.38	1.33E-08	conserved hypothetical protein	
CIMG_03877	1.60	0.00358	2.21	0.00002	NADH-ubiquinone oxidoreductase 11.5kD subunit	
CIMG_03880	2.95	8.35E-09	19.02	2.35E-38	erythromycin esterase	
CIMG_03881	6.88	7.21E-26	10.74	5.27E-32	conserved hypothetical protein	

*: Predicted secreted;
+: Gene under positive selection [19]; G: Gene gained in Coccidioides [19]
H, S: Up-regulated in Hyphae or Spherule by microarray [18]

Table S2: All genes up-regulated in parasitic phase

CIMG_03882	1.99	4.54E-06	1.90	0.00009	inositol phospholipid biosynthesis protein Scs3	
CIMG_03886	1.43	0.03275	2.16	0.00019	conserved hypothetical protein	
CIMG_03895	3.12	4.00E-08	2.51	0.00186	tRNA-specific adenosine deaminase	
CIMG_03896	1.55	0.00524	1.63	0.00468	splicing factor 3B, subunit 10 (SF3b10)	
CIMG_03897	1.51	0.00520	5.92	3.88E-20	conserved hypothetical protein (putative phage head-tail adaptor)	
CIMG_03899	2.65	0.00109	5.96	0.00005	conserved hypothetical protein	
CIMG_03901	1.40	0.03777	3.73	6.34E-13	conserved hypothetical protein (putative oestrogen-responsive protein Fam102A-B)	
CIMG_03907	4.26	4.86E-19	1.53	0.02514	glycosyl transferase family 8 protein	
CIMG_03909	4.92	6.32E-23	5.54	4.74E-21	alpha/beta hydrolase	*
CIMG_03914	2.74	0.00014	6.34	5.58E-09	conserved hypothetical protein	
CIMG_03938	2.61	0.00122	5.37	4.38E-08	conserved hypothetical protein	
CIMG_03948	1.39	0.02175	1.81	0.00032	TBC domain-containing protein	
CIMG_03952	1.42	0.01474	2.09	0.00016	Phospholipid:diacylglycerol acyltransferase	
CIMG_03956	18.84	7.28E-24	2.40	4.44E-07	carboxylic acid transporter	
CIMG_03959	2.42	3.18E-06	7.54	3.62E-12	conserved hypothetical protein	
CIMG_03960	2.50	1.14E-07	3.30	7.11E-10	conserved hypothetical protein	
CIMG_03961	2.86	7.84E-12	1.60	0.01914	cAMP-dependent protein kinase regulatory subunit	
CIMG_03962	2.26	1.34E-07	1.52	0.01587	conserved hypothetical protein	
CIMG_03969	2.52	5.11E-09	2.47	6.13E-07	conserved hypothetical protein	
CIMG_03971	4.56	1.39E-09	4.24	7.85E-06	conserved hypothetical protein	
CIMG_03990	5.34	4.97E-08	4.60	0.00002	conserved hypothetical protein	
CIMG_03992	2.82	0.04836	4.28	3.39E-06	conserved hypothetical protein	*
CIMG_03996	1.42	0.00897	6.22	7.70E-24	NADH-ubiquinone oxidoreductase 23 kDa subunit	
CIMG_03998	1.57	0.00362	2.96	1.16E-09	vacuolar protein sorting-associated protein 74	
CIMG_03999	1.61	0.00048	3.33	1.28E-11	conserved hypothetical protein	
CIMG_04007	1.55	0.00329	1.54	0.01738	RLI and DUF367 domain-containing protein	
CIMG_04016	1.83	0.00007	3.67	4.42E-14	aconitate hydratase	
CIMG_04019	1.38	0.01155	3.64	1.43E-14	vacuolar membrane PQ loop repeat protein	
CIMG_04020	3.46	0.00038	3.96	0.00049	conserved hypothetical protein	G
CIMG_04040	1.85	0.02480	3.33	0.00065	conserved hypothetical protein	
CIMG_04053	4.71	2.71E-18	4.73	7.54E-12	conserved hypothetical protein	
CIMG_04055	1.48	0.01083	3.38	4.02E-10	conserved hypothetical protein	
CIMG_04068	3.26	0.00026	3.68	0.00236	conserved hypothetical protein	
CIMG_04085	2.32	1.93E-08	2.96	1.15E-11	conserved hypothetical protein	
CIMG_04092	1.61	0.00171	2.46	6.13E-07	conserved hypothetical protein	
CIMG_04096	11.10	5.43E-35	14.73	5.13E-43	conserved hypothetical protein	
CIMG_04100	1.47	0.01315	2.24	3.26E-07	conserved hypothetical protein	
CIMG_04136	1.79	0.01460	2.35	0.00117	conserved hypothetical protein	
CIMG_04137	2.66	1.64E-10	3.94	2.83E-14	tyrosine phosphatase	
CIMG_04150	2.20	0.00008	2.21	0.00004	conserved hypothetical protein	
CIMG_04172	2.35	0.00001	1.93	0.00013	bZIP transcription factor	
CIMG_04183	1.67	0.00022	4.24	3.71E-18	longevity-assurance protein	
CIMG_04192	1.49	0.03451	1.72	0.02507	conserved hypothetical protein	
CIMG_04215	3.24	3.68E-10	1.69	0.01559	conserved hypothetical protein	
CIMG_04228	1.58	0.00464	2.14	0.00043	cytochrome oxidase assembly	
CIMG_04236	1.59	0.00088	2.94	7.10E-10	conserved hypothetical protein	
CIMG_04240	1.73	0.00034	1.99	0.00068	conserved hypothetical protein (Sedlin N-terminal conserved region)	
CIMG_04267	2.64	2.77E-10	1.46	0.02417	conserved hypothetical protein	
CIMG_04306	1.46	0.03136	1.76	0.00393	nucleic acid-binding protein	
CIMG_04316	2.76	2.38E-10	2.68	9.37E-09	conserved hypothetical protein	
CIMG_04331	4.53	2.77E-14	29.86	3.20E-48	conserved hypothetical protein	
CIMG_04336	5.75	5.04E-09	5.52	1.26E-15	catalase	
CIMG_04337	3.86	1.41E-06	6.45	1.27E-12	conserved hypothetical protein	
CIMG_04344	3.94	4.51E-16	1.80	0.00140	uricase	
CIMG_04347	2.19	2.05E-07	2.89	8.52E-09	AAR2 family protein	
CIMG_04349	1.53	0.00633	2.43	5.25E-07	prephenate dehydrogenase	
CIMG_04350	2.70	0.00039	7.13	1.37E-08	conserved hypothetical protein	
CIMG_04351	9.46	1.79E-31	30.67	5.22E-52	cellobiose dehydrogenase	*
CIMG_04354	1.62	0.00357	2.08	0.00056	iron-sulfur cluster assembly 2	
CIMG_04355	8.65	1.81E-15	20.07	1.40E-33	HHE domain-containing protein	
CIMG_04356	5.11	3.02E-24	10.94	1.98E-37	metallo-beta-lactamase superfamily protein	
CIMG_04362	1.82	0.00003	1.55	0.00321	DUF408 domain-containing protein	
CIMG_04367	4.04	3.47E-18	3.22	6.09E-12	conserved hypothetical protein (Methyltransferase domain)	G
CIMG_04370	2.56	2.61E-07	11.09	7.49E-27	conserved hypothetical protein	
CIMG_04373	2.38	2.89E-09	1.52	0.04283	glycerol dehydrogenase	
CIMG_04384	1.78	0.00043	1.70	0.00864	topoisomerase TRF4	
CIMG_04389	2.05	1.92E-06	1.49	0.01718	DUF602 domain-containing protein	
CIMG_04394	1.82	0.00104	2.41	0.00011	conserved hypothetical protein (DUF2458)	
CIMG_04396	3.40	0.00002	8.26	4.49E-09	conserved hypothetical protein	
CIMG_04409	2.64	2.94E-08	3.95	4.30E-11	conserved hypothetical protein	
CIMG_04411	2.88	2.90E-10	5.47	5.47E-17	glucosamine-6-phosphate deaminase	
CIMG_04427	2.43	2.55E-09	4.55	5.01E-17	conserved hypothetical protein	*
CIMG_04428	1.35	0.04561	1.89	0.00167	conserved hypothetical protein	
CIMG_04431	3.00	7.39E-08	4.12	5.56E-12	actin cytoskeleton protein	
CIMG_04434	2.39	3.06E-08	2.31	3.20E-06	antiviral helicase SKI2	
CIMG_04435	2.03	3.48E-06	2.03	0.00006	conserved hypothetical protein (DUF2415)	
CIMG_04439	4.34	3.03E-07	6.63	7.98E-17	conserved hypothetical protein	
CIMG_04448	4.13	4.10E-10	2.44	0.00672	conserved hypothetical protein	
CIMG_04457	2.04	0.00002	3.07	1.70E-06	C6 transcription factor	

*: Predicted secreted;
+: Gene under positive selection [19]; G: Gene gained in Coccidioides [19]
H, S: Up-regulated in Hyphae or Spherule by microarray [18]

Table S2: All genes up-regulated in parasitic phase

CIMG_04459	1.31	0.04138	2.98	6.68E-10	MFS transporter Fmp42	
CIMG_04465	1.87	0.00005	2.10	0.00002	UTP-glucose-1-phosphate uridylyltransferase	
CIMG_04472	2.65	0.00516	3.01	0.00040	conserved hypothetical protein	
CIMG_04496	1.49	0.01533	2.14	0.00074	IMP dehydrogenase	
CIMG_04503	3.69	0.00002	4.38	8.44E-07	conserved hypothetical protein	
CIMG_04504	2.82	0.01562	2.46	0.02402	conserved hypothetical protein	*
CIMG_04513	2.88	0.00131	2.79	0.01077	conserved hypothetical protein	
CIMG_04519	2.28	0.00002	1.52	0.02887	conserved hypothetical protein	
CIMG_04523	1.53	0.00792	2.16	0.00003	C6 transcription factor	
CIMG_04526	1.45	0.02192	1.50	0.04093	import inner membrane translocase subunit tim-50	
CIMG_04531	5.28	5.51E-16	3.35	5.42E-06	polarized growth protein	
CIMG_04532	7.79	5.81E-07	2.90	0.02002	conserved hypothetical protein	
CIMG_04534	1.66	0.00074	2.13	0.00008	conserved hypothetical protein	
CIMG_04536	2.22	0.00004	3.92	1.89E-09	conserved hypothetical protein (Mitochondrial ATPase inhibitor, IATP)	
CIMG_04544	1.72	0.00123	2.14	0.00004	alpha-amylase AmyA	* S
CIMG_04545	2.73	5.04E-08	2.85	1.22E-07	bax Inhibitor family protein	
CIMG_04550	4.53	8.20E-11	116.10	4.32E-68	conserved hypothetical protein	
CIMG_04551	2.08	2.76E-07	140.87	3.25E-73	conserved hypothetical protein (putative conidiation protein 6)	
CIMG_04552	1.76	0.00616	2.35	0.00102	conserved hypothetical protein	
CIMG_04558	1.40	0.01056	3.32	7.38E-06	proline oxidase PrnD	
CIMG_04561	2.65	0.00010	2.69	0.00113	hypothetical protein	
CIMG_04574	2.47	4.77E-07	3.59	3.06E-10	conserved hypothetical protein	G
CIMG_04575	2.98	2.67E-06	3.48	0.00023	MYB DNA-binding domain-containing protein	
CIMG_04576	1.65	0.00860	1.98	0.00059	cytochrome c oxidase polypeptide IV	
CIMG_04583	4.52	4.26E-12	2.72	0.00002	conserved hypothetical protein	
CIMG_04587	6.23	4.50E-06	3.14	0.00541	conserved hypothetical protein	
CIMG_04588	7.02	2.48E-24	4.62	1.60E-14	aldo-keto reductase	
CIMG_04590	1.56	0.00206	1.88	0.00039	dual specificity phosphatase	
CIMG_04591	1.40	0.00890	3.36	1.48E-11	dihydroxyacid dehydratase Ilv3	
CIMG_04592	2.68	3.57E-07	12.12	1.86E-27	conserved hypothetical protein (DUF1772)	
CIMG_04610	3.59	2.25E-13	1.95	0.00089	conserved hypothetical protein (Fungal Zn(2)-Cys(6) binuclear cluster domain)	
CIMG_04613	10.92	5.51E-12	26.33	3.03E-50	hypothetical protein	*
CIMG_04624	1.57	0.00236	1.78	0.00058	prefoldin subunit 2	
CIMG_04634	1.68	0.00023	2.76	7.73E-09	DNA-directed RNA polymerase I and III polypeptide	
CIMG_04635	2.04	3.71E-06	1.63	0.01830	NEDD8 activating enzyme	
CIMG_04661	5.54	4.15E-21	4.82	9.10E-18	conserved hypothetical protein (Fungal Zn(2)-Cys(6) binuclear cluster domain)	
CIMG_04662	9.70	4.93E-18	28.77	9.55E-41	magnesium-translocating P-type ATPase	S
CIMG_04663	1.44	0.02072	4.67	5.75E-18	acetyltransferase	
CIMG_04673	1.72	0.00170	2.79	1.03E-07	8-oxoguanine DNA glycosylase	
CIMG_04678	9.38	7.24E-15	8.47	4.99E-07	conserved hypothetical protein (Fatty acid hydroxylase superfamily)	
CIMG_04680	10.16	2.69E-18	5.49	4.24E-14	conserved hypothetical protein	
CIMG_04682	12.64	7.10E-15	4.43	2.76E-08	conserved hypothetical protein (DUF1857)	G
CIMG_04683	11.48	2.92E-18	20.81	1.80E-23	O-methyltransferase	
CIMG_04684	8.29	2.27E-22	17.52	1.81E-30	pyridoxal reductase	
CIMG_04685	3.23	9.83E-09	3.01	1.37E-08	conserved hypothetical protein (O-methyltransferase)	
CIMG_04687	2.63	2.98E-08	4.19	8.40E-11	conserved hypothetical protein (Myb-like DNA-binding domain)	
CIMG_04691	2.23	2.67E-07	1.45	0.02941	chromate ion transporter	
CIMG_04695	1.37	0.03475	1.82	0.00033	conserved hypothetical protein (Dpy-30 motif)	
CIMG_04710	2.71	1.69E-08	2.04	0.00013	conserved hypothetical protein	
CIMG_04717	3.50	1.24E-15	2.50	1.21E-07	silencing information regulator (Sir2 family)	
CIMG_04723	1.80	0.04049	2.57	0.01304	conserved hypothetical protein	
CIMG_04726	1.50	0.01033	1.88	0.00092	conserved hypothetical protein (Helix-loop-helix DNA-binding domain)	
CIMG_04731	2.99	3.78E-10	12.06	1.18E-37	aspartyl-tRNA synthetase	
CIMG_04732	1.49	0.01771	48.62	1.40E-61	HHE domain-containing protein	
CIMG_04739	3.00	7.87E-12	4.10	1.41E-17	conserved hypothetical protein	
CIMG_04740	18.35	1.99E-48	16.54	5.75E-32	conserved hypothetical protein	
CIMG_04744	1.69	0.00045	1.79	0.00155	conserved hypothetical protein (Fungal family of unknown function (DUF1776))	S
CIMG_04753	2.41	2.31E-06	1.88	0.00002	conserved hypothetical protein	
CIMG_04756	2.36	1.09E-06	6.49	4.41E-16	Y20 protein	
CIMG_04757	1.55	0.02437	12.23	9.46E-16	conserved hypothetical protein (CVNH domain)	
CIMG_04761	1.72	0.01277	1.93	0.01388	chitinase 5	+
CIMG_04763	2.01	0.00251	1.97	0.01167	conserved hypothetical protein	
CIMG_04765	3.06	5.06E-10	38.38	5.73E-54	integral membrane protein	
CIMG_04768	1.63	0.00122	1.57	0.01174	conserved hypothetical protein (Mediator complex protein)	
CIMG_04801	1.67	0.00027	2.89	1.36E-09	conserved hypothetical protein (Protein of unknown function (DUF2414))	
CIMG_04819	4.49	5.44E-17	5.26	2.75E-19	conserved hypothetical protein (Vta1-like)	
CIMG_04858	2.55	0.00011	2.31	0.01419	conserved hypothetical protein (putative ochratoxin A non-ribosomal peptide synthetase)	
CIMG_04862	6.02	8.87E-24	1.84	0.00326	cytochrome P450 monooxygenase	
CIMG_04863	2.83	1.42E-09	2.91	6.70E-07	conserved hypothetical protein (MFS transporter, Tat pathway signal sequence)	
CIMG_04864	2.00	0.00160	2.22	0.00009	flavoprotein subunit P-cresol methylhydroxylase	
CIMG_04869	2.14	0.01499	3.59	0.00002	conserved hypothetical protein	
CIMG_04877	2.42	0.00102	2.87	0.00836	conserved hypothetical protein	
CIMG_04891	2.17	7.00E-07	2.11	0.00002	sulfate permease II	
CIMG_04897	2.79	1.76E-09	2.70	1.30E-06	patatin-like phospholipase domain-containing protein	
CIMG_04912	1.58	0.00117	1.48	0.01579	translocon protein Sec61beta	

*: Predicted secreted;
+: Gene under positive selection [19]; G: Gene gained in Coccididioides [19]
H, S: Up-regulated in Hyphae or Spherule by microarray [18]

Table S2: All genes up-regulated in parasitic phase

CIMG_04916	1.80	0.00305	3.00	3.39E-07	conserved hypothetical protein	
CIMG_04925	1.78	0.00573	3.88	4.20E-09	conserved hypothetical protein (YDG/SRA domain)	
CIMG_04927	2.66	4.70E-10	3.06	7.98E-09	Sin3 complex subunit (SIN3 protein-binding protein STB2)	
CIMG_04933	2.45	0.00160	4.84	0.00019	conserved hypothetical protein	
CIMG_04935	1.60	0.00408	2.38	0.00002	urease	
CIMG_04936	2.92	2.15E-11	3.79	1.04E-10	DNA repair protein	
CIMG_04938	2.30	1.20E-08	2.05	0.00022	conserved hypothetical protein	
CIMG_04950	2.53	5.29E-07	2.30	4.34E-06	conserved hypothetical protein	
CIMG_04952	1.66	0.00197	1.66	0.04320	conserved hypothetical protein	
CIMG_04955	2.02	0.00001	1.68	0.00940	conserved hypothetical protein (FMC1 protein family)	
CIMG_04956	2.61	1.09E-08	2.08	0.00019	HIT finger domain-containing protein	
CIMG_04961	6.21	1.44E-13	2.51	0.00014	conserved hypothetical protein	**G**
CIMG_04979	3.26	3.01E-14	1.64	0.00739	Ser/Thr protein phosphatase	
CIMG_04984	1.56	0.04574	2.64	0.00007	hypothetical protein	
CIMG_04986	2.42	0.00396	2.55	0.00312	conserved hypothetical protein	
CIMG_05000	1.73	0.00056	1.78	0.00391	TBC domain-containing protein	
CIMG_05004	8.81	1.58E-21	9.52	8.78E-25	conserved hypothetical protein	**G**
CIMG_05010	2.71	0.01686	5.18	0.00017	conserved hypothetical protein	
CIMG_05013	2.47	2.85E-09	1.85	0.00122	conserved hypothetical protein (pentatricopeptide repeat domain)	
CIMG_05037	3.80	1.20E-12	2.01	0.00022	conserved hypothetical protein	*****
CIMG_05043	1.94	0.00001	1.51	0.02618	phosphatidylinositol 4-kinase type II subunit alpha	
CIMG_05052	4.90	1.35E-21	2.77	3.80E-07	mitochondrial pyruvate dehydrogenase kinase	
CIMG_05071	5.44	1.38E-18	21.93	8.67E-40	conserved hypothetical protein	
CIMG_05072	2.45	0.00039	1.76	0.02565	hypothetical protein	
CIMG_05078	2.61	3.92E-10	2.18	2.67E-06	extracellular serine-rich protein	*****
CIMG_05085	1.72	0.00048	4.00	2.00E-15	DRAP deaminase	
CIMG_05086	1.76	0.01280	3.52	2.99E-09	conserved hypothetical protein	
CIMG_05095	1.93	0.00004	3.49	3.55E-10	conserved hypothetical protein	
CIMG_05107	2.75	9.55E-11	2.56	1.03E-08	calcineurin binding protein	
CIMG_05109	5.20	7.59E-19	2.71	0.00005	conserved hypothetical protein	
CIMG_05116	1.78	0.00017	2.43	2.15E-06	phenylalanyl-tRNA synthetase, alpha subunit	
CIMG_05122	2.00	0.00001	2.23	0.00003	conserved hypothetical protein (PAPA-1-like conserved region)	
CIMG_05123	1.46	0.00877	1.49	0.00842	conserved hypothetical protein	
CIMG_05163	2.19	4.78E-07	2.03	0.00009	conserved hypothetical protein	
CIMG_05172	11.66	2.61E-38	2.60	0.00004	conserved hypothetical protein (Ankyrin repeat)	
CIMG_05174	1.93	0.00002	1.48	0.04517	NADPH-adrenodoxin reductase Arh1	
CIMG_05193	3.46	0.00087	5.91	8.13E-08	conserved hypothetical protein	
CIMG_05209	3.24	1.33E-13	2.47	6.99E-07	hydroxymethylglutaryl-CoA lyase	
CIMG_05218	1.48	0.01017	1.66	0.00687	mitochondrion organization and biogenesis protein	
CIMG_05221	1.68	0.00098	1.75	0.00135	conserved hypothetical protein	
CIMG_05229	1.69	0.00100	1.70	0.02547	mitochondrial cytochrome c oxidase assembly factor Cox11p	
CIMG_05235	19.55	2.44E-44	35.45	2.80E-52	conserved hypothetical protein	
CIMG_05241	4.02	4.12E-11	2.80	4.86E-07	conserved hypothetical protein	
CIMG_05247	4.41	3.05E-16	9.33	8.28E-27	conserved hypothetical protein	
CIMG_05252	2.24	8.07E-07	3.12	4.84E-09	glycosyl transferase	
CIMG_05255	6.32	5.37E-10	3.12	0.00015	conserved hypothetical protein	
CIMG_05269	1.90	0.00003	2.15	0.00010	kinase activator	
CIMG_05271	2.33	1.15E-06	3.55	4.45E-11	sulfur metabolite repression control protein	
CIMG_05278	1.70	0.00029	1.95	0.00200	conserved hypothetical protein	
CIMG_05288	2.38	0.00001	2.09	0.00087	conserved hypothetical protein	
CIMG_05377	6.64	1.84E-15	18.02	1.01E-28	protein phosphatase regulatory subunit Gac1	
CIMG_05392	1.58	0.00175	1.49	0.02040	conserved hypothetical protein (U3 snoRNA associated)	
CIMG_05394	2.05	0.00012	2.40	6.83E-06	Sad1-interacting factor 2	
CIMG_05402	1.81	0.00054	2.04	0.00144	conserved hypothetical protein (DUF917, Hydantoinase/oxoprolinase N-terminal region)	
CIMG_05406	1.76	0.00021	1.71	0.00849	dual specificity phosphatase	
CIMG_05409	1.97	3.49E-06	2.17	4.67E-06	exocyst complex protein exo70	
CIMG_05415	1.64	0.00043	1.55	0.00480	conserved hypothetical protein	
CIMG_05419	3.70	6.43E-09	2.54	0.00054	conserved hypothetical protein	
CIMG_05424	2.76	2.29E-08	3.41	4.97E-11	conserved hypothetical protein	
CIMG_05442	2.28	1.81E-08	3.25	4.58E-12	phosphoglycerate mutase	
CIMG_05452	1.53	0.01764	1.89	0.00179	phosphoric ester hydrolase	
CIMG_05460	1.76	0.00254	4.97	1.13E-14	conserved hypothetical protein	
CIMG_05471	3.07	1.16E-07	3.57	4.71E-08	conserved hypothetical protein	
CIMG_05475	2.74	2.21E-10	3.61	6.23E-14	DUF1275 domain-containing protein	**+**
CIMG_05484	2.67	6.74E-09	1.59	0.01284	sorting nexin-41	
CIMG_05485	5.01	5.30E-16	3.74	3.39E-08	conserved hypothetical protein	
CIMG_05489	1.72	0.00087	1.57	0.02287	protein kinase	
CIMG_05492	2.47	6.21E-08	2.84	2.52E-09	dihydroxy acid dehydratase Ilv3	
CIMG_05501	5.23	4.11E-17	3.61	7.35E-08	benzoate 4-monooxygenase cytochrome P450	
CIMG_05503	7.96	7.05E-15	3.55	1.59E-06	conserved hypothetical protein (alpha/beta hydrolase fold)	
CIMG_05510	2.02	0.00004	1.70	0.01797	conserved hypothetical protein (Ankyrin repeat)	
CIMG_05516	4.02	1.52E-08	2.04	0.00401	CCCH zinc finger domain-containing protein	
CIMG_05519	2.22	0.00004	2.00	0.00896	conserved hypothetical protein (Fungal Zn(2)-Cys(6) binuclear cluster domain, Fungal specific transcription factor domain)	
CIMG_05526	2.11	5.71E-07	1.57	0.00447	conserved hypothetical protein	
CIMG_05530	2.47	0.00018	3.07	0.00015	conserved hypothetical protein	
CIMG_05533	1.75	0.00132	2.02	0.00084	regulatory protein suaprga1	
CIMG_05539	2.68	0.00002	1.84	0.00244	protein kinase domain-containing protein	
CIMG_05542	1.78	0.00044	1.87	0.00663	hexose carrier protein (MFS transporter)	

*: Predicted secreted;
+: Gene under positive selection [19]; G: Gene gained in Coccidioides [19]
H, S: Up-regulated in Hyphae or Spherule by microarray [18]

Table S2: All genes up-regulated in parasitic phase

CIMG_05543	3.96	3.35E-06	2.08	0.00916	conserved hypothetical protein	
CIMG_05545	3.38	0.00001	7.53	5.00E-08	conserved hypothetical protein	
CIMG_05549	1.91	0.00002	2.90	5.64E-09	metallo-beta-lactamase superfamily protein	
CIMG_05552	2.91	4.32E-11	1.71	0.03153	transmembrane protein UsgS	
CIMG_05564	5.89	7.31E-13	3.32	3.91E-08	conserved hypothetical protein	
CIMG_05575	5.05	0.00012	4.04	0.00041	conserved hypothetical protein	
CIMG_05584	4.70	2.39E-11	2.08	0.00399	conserved hypothetical protein	
CIMG_05585	5.60	8.30E-22	3.93	1.17E-10	conserved hypothetical protein (carbonic anhydrase 2)	*
CIMG_05588	2.16	5.50E-07	2.78	2.55E-08	conserved hypothetical protein	
CIMG_05595	2.22	7.03E-07	1.88	0.00143	ubiquinone biosynthesis protein Coq7	
CIMG_05608	1.76	0.00154	5.43	6.41E-10	conserved hypothetical protein	
CIMG_05609	1.43	0.01255	1.90	0.00012	mitochondrial large ribosomal subunit	
CIMG_05615	2.06	0.00005	2.40	2.96E-06	conserved hypothetical protein (Major facilitator superfamily)	
CIMG_05617	1.77	0.00147	2.00	0.00017	phosphoglycerate mutase	*
CIMG_05622	1.69	0.00015	21.08	1.99E-51	conserved hypothetical protein (DUF2503)	*
CIMG_05627	1.32	0.03190	8.86	1.54E-24	conserved hypothetical protein	
CIMG_05629	1.73	0.00039	1.60	0.02288	conserved hypothetical protein (FHA domain)	
CIMG_05631	2.50	0.00229	10.77	1.73E-13	conserved hypothetical protein	
CIMG_05651	2.63	2.62E-06	3.41	3.20E-07	conserved hypothetical protein	
CIMG_05653	1.94	0.00001	5.68	4.05E-20	syntaxin 5	
CIMG_05654	2.98	1.00E-08	2.48	0.00005	conserved hypothetical protein	
CIMG_05660	4.03	2.50E-14	3.34	2.54E-09	protein kinase	
CIMG_05695	3.66	3.05E-07	2.42	0.00272	conserved hypothetical protein	
CIMG_05723	1.49	0.00950	1.70	0.00103	conserved hypothetical protein (Phosphotransferase enzyme family)	
CIMG_05727	2.20	6.21E-08	2.15	2.27E-08	farnesyltransferase alpha subunit	
CIMG_05729	2.04	9.84E-07	2.20	0.00010	methionyl-tRNA synthetase	
CIMG_05730	2.44	1.26E-09	1.76	0.00126	nuclear transport factor 2 domain-containing protein	
CIMG_05736	1.89	0.02360	2.33	0.01552	metalloproteinase 6	
CIMG_05745	6.71	1.36E-17	4.60	1.96E-13	conserved hypothetical protein (cytoplasm protein)	
CIMG_05749	2.80	0.00007	2.66	0.00003	conserved hypothetical protein	
CIMG_05752	1.76	0.01887	2.34	0.00523	conserved hypothetical protein (ankyrin repeat domain-containing protein 1)	
CIMG_05758	9.83	8.21E-20	38.01	3.01E-52	parasitic phase-specific protein PSP-1	
CIMG_05759	3.42	1.87E-11	6.36	1.74E-16	FAD dependent sulfhydryl oxidase Erv2	
CIMG_05760	1.91	0.00106	1.62	0.00476	conserved hypothetical protein	
CIMG_05769	2.00	0.03113	1.88	0.04734	siderophore iron transporter	
CIMG_05793	1.92	0.00001	1.60	0.00988	mitochondrial inner membrane magnesium transporter mrs2	
CIMG_05803	2.26	1.10E-06	3.08	1.44E-08	conserved hypothetical protein (Phospholipase/Carboxylesterase)	
CIMG_05810	6.10	3.52E-13	6.58	1.56E-16	proteasome subunit beta type-9	
CIMG_05824	2.48	3.96E-09	2.31	6.08E-08	peptidyl-prolyl cis-trans isomerase-like 1	
CIMG_05827	2.10	7.07E-07	2.43	1.76E-06	ribonuclease P complex subunit Pop1	
CIMG_05834	1.46	0.03312	1.93	0.00121	conserved hypothetical protein (phosphotransferase enzyme family)	
CIMG_05852	1.85	0.00004	7.35	2.05E-25	fasciclin domain-containing protein	*
CIMG_05853	1.73	0.00324	2.86	1.24E-06	serine/threonine protein kinase	**S**
CIMG_05873	1.77	0.00018	1.98	0.00061	esterase	
CIMG_05884	1.81	0.00742	1.71	0.01065	rRNA processing protein Ipi1	
CIMG_05892	7.08	4.50E-24	14.40	2.88E-45	conserved hypothetical protein (Methyltransferase domain)	
CIMG_05893	2.89	0.00014	2.76	0.00289	conserved hypothetical protein	
CIMG_05898	2.53	3.56E-10	1.44	0.03904	aldo/keto reductase	
CIMG_05899	3.43	5.86E-14	2.28	5.23E-06	sulfite transporter Ssu1	
CIMG_05904	3.10	4.78E-10	2.42	0.00004	conserved hypothetical protein	
CIMG_05914	3.87	7.48E-14	4.22	7.16E-10	conserved hypothetical protein	
CIMG_05915	1.43	0.01569	1.65	0.00220	mitogen-activated protein kinase HOG1	**S**
CIMG_05918	1.64	0.00061	3.35	4.54E-11	cupin domain-containing protein	**G**
CIMG_05920	2.69	2.83E-11	3.11	2.52E-11	conserved hypothetical protein (DUF2342)	
CIMG_05961	4.58	2.44E-09	5.46	3.69E-10	hypothetical protein	
CIMG_05973	5.12	5.22E-15	4.37	3.53E-13	conserved hypothetical protein (Swi5)	
CIMG_05987	1.44	0.01466	2.56	2.67E-07	DnaJ domain-containing protein Psi	
CIMG_05995	1.72	0.00009	3.41	4.55E-13	4-aminobutyrate aminotransferase	
CIMG_05996	4.11	0.00017	3.39	0.00010	conserved hypothetical protein	
CIMG_06007	1.41	0.02679	2.28	0.00001	filamentation protein	
CIMG_06009	2.89	0.00004	2.03	0.02305	low-temperature viability protein ltv1	
CIMG_06011	2.35	0.00001	2.15	0.00002	C2H2 type zinc finger domain-containing protein	
CIMG_06039	2.85	4.05E-08	23.97	4.38E-44	conserved hypothetical protein	
CIMG_06040	5.62	3.31E-21	8.00	3.81E-20	BAR domain-containing protein	
CIMG_06048	1.89	0.00001	1.49	0.01610	conserved hypothetical protein	
CIMG_06053	5.55	3.65E-08	10.51	4.66E-28	alcohol dehydrogenase I	
CIMG_06054	1.63	0.00290	1.65	0.00646	SNF2 family helicase/ATPase	
CIMG_06062	1.33	0.04058	2.75	2.52E-06	DUF500 domain-containing protein	
CIMG_06064	1.43	0.01923	3.77	1.74E-12	NADPH-dependent FMN/FAD containing oxidoreductase	
CIMG_06068	5.63	1.41E-22	2.02	8.14E-06	DUF300 domain-containing protein	
CIMG_06073	4.56	1.97E-11	2.54	6.11E-06	metalloproteinase 10	*
CIMG_06074	3.83	1.20E-07	3.64	0.00001	conserved hypothetical protein	
CIMG_06075	2.97	1.48E-12	3.21	9.16E-13	conserved hypothetical protein	
CIMG_06080	1.46	0.00890	3.47	1.95E-11	elongation factor G	
CIMG_06088	3.94	2.58E-13	1.87	0.00050	cystathionine beta-synthase	
CIMG_06090	2.58	0.00189	2.91	0.00076	conserved hypothetical protein	
CIMG_06104	2.68	7.01E-11	1.81	0.00044	conserved hypothetical protein	
CIMG_06118	5.04	1.57E-22	4.55	2.03E-19	conserved hypothetical protein	
CIMG_06120	1.61	0.00174	2.63	1.60E-06	conserved hypothetical protein	*

*: Predicted secreted;
+: Gene under positive selection [19]; G: Gene gained in Coccidioides [19]
H, S: Up-regulated in Hyphae or Spherule by microarray [18]

Table S2: All genes up-regulated in parasitic phase

CIMG_06121	2.95	1.23E-12	2.56	5.89E-07	conserved hypothetical protein	
CIMG_06122	1.54	0.00174	2.70	4.16E-07	AMP-binding enzyme	
CIMG_06124	2.81	0.00747	4.85	0.00006	conserved hypothetical protein	
CIMG_06131	4.10	1.61E-09	5.78	7.10E-10	conserved hypothetical protein	G
CIMG_06132	2.04	2.45E-06	2.15	0.00002	SH3 domain signaling protein	
CIMG_06138	2.42	1.21E-08	1.62	0.00350	Ku70	
CIMG_06140	2.10	5.05E-07	1.79	0.00184	pre-mRNA-splicing factor cwc24	
CIMG_06144	1.93	0.02491	7.55	5.18E-07	hypothetical protein	
CIMG_06146	2.22	2.63E-07	3.58	1.37E-12	ferrochelatase	
CIMG_06147	2.29	1.16E-07	2.91	1.37E-09	conserved hypothetical protein (pentatricopeptide repeat domain)	
CIMG_06175	7.49	8.50E-14	10.35	4.55E-11	conserved hypothetical protein	
CIMG_06176	2.14	0.00004	2.51	1.19E-06	N-terminal asparagine amidohydrolase	
CIMG_06182	1.69	0.00404	2.30	0.00072	phospholipase D	*
CIMG_06194	2.96	4.47E-07	2.21	0.00198	conserved hypothetical protein (Glutathione-dependent formaldehyde-activating enzyme)	
CIMG_06198	5.15	1.61E-22	4.89	2.99E-15	conserved hypothetical protein	
CIMG_06203	4.50	1.20E-19	3.55	2.52E-11	conserved hypothetical protein (F-box domain)	
CIMG_06217	1.68	0.00058	2.60	2.14E-07	TRAPP complex subunit	
CIMG_06218	2.60	9.25E-08	2.16	0.00122	serine/threonine protein kinase	
CIMG_06228	1.59	0.00160	2.01	0.00038	poly(p)/ATP NAD kinase	
CIMG_06232	2.81	0.00001	1.70	0.03874	conserved hypothetical protein	
CIMG_06239	1.73	0.00012	2.50	6.44E-07	calcium channel subunit Mid1	*
CIMG_06242	4.95	2.07E-13	4.80	2.52E-07	conserved hypothetical protein	
CIMG_06246	1.48	0.00984	1.68	0.00392	1,3-beta-glucan biosynthesis protein	
CIMG_06250	3.79	1.13E-09	2.79	3.80E-07	conserved hypothetical protein	*
CIMG_06258	1.89	0.02104	2.76	0.00349	conserved hypothetical protein	
CIMG_06261	4.81	8.03E-09	2.59	0.00112	conserved hypothetical protein	
CIMG_06262	22.25	1.54E-27	2.95	5.45E-06	conserved hypothetical protein	
CIMG_06263	3.18	0.00005	16.28	8.11E-32	conserved hypothetical protein	
CIMG_06271	4.51	8.69E-19	1.62	0.01851	conserved hypothetical protein (Chromatin organisation modifier (chromo) domain)	
CIMG_06275	2.20	2.85E-07	1.98	0.00135	glycogen branching enzyme	
CIMG_06290	3.67	0.00001	3.01	0.00150	conserved hypothetical protein	
CIMG_06294	1.61	0.01424	2.55	2.52E-06	citrate synthase	
CIMG_06297	2.11	5.83E-07	1.73	0.00291	DNA-directed RNA polymerase III subunit rpc3	
CIMG_06322	2.38	1.34E-06	1.45	0.04058	conserved hypothetical protein (alpha/beta hydrolase fold)	*
CIMG_06327	1.98	0.00001	1.92	0.00022	conserved hypothetical protein	
CIMG_06333	2.32	4.64E-06	1.87	0.00324	conserved hypothetical protein	
CIMG_06340	1.40	0.03495	1.65	0.04492	RNase3 domain-containing protein	
CIMG_06357	2.04	0.00904	2.41	0.00760	conserved hypothetical protein	
CIMG_06363	1.81	0.00056	2.06	0.00003	hemoglobin and proliferation regulated protein	
CIMG_06364	1.54	0.01566	2.41	9.11E-07	DUF1671 domain-containing protein (peptidase family C78)	
CIMG_06371	2.30	3.90E-08	3.30	1.09E-10	conserved hypothetical protein	
CIMG_06377	1.41	0.03907	1.53	0.01658	FYV10	
CIMG_06378	3.74	7.85E-10	7.54	4.05E-20	UPF0617 protein	
CIMG_06384	3.12	0.00005	2.20	0.02696	conserved hypothetical protein	
CIMG_06390	3.72	0.00003	4.34	0.00002	conserved hypothetical protein	
CIMG_06395	3.00	2.09E-10	4.31	2.43E-12	Ser/Thr protein phosphatase	
CIMG_06399	2.61	3.61E-10	2.07	0.00004	heat shock transcription factor	
CIMG_06404	2.29	0.00031	3.05	2.44E-07	glyceraldehyde-3-phosphate dehydrogenase	
CIMG_06417	4.74	1.77E-22	8.62	3.27E-25	conserved hypothetical protein	
CIMG_06424	1.39	0.04520	2.07	0.00020	D-lactate dehydrogenase 2	
CIMG_06427	1.89	0.00001	4.44	3.32E-18	sulfite oxidase	
CIMG_06434	2.30	0.00182	5.21	4.57E-06	conserved hypothetical protein	*
CIMG_06441	3.26	0.00277	3.68	0.00502	conserved hypothetical protein	*
CIMG_06442	1.60	0.00117	1.42	0.02710	NAD dependent epimerase/dehydratase	
CIMG_06445	4.56	4.98E-20	6.48	4.39E-23	potassium ion transporter	
CIMG_06449	1.57	0.00137	1.92	0.00185	WD domain-containing protein	
CIMG_06478	2.14	8.26E-07	2.21	6.18E-06	amino acid permease	
CIMG_06483	5.09	1.54E-21	2.36	6.88E-07	conserved hypothetical protein	
CIMG_06484	3.78	1.15E-14	2.83	7.86E-09	glyoxylate reductase	
CIMG_06492	3.91	1.56E-06	11.32	1.42E-15	conserved hypothetical protein	
CIMG_06493	5.56	1.07E-08	4.78	1.10E-06	conserved hypothetical protein	
CIMG_06497	2.12	0.00098	2.29	0.00003	conserved hypothetical protein	
CIMG_06500	1.66	0.00144	1.72	0.00246	impact family protein	
CIMG_06513	8.34	9.01E-11	3.94	2.84E-06	conserved hypothetical protein	
CIMG_06517	3.50	1.34E-10	8.57	9.74E-30	conserved hypothetical protein	
CIMG_06519	1.56	0.00098	3.97	1.95E-10	conserved hypothetical protein	
CIMG_06520	6.90	4.14E-16	3.12	2.31E-07	conserved hypothetical protein	
CIMG_06523	1.73	0.00018	2.05	0.00008	conserved hypothetical protein	
CIMG_06528	2.54	5.45E-08	2.14	0.00004	conserved hypothetical protein	*
CIMG_06534	3.28	0.00009	5.48	1.20E-09	conserved hypothetical protein	*
CIMG_06539	2.50	2.12E-09	2.09	0.00006	pyruvate dehydrogenase kinase	
CIMG_06542	2.83	1.36E-08	12.44	2.77E-27	conserved hypothetical protein (Uncharacterized protein family UPF0057)	*
CIMG_06544	1.69	0.00038	1.71	0.02055	Ulp1 protease	
CIMG_06547	2.94	0.00002	4.69	4.21E-10	conserved hypothetical protein (putative aminotransferase)	
CIMG_06552	2.82	2.23E-07	2.95	6.57E-07	conserved hypothetical protein	
CIMG_06559	1.59	0.00457	1.76	0.00274	carboxyvinyl-carboxyphosphonate phosphorylmutase	
CIMG_06577	1.74	0.00012	2.78	4.19E-09	conserved hypothetical protein	
CIMG_06589	1.78	0.00456	1.65	0.01650	conserved hypothetical protein (CHP02453)	

*: Predicted secreted;
+: Gene under positive selection [19]; G: Gene gained in Coccidioides [19]
H, S: Up-regulated in Hyphae or Spherule by microarray [18]

Table S2: All genes up-regulated in parasitic phase

CIMG_06594	3.29	1.39E-15	7.23	8.56E-23	oxidoreductase	
CIMG_06596	1.42	0.01083	2.04	0.00073	alkaline phosphatase	
CIMG_06600	1.65	0.00057	1.49	0.01606	conserved hypothetical protein (mitochondrial fusion and transporter Ugo1)	
CIMG_06609	2.83	1.84E-10	12.36	4.57E-29	cupin 2 domain-containing protein	
CIMG_06612	1.58	0.00750	2.48	1.46E-06	ankyrin repeat protein	
CIMG_06617	1.41	0.01159	3.90	4.17E-16	succinate dehydrogenase cytochrome b560 subunit	
CIMG_06638	1.85	0.00002	1.71	0.00411	conserved hypothetical protein (Nin one binding (NOB1) Zn-ribbon like)	
CIMG_06642	1.43	0.02998	1.79	0.00088	conserved hypothetical protein	**G** *
CIMG_06643	2.40	7.00E-09	1.89	0.00098	scramblase	
CIMG_06645	2.17	0.00248	2.35	0.00350	meiotic recombination protein spo11	
CIMG_06646	1.83	0.00002	1.51	0.02120	oxidosqualene:lanosterol cyclase	
CIMG_06648	2.29	2.15E-08	1.93	0.02394	PQ loop repeat protein	
CIMG_06651	1.52	0.00856	2.24	0.00001	tRNA methyltransferase complex GCD14 subunit	
CIMG_06653	4.26	1.30E-10	1.94	0.00324	conserved hypothetical protein	
CIMG_06659	3.08	2.71E-11	2.18	0.00024	MFS multidrug transporter	
CIMG_06669	2.02	4.06E-06	3.36	1.07E-09	phospholipase/Carboxylesterase superfamily	
CIMG_06683	2.59	8.29E-08	10.84	2.66E-22	DUF1479 domain-containing protein	
CIMG_06687	3.19	0.00001	3.47	0.00057	conserved hypothetical protein	
CIMG_06688	2.16	6.72E-07	3.18	7.05E-11	ubiquitin conjugating enzyme	
CIMG_06694	2.03	0.00190	2.43	0.00005	DEAD box helicase	
CIMG_06705	4.37	3.99E-18	9.62	1.95E-23	conserved hypothetical protein	
CIMG_06707	2.94	8.98E-08	1.82	0.02035	MFS transporter	
CIMG_06720	2.59	8.02E-10	3.76	2.29E-12	triglyceride lipase-cholesterol esterase	* **S**
CIMG_06721	2.70	2.55E-07	1.72	0.01571	conserved hypothetical protein	
CIMG_06725	1.33	0.04616	2.12	0.00036	conserved hypothetical protein	
CIMG_06728	1.95	0.00042	2.04	0.01555	conserved hypothetical protein (Ankyrin repeats, F-box domain)	
CIMG_06765	4.28	0.00021	9.90	6.80E-12	conserved hypothetical protein	
CIMG_06768	1.74	0.00117	4.60	2.20E-12	conserved hypothetical protein	
CIMG_06770	2.96	2.30E-12	2.18	0.00001	phosphoinositide 3-kinase	
CIMG_06773	3.98	5.85E-08	2.17	0.00917	conserved hypothetical protein	**G**
CIMG_06775	1.60	0.00100	1.68	0.01338	serine/threonine-protein kinase PRKX	
CIMG_06787	4.34	8.04E-16	2.71	3.13E-07	conserved hypothetical protein (Coiled-coil domain containing protein (DUF2052))	
CIMG_06790	2.00	0.00003	1.96	0.00007	serine/threonine protein kinase	
CIMG_06791	8.37	1.57E-13	3.69	6.23E-06	conserved hypothetical protein	
CIMG_06797	3.05	3.65E-06	6.77	3.23E-14	conserved hypothetical protein	
CIMG_06798	3.34	1.46E-14	3.75	7.06E-13	conserved hypothetical protein	
CIMG_06799	2.20	1.87E-06	2.80	1.19E-09	high osmolarity signaling protein Sho1	
CIMG_06816	3.23	0.00036	2.46	0.01529	conserved hypothetical protein	
CIMG_06830	1.57	0.02112	2.65	6.65E-06	short chain dehydrogenase	
CIMG_06836	1.56	0.00694	2.70	9.55E-07	isocitrate dehydrogenase	
CIMG_06849	1.76	0.01309	1.35	0.02908	conserved hypothetical protein (Fungal Zn(2)-Cys(6) binuclear cluster domain)	
CIMG_06853	1.99	0.00258	9.49	1.97E-11	conserved hypothetical protein	
CIMG_06858	1.68	0.00043	5.29	5.33E-18	mannosylphosphate transferase	*
CIMG_06865	1.64	0.01386	2.68	5.89E-07	conserved hypothetical protein (FAD dependent oxidoreductase)	
CIMG_06866	7.33	1.07E-06	4.68	7.76E-07	conserved hypothetical protein	
CIMG_06867	2.15	0.00027	4.86	1.35E-12	conserved hypothetical protein (Major facilitator superfamily)	
CIMG_06877	6.65	1.68E-09	3.82	2.48E-08	conserved hypothetical protein	
CIMG_06881	9.93	4.57E-17	3.30	4.20E-09	MFS transporter	
CIMG_06882	7.76	9.00E-08	12.43	1.33E-14	conserved hypothetical protein	
CIMG_06898	2.63	0.00001	2.86	0.00004	conserved hypothetical protein	
CIMG_06907	1.93	0.00008	2.37	0.00007	conserved hypothetical protein	
CIMG_06911	3.10	1.01E-13	4.55	1.26E-16	lanthionine synthetase C family protein	
CIMG_06959	2.56	5.76E-10	3.65	6.73E-13	conserved hypothetical protein	
CIMG_06964	1.62	0.01133	2.60	2.10E-06	sterol desaturase family	*
CIMG_06969	1.97	0.00008	2.31	0.00003	heat shock protein 78	+ **S**
CIMG_06976	3.90	5.34E-11	1.97	0.00902	conserved hypothetical protein	
CIMG_06980	2.42	0.00336	4.82	0.00002	conserved hypothetical protein	
CIMG_06984	6.27	2.19E-11	4.55	2.51E-12	conserved hypothetical protein	
CIMG_06996	3.96	3.30E-06	8.30	8.10E-07	conserved hypothetical protein	
CIMG_07013	4.45	5.22E-20	3.35	7.21E-10	transmembrane amino acid transporter	
CIMG_07014	1.83	0.03030	1.62	0.03987	conserved hypothetical protein	
CIMG_07020	3.17	0.00009	3.90	0.00047	conserved hypothetical protein	
CIMG_07031	2.19	1.16E-08	7.21	9.89E-27	glutamyl-tRNA synthetase	
CIMG_07032	2.58	5.12E-09	7.99	3.84E-17	calcium transporter	
CIMG_07033	1.55	0.00413	2.05	0.00001	conserved hypothetical protein (LSM domain)	
CIMG_07034	2.74	3.11E-11	2.31	9.31E-07	conserved hypothetical protein	
CIMG_07036	1.70	0.00055	4.03	1.24E-10	cytoplasm protein (asparagine synthase)	
CIMG_07062	3.68	2.59E-12	2.94	0.00025	conserved hypothetical protein	
CIMG_07072	2.16	0.00002	2.40	0.00005	conserved hypothetical protein	
CIMG_07073	1.60	0.00134	2.38	1.55E-09	alkaline dihydroceramidase Ydc1	
CIMG_07082	6.88	8.50E-22	5.69	1.99E-19	citrinin biosynthesis oxydoreductase CtnB	
CIMG_07083	4.09	8.38E-09	2.43	0.00009	conserved hypothetical protein (Major Facilitator Superfamily)	
CIMG_07085	5.26	1.28E-12	5.03	1.68E-08	citrinin biosynthesis oxygenase CtnA	
CIMG_07086	5.99	3.46E-15	2.13	0.01524	conserved hypothetical protein (Fungal specific transcription factor domain, fungal Zn(2)-Cys(6) binuclear cluster domain)	
CIMG_07088	4.31	8.48E-07	9.97	3.39E-25	zinc-binding oxidoreductase CipB	
CIMG_07089	12.09	9.74E-12	48.44	2.60E-48	conserved hypothetical protein (YCII-related domain)	
CIMG_07091	1.55	0.00361	1.49	0.01341	LIM domain-containing protein	

*: Predicted secreted;
+: Gene under positive selection [19]; G: Gene gained in Coccididioides [19]
H, S: Up-regulated in Hyphae or Spherule by microarray [18]

Table S2: All genes up-regulated in parasitic phase

CIMG_07094	1.64	0.00688	2.08	0.00102	nitrilase	
CIMG_07101	1.50	0.00699	2.43	4.96E-06	protein kinase	
CIMG_07102	2.30	1.98E-07	1.64	0.00802	3-oxoacyl-(acyl-carrier-protein) reductase	
CIMG_07137	1.56	0.00228	2.17	2.98E-06	cholinephosphate cytidylyltransferase	
CIMG_07140	2.29	3.08E-08	2.48	7.43E-07	acetyltransferase (GNAT family)	
CIMG_07153	2.60	6.87E-11	16.99	6.60E-52	conserved hypothetical protein	
CIMG_07162	6.70	0.00001	4.41	0.00191	conserved hypothetical protein	
CIMG_07166	1.61	0.00131	4.34	4.20E-15	conserved hypothetical protein (putative patatin-like phospholipase)	
CIMG_07167	1.36	0.02508	1.52	0.01563	conserved hypothetical protein (RXT2-like N-terminal domain)	
CIMG_07176	1.35	0.04692	1.64	0.00138	UPF0160 domain-containing protein MYG1	
CIMG_07177	1.76	0.00022	1.55	0.01063	conserved hypothetical protein (putative ER membrane protein ICE2)	*
CIMG_07180	1.78	0.00017	3.56	8.15E-14	DUF431 domain-containing protein (predicted SAM-dependent RNA methyltransferase)	
CIMG_07182	1.64	0.00148	1.58	0.01039	WD repeat protein	
CIMG_07184	5.46	9.97E-11	2.11	0.01407	conserved hypothetical protein	
CIMG_07189	3.25	3.72E-07	18.52	2.25E-24	conserved hypothetical protein	
CIMG_07201	2.31	0.00005	3.90	1.06E-10	conserved hypothetical protein	
CIMG_07203	1.47	0.00338	1.49	0.02898	glycerol-3-phosphate dehydrogenase	
CIMG_07216	1.96	0.00001	6.28	2.94E-23	salicylate hydroxylase	*
CIMG_07237	1.77	0.00033	3.20	9.48E-09	conserved hypothetical protein	
CIMG_07255	1.67	0.00043	1.59	0.02887	conserved hypothetical protein	
CIMG_07256	2.08	0.00001	2.34	0.00003	adoMet-dependent tRNA methyltransferase complex subunit Trm112	
CIMG_07263	2.08	0.00005	2.59	2.84E-06	isocitrate dehydrogenase subunit 1	
CIMG_07265	1.81	0.00004	1.63	0.00566	methyltransferase	
CIMG_07270	1.72	0.00019	1.93	0.00057	conserved hypothetical protein (HCNGP-like protein)	
CIMG_07281	5.31	2.98E-12	2.97	0.00002	conserved hypothetical protein	
CIMG_07287	1.65	0.00029	2.01	2.79E-06	DnaJ chaperone	
CIMG_07295	2.28	2.85E-07	7.78	1.11E-26	conserved hypothetical protein	
CIMG_07309	3.09	0.00001	2.00	0.01513	conserved hypothetical protein	
CIMG_07324	1.63	0.00240	1.58	0.01315	26S protease regulatory subunit S10B	
CIMG_07329	2.01	3.01E-06	1.59	0.00855	telomere-binding alpha subunit central domain-containing protein	
CIMG_07333	1.51	0.00353	2.19	1.72E-06	sulphite efflux pump protein	
CIMG_07338	9.99	1.58E-30	10.30	2.43E-35	essential protein Yae1	
CIMG_07339	10.95	3.05E-32	6.14	4.11E-27	conserved hypothetical protein (Putative phosphatase regulatory subunit)	
CIMG_07354	2.07	0.00003	1.64	0.02224	RNA polymerase II transcription factor B subunit 5	
CIMG_07462	1.81	0.00004	2.12	0.00005	diacylglycerol O-acyltransferase	
CIMG_07464	1.33	0.04735	1.99	0.00011	conserved hypothetical protein (G-patch domain)	
CIMG_07514	3.11	0.00001	2.67	0.00209	conserved hypothetical protein	
CIMG_07525	1.88	0.00005	2.93	2.22E-10	amino acid permease	
CIMG_07529	1.71	0.00088	1.80	0.00118	conserved hypothetical protein	*
CIMG_07537	7.53	1.32E-29	1.62	0.00189	conserved hypothetical protein	
CIMG_07542	1.58	0.00220	2.03	0.00009	type II protein geranylgeranyltransferase, beta subunit	
CIMG_07543	3.94	2.32E-08	3.27	0.00005	conserved hypothetical protein	
CIMG_07565	1.65	0.00110	2.28	6.59E-06	BEM46 family protein	
CIMG_07572	3.71	9.28E-10	3.97	1.33E-10	conserved hypothetical protein (Fungal Zn(2)-Cys(6) binuclear cluster domain)	
CIMG_07600	1.46	0.01923	1.76	0.00054	NUDIX domain-containing protein	
CIMG_07608	2.90	2.88E-10	2.95	1.80E-07	conserved hypothetical protein	
CIMG_07610	4.21	0.00014	1.99	0.03243	conserved hypothetical protein	
CIMG_07632	3.35	3.71E-06	1.83	0.00625	conserved hypothetical protein	
CIMG_07636	5.10	5.33E-23	5.25	1.38E-17	conserved hypothetical protein	
CIMG_07640	3.55	6.84E-14	3.27	3.15E-10	conserved hypothetical protein (Phosphotransferase enzyme family)	
CIMG_07644	1.83	0.01653	6.80	2.63E-09	conserved hypothetical protein	
CIMG_07645	7.33	1.05E-27	2.19	0.00004	protein kinase domain-containing protein	
CIMG_07646	24.59	1.19E-53	7.49	1.79E-21	conserved hypothetical protein	
CIMG_07647	7.28	5.08E-21	2.55	9.80E-07	conserved hypothetical protein	
CIMG_07651	2.13	0.00006	1.84	0.00581	conserved hypothetical protein	
CIMG_07661	2.09	1.15E-06	1.54	0.02033	glucose-6-phosphate 1-dehydrogenase	
CIMG_07665	4.07	9.01E-11	3.04	2.69E-06	mitochondrial phosphate carrier protein 2	
CIMG_07666	1.90	0.00421	5.07	3.06E-12	conserved hypothetical protein	**G**
CIMG_07675	3.01	5.02E-12	1.92	0.00009	SH3 domain-containing protein	
CIMG_07676	1.71	0.00330	1.91	0.00070	conserved hypothetical protein	*
CIMG_07685	3.37	1.24E-07	21.69	2.99E-29	conserved hypothetical protein	
CIMG_07692	1.52	0.00706	2.93	1.04E-08	NADH-ubiquinone oxidoreductase subunit GRIM-19	
CIMG_07700	2.67	2.98E-10	5.21	6.21E-17	conserved hypothetical protein	
CIMG_07701	1.44	0.00481	5.00	1.31E-13	conserved hypothetical protein	
CIMG_07702	2.48	0.00019	2.37	0.00002	conserved hypothetical protein	*
CIMG_07706	2.82	1.77E-07	1.76	0.04096	conserved hypothetical protein (F-box domain)	
CIMG_07711	3.61	1.04E-14	1.83	0.00209	conserved hypothetical protein	
CIMG_07721	5.80	9.73E-07	2.52	0.01157	conserved hypothetical protein	
CIMG_07723	1.49	0.04659	2.69	1.53E-06	polysaccharide synthase Cps1	
CIMG_07724	3.20	0.00002	2.59	0.00092	conserved hypothetical protein	
CIMG_07734	1.49	0.00788	1.54	0.03695	helicase swr1	
CIMG_07743	2.09	0.00029	2.68	0.00073	conserved hypothetical protein	
CIMG_07753	3.91	2.00E-16	5.16	1.03E-17	conserved hypothetical protein	
CIMG_07755	2.87	1.54E-09	3.62	4.19E-10	conserved hypothetical protein	**G**
CIMG_07757	1.69	0.00022	2.22	4.86E-06	conserved hypothetical protein	
CIMG_07766	4.13	6.47E-14	3.54	4.27E-10	conserved hypothetical protein (Putative methyltransferase)	
CIMG_07798	1.80	0.00020	3.92	4.98E-14	conserved hypothetical protein (Fungal protein of unknown function (DUF1711))	

*: Predicted secreted;
+: Gene under positive selection [19]; G: Gene gained in Coccididioides [19]
H, S: Up-regulated in Hyphae or Spherule by microarray [18]

Table S2: All genes up-regulated in parasitic phase

CIMG_07803	4.77	1.38E-19	1.54	0.03147	indoleamine 2,3-dioxygenase	
CIMG_07804	1.93	0.01097	2.50	0.00040	conserved hypothetical protein (tetratricopeptide repeat)	
CIMG_07806	2.04	0.00229	2.03	0.00295	conserved hypothetical protein	
CIMG_07807	4.33	2.77E-06	9.57	1.13E-12	conserved hypothetical protein	
CIMG_07815	3.16	6.17E-09	11.24	9.29E-24	short chain dehydrogenase/reductase	
CIMG_07822	3.47	0.00122	4.14	0.00065	conserved hypothetical protein	
CIMG_07829	8.63	2.24E-22	2.04	0.00207	conserved hypothetical protein (Fungal Zn(2)-Cys(6) binuclear cluster domain)	
CIMG_07834	1.62	0.03938	2.63	1.70E-06	conserved hypothetical protein (UbiD family decarboxylases)	
CIMG_07840	4.67	0.00001	4.69	4.27E-06	conserved hypothetical protein	
CIMG_07842	3.52	2.49E-13	1.81	0.00181	conserved hypothetical protein (WSC domain, putative peroxidase)	*
CIMG_07843	3.40	3.40E-11	1.65	0.00028	prp 6 CRoW domain-containing protein	*
CIMG_07849	1.70	0.00014	8.13	8.23E-29	conserved hypothetical protein (DUF2263)	
CIMG_07853	2.47	0.02317	3.71	0.00818	conserved hypothetical protein	
CIMG_07857	1.81	0.00016	2.09	0.00003	hypothetical protein (CHCH domain)	**G**
CIMG_07867	1.41	0.02013	2.30	2.39E-06	SNARE protein	
CIMG_07895	2.09	1.05E-06	1.98	0.00002	conserved hypothetical protein	
CIMG_07902	1.75	0.00134	1.90	0.00108	DNA repair protein rad9	
CIMG_07903	2.01	4.50E-06	1.74	0.00006	alpha-tubulin suppressor protein Aats1	
CIMG_07904	5.26	1.97E-12	3.62	1.86E-09	conserved hypothetical protein	
CIMG_07921	5.08	0.00001	3.92	1.78E-07	conserved hypothetical protein	
CIMG_07930	3.66	9.06E-16	2.38	1.75E-06	conserved hypothetical protein	
CIMG_07948	3.92	3.15E-08	4.23	2.36E-08	conserved hypothetical protein	
CIMG_07949	2.06	0.00006	3.42	6.25E-09	conserved hypothetical protein (NmrA-like family)	
CIMG_07959	1.71	0.01924	1.60	0.04656	conserved hypothetical protein (putative ADP-ribosylglycohydrolase)	
CIMG_07965	17.39	1.62E-11	5.79	1.50E-09	conserved hypothetical protein	
CIMG_07966	4.78	1.11E-06	2.28	0.01268	conserved hypothetical protein	
CIMG_07968	2.44	0.00021	2.53	0.00051	conserved hypothetical protein	
CIMG_07972	2.42	1.27E-06	3.34	1.69E-09	developmental regulator flbA	**S**
CIMG_07982	1.49	0.03133	1.59	0.02747	DNA replication complex GINS protein PSF2	
CIMG_07983	4.89	1.28E-08	3.57	0.00007	conserved hypothetical protein	
CIMG_07989	3.36	1.55E-14	3.65	1.84E-12	conserved hypothetical protein	*
CIMG_07997	1.90	0.00002	3.06	4.41E-11	maleylacetate reductase	
CIMG_07999	2.43	7.51E-09	2.37	0.00001	conserved hypothetical protein	
CIMG_08005	3.02	5.48E-07	2.89	3.15E-06	conserved hypothetical protein	
CIMG_08006	3.13	0.00086	1.94	0.01279	conserved hypothetical protein	
CIMG_08014	1.79	0.00004	2.42	0.00004	conserved hypothetical protein	**G**
CIMG_08017	3.43	1.50E-12	3.71	2.18E-11	nitrate reductase	**S**
CIMG_08021	5.05	1.06E-16	2.51	5.84E-06	kynurenine 3-monooxygenase	
CIMG_08028	2.52	9.41E-10	3.35	1.36E-11	conserved hypothetical protein (DUF1769)	
CIMG_08040	3.20	0.00027	4.92	0.00220	conserved hypothetical protein	
CIMG_08044	3.97	1.23E-08	13.58	2.98E-27	cell wall integrity signaling protein Lsp1/Pil1	
CIMG_08048	2.67	1.12E-10	1.71	0.00142	LSM domain-containing protein	
CIMG_08051	1.68	0.00063	1.84	0.00103	chromatin assembly factor 1 subunit B	
CIMG_08055	2.30	0.00048	7.83	1.55E-18	hypothetical protein	
CIMG_08056	7.96	4.14E-07	2.75	0.00126	conserved hypothetical protein	
CIMG_08072	6.62	2.36E-16	3.93	1.89E-08	conserved hypothetical protein (cytochrome P450)	
CIMG_08077	4.66	7.04E-12	3.05	8.53E-07	conserved hypothetical protein	*
CIMG_08082	5.46	6.18E-23	2.35	1.44E-08	mitochondrial import receptor subunit	
CIMG_08085	3.51	3.45E-15	3.21	9.07E-10	conserved hypothetical protein	
CIMG_08096	1.76	0.00011	2.09	0.00017	conserved hypothetical protein	
CIMG_08103	6.50	1.09E-11	56.16	2.50E-48	opsin 1	
CIMG_08116	1.87	0.00374	2.07	0.00303	conserved hypothetical protein	
CIMG_08119	2.28	6.47E-08	2.36	6.23E-06	DNA-directed polymerase kappa	
CIMG_08122	1.92	0.00566	2.10	0.00357	conserved hypothetical protein	
CIMG_08132	2.62	9.39E-07	1.84	0.00466	conserved hypothetical protein	
CIMG_08150	1.79	0.00192	1.55	0.00022	carbon catabolite repressor	
CIMG_08152	3.69	1.60E-11	3.00	0.00009	conserved hypothetical protein	
CIMG_08153	4.85	0.00002	3.98	0.00006	conserved hypothetical protein	
CIMG_08154	1.97	0.02272	2.31	0.01558	conserved hypothetical protein	
CIMG_08155	1.58	0.00142	1.83	0.00052	MOSC domain-containing protein	
CIMG_08156	3.80	2.02E-16	3.03	1.74E-10	conserved hypothetical protein	
CIMG_08157	3.90	1.42E-12	3.65	6.34E-13	conserved hypothetical protein	
CIMG_08158	6.16	4.86E-08	5.06	9.20E-12	acyl-CoA desaturase	
CIMG_08167	2.64	7.23E-09	2.66	0.00003	5-formyltetrahydrofolate cyclo-ligase	
CIMG_08168	1.83	0.00021	1.66	0.01075	rot1 (chaperone for protein-folding within the ER)	*
CIMG_08180	2.23	2.10E-06	2.99	1.19E-07	conserved hypothetical protein (F-box domain)	
CIMG_08195	1.51	0.00460	1.66	0.01392	acetyltransferase	
CIMG_08201	1.78	0.00057	5.48	2.00E-16	conserved hypothetical protein	
CIMG_08209	1.76	0.00066	8.62	4.81E-22	cytochrome c peroxidase Ccp1	
CIMG_08221	5.16	3.64E-09	2.53	0.00039	high affinity methionine permease	
CIMG_08289	5.86	5.08E-20	5.49	2.14E-08	calcium/calmodulin dependent protein kinase	
CIMG_08294	1.87	0.00002	2.98	3.72E-09	conserved hypothetical protein	
CIMG_08303	1.67	0.00079	2.25	0.00002	conserved hypothetical protein	
CIMG_08310	1.36	0.02155	3.37	1.82E-10	MFS monosaccharide transporter	
CIMG_08323	3.71	1.86E-16	3.05	2.84E-09	conserved hypothetical protein	
CIMG_08328	4.18	5.53E-19	4.21	3.71E-15	conserved hypothetical protein	
CIMG_08337	2.46	2.47E-07	2.08	0.00210	molybdenum cofactor sulfurase	*
CIMG_08342	6.21	4.91E-08	3.37	0.00003	conserved hypothetical protein	
CIMG_08457	2.00	0.00256	1.95	0.00295	conserved hypothetical protein	

*: Predicted secreted;
+: Gene under positive selection [19]; G: Gene gained in Coccidioides [19]
H, S: Up-regulated in Hyphae or Spherule by microarray [18]

Table S2: All genes up-regulated in parasitic phase

CIMG_08458	1.61	0.00139	3.24	3.59E-10	glutamyl-tRNA amidotransferase	
CIMG_08474	8.94	1.55E-29	5.42	2.68E-09	conserved hypothetical protein	
CIMG_08477	2.33	0.00003	1.57	0.00842	fatty acid synthase alpha subunit FasA	
CIMG_08490	1.77	0.01471	4.03	3.20E-07	conserved hypothetical protein	
CIMG_08491	1.79	0.00254	1.40	0.03673	conserved hypothetical protein	
CIMG_08492	2.44	1.01E-07	1.94	0.00102	inositol pyrophosphate synthase	
CIMG_08495	1.70	0.01237	3.10	4.48E-06	conserved hypothetical protein	
CIMG_08504	2.84	2.05E-07	2.24	0.00601	conserved hypothetical protein (PAN domain)	*
CIMG_08509	3.07	6.30E-11	2.58	2.88E-07	conserved hypothetical protein (Pterin 4 alpha carbinolamine dehydratase)	
CIMG_08518	2.45	1.78E-07	2.83	2.69E-07	riboflavin-specific deaminase	
CIMG_08530	4.53	5.37E-10	11.94	6.44E-18	conserved hypothetical protein	
CIMG_08535	1.65	0.00240	1.85	0.00139	ubiquitin-conjugating enzyme E2 6	
CIMG_08537	2.03	0.00001	2.20	0.00003	cytochrome c heme lyase	
CIMG_08540	3.84	2.14E-15	4.10	2.04E-12	ubiquinone biosynthesis protein (ABC1 family)	
CIMG_08545	1.35	0.03383	2.96	1.30E-08	short chain dehydrogenase/reductase	
CIMG_08547	1.56	0.00319	2.07	0.00020	conserved hypothetical protein	
CIMG_08563	2.09	1.08E-06	1.37	0.04164	AflT	
CIMG_08564	2.86	2.54E-11	1.78	0.00175	fatty-acid synthase	
CIMG_08613	2.20	0.00372	4.40	6.21E-08	metalloproteinase 7	*
CIMG_08614	3.17	0.00425	4.46	0.00010	conserved hypothetical protein	
CIMG_08623	1.73	0.00144	2.30	0.00074	actin-like protein arp6	
CIMG_08637	2.71	1.35E-10	13.98	5.77E-36	copper resistance protein Crd2	
CIMG_08653	3.07	3.74E-07	2.78	2.52E-15	conserved hypothetical protein	
CIMG_08660	1.68	0.00068	1.59	0.02754	SAC3/GANP domain-containing protein	
CIMG_08671	4.67	3.48E-12	2.57	0.00161	conserved hypothetical protein	
CIMG_08675	2.30	1.19E-06	2.09	0.00010	conserved hypothetical protein	
CIMG_08679	2.51	1.17E-08	1.93	2.99E-06	conserved hypothetical protein (putative mitochondrial 18 KDa protein (MTP18))	
CIMG_08686	4.00	1.24E-08	4.61	3.21E-12	conserved hypothetical protein	
CIMG_08701	2.85	0.01954	2.61	0.00649	conserved hypothetical protein	
CIMG_08703	1.55	0.00280	2.21	3.99E-06	conserved hypothetical protein (Alkaline phytoceramidase (aPHC))	
CIMG_08709	3.13	0.00007	3.31	0.00009	conserved hypothetical protein	
CIMG_08716	2.10	4.31E-07	3.53	1.49E-11	malic acid transporter	
CIMG_08719	1.45	0.00957	2.19	0.00006	conserved hypothetical protein	
CIMG_08720	4.31	3.56E-10	9.82	2.14E-22	fumarate reductase Osm1	*
CIMG_08724	2.61	0.00001	4.27	5.32E-13	acetyl-coA hydrolase Ach1	
CIMG_08770	4.64	5.39E-21	5.19	1.18E-19	MFS multidrug transporter	
CIMG_08779	2.21	0.00013	4.91	5.54E-16	conserved hypothetical protein (Putative methyltransferase)	
CIMG_08780	2.19	3.33E-07	2.15	0.00031	conserved hypothetical protein	
CIMG_08782	7.55	1.37E-28	6.11	4.87E-19	conserved hypothetical protein	
CIMG_08783	3.16	0.00001	2.04	0.00705	conserved hypothetical protein	
CIMG_08815	1.44	0.01139	2.02	0.00182	threonine dehydratase	
CIMG_08832	2.86	0.00001	3.03	0.00002	conserved hypothetical protein	
CIMG_08867	1.91	0.00005	1.98	0.00323	MFS multidrug transporter	
CIMG_08872	2.97	1.09E-09	2.88	4.27E-09	oxidoreductase (2OG-Fe(II) oxygenase superfamily)	
CIMG_08873	3.35	8.26E-09	5.93	5.22E-17	NmrA family transcriptional regulator	
CIMG_08879	1.53	0.01293	1.69	0.02078	6-phosphofructokinase	
CIMG_08903	2.21	0.00100	2.76	0.00081	conserved hypothetical protein	
CIMG_08913	1.67	0.02646	4.49	3.27E-12	conserved hypothetical protein	
CIMG_08914	1.61	0.04593	2.16	0.00014	conserved hypothetical protein	
CIMG_08919	2.01	1.06E-06	4.17	4.18E-19	conserved hypothetical protein (NmrA-like family)	
CIMG_08979	1.59	0.00221	1.54	0.02547	proteasome regulatory particle subunit	
CIMG_08986	2.76	5.73E-10	4.76	2.94E-14	MFS monocarboxylate transporter	
CIMG_08990	3.07	2.98E-10	3.07	2.90E-08	mitochondrial hypoxia responsive domain-containing protein	
CIMG_08993	2.54	2.11E-06	5.52	3.20E-22	MFS multidrug transporter	
CIMG_09001	3.75	6.61E-08	6.31	1.34E-17	conserved hypothetical protein	*
CIMG_09013	1.83	0.00006	1.75	0.00029	conserved hypothetical protein	
CIMG_09020	3.92	5.48E-17	2.41	2.78E-06	conserved hypothetical protein	
CIMG_09040	2.00	1.18E-06	2.62	1.27E-08	conserved hypothetical protein	
CIMG_09045	2.69	2.98E-10	3.80	2.15E-11	acyl-CoA thioesterase	
CIMG_09049	3.86	6.08E-11	4.65	1.35E-11	conserved hypothetical protein	
CIMG_09058	10.28	6.62E-17	14.16	1.87E-17	conserved hypothetical protein	
CIMG_09059	2.40	2.56E-09	4.36	1.72E-14	serine palmitoyltransferase 2	
CIMG_09061	1.68	0.00030	4.43	8.94E-21	acetoacetyl-CoA synthase	
CIMG_09062	2.35	2.30E-06	1.54	0.00462	mitochondrial processing peptidase alpha subunit	
CIMG_09079	2.52	4.97E-09	1.75	0.01801	cation diffusion facilitator 1	
CIMG_09087	4.60	1.04E-09	2.65	0.00008	conserved hypothetical protein (Fungal Zn(2)-Cys(6) binuclear cluster domain)	
CIMG_09095	4.71	1.58E-12	4.84	2.27E-08	conserved hypothetical protein	
CIMG_09097	2.47	4.12E-08	3.59	1.04E-10	conserved hypothetical protein	
CIMG_09099	1.44	0.01665	2.06	0.00004	HAD superfamily hydrolase	
CIMG_09105	5.55	3.88E-08	3.90	1.65E-06	conserved hypothetical protein	
CIMG_09112	1.33	0.04592	1.79	0.00243	TPR repeat protein	
CIMG_09117	2.29	0.00016	5.06	1.12E-06	conserved hypothetical protein	
CIMG_09121	1.90	0.00001	3.29	2.61E-10	conserved hypothetical protein	
CIMG_09140	1.96	0.00001	1.59	0.00635	conserved hypothetical protein	
CIMG_09146	1.38	0.04114	6.82	2.33E-22	quinone oxidoreductase	
CIMG_09152	2.77	2.43E-06	3.57	1.36E-07	conserved hypothetical protein (Histidine acid phosphatase)	*
CIMG_09153	4.23	3.56E-18	1.60	0.00940	conserved hypothetical protein	
CIMG_09155	1.78	0.00013	1.56	0.00917	serine/threonine-protein kinase nrc-2	

*: Predicted secreted;
+: Gene under positive selection [19]; G: Gene gained in Coccidioides [19]
H, S: Up-regulated in Hyphae or Spherule by microarray [18]

Table S2: All genes up-regulated in parasitic phase

CIMG_09160	1.79	0.00057	1.78	0.01962	transcription factor TFIIH, subunit Tfb4	
CIMG_09181	3.05	9.76E-10	2.07	8.68E-06	TOR signaling pathway regulator	
CIMG_09188	1.53	0.00806	1.45	0.01786	survival factor 1	
CIMG_09191	1.97	0.00001	1.62	0.00127	protein kinase	**S**
CIMG_09194	2.18	0.00001	3.13	1.38E-08	mitochondrial carrier protein	
CIMG_09205	7.27	8.86E-12	9.96	1.22E-19	conserved hypothetical protein	
CIMG_09211	1.67	0.00019	1.92	0.00064	conserved hypothetical protein	*
CIMG_09213	2.56	2.09E-09	1.89	0.00270	nitrogen assimilation transcription factor nirA	**H**
CIMG_09214	4.06	7.89E-07	4.17	8.71E-08	conserved hypothetical protein	
CIMG_09226	1.58	0.01360	1.67	0.00533	conserved hypothetical protein (PH domain)	
CIMG_09238	6.15	1.58E-15	18.64	2.35E-28	conserved hypothetical protein	
CIMG_09260	2.32	0.00007	1.81	0.00383	conserved hypothetical protein	
CIMG_09276	1.53	0.00603	3.22	9.57E-10	mitochondrial inner membrane protease subunit 1	
CIMG_09286	5.27	8.24E-14	4.92	4.05E-10	conserved hypothetical protein	
CIMG_09309	2.06	0.00006	2.25	3.59E-06	UPF0052 domain-containing protein	
CIMG_09313	3.23	8.82E-13	1.97	0.00058	CorA family metal ion transporter	
CIMG_09317	4.04	2.02E-18	8.27	2.88E-30	conserved hypothetical protein	
CIMG_09319	1.40	0.02825	1.96	0.00072	conserved hypothetical protein (pentatricopeptide repeat domain)	
CIMG_09324	1.61	0.00190	1.59	0.03653	cell cycle control protein	
CIMG_09325	1.60	0.00145	2.96	5.76E-09	aminomethyl transferase	
CIMG_09327	2.49	1.67E-09	2.79	3.69E-09	conserved hypothetical protein	
CIMG_09332	3.18	0.00001	1.70	0.01454	conserved hypothetical protein (DUF2392)	
CIMG_09336	4.14	1.30E-14	29.61	1.57E-47	conserved hypothetical protein	
CIMG_09338	28.41	3.75E-14	15.49	1.96E-12	conserved hypothetical protein	
CIMG_09340	3.55	1.46E-07	2.52	0.00021	C-5 cytosine methyltransferase DmtA	
CIMG_09341	3.38	1.04E-07	6.63	2.85E-14	conserved hypothetical protein	
CIMG_09344	1.85	0.00010	1.93	0.00054	WD repeat protein	
CIMG_09345	3.82	1.06E-13	1.91	0.00036	mitochondrial oxaloacetate transporter	
CIMG_09353	1.97	0.00001	2.07	0.00003	ribosome biogenesis protein Ria1	
CIMG_09354	2.40	2.67E-07	2.21	0.00002	conserved hypothetical protein	
CIMG_09404	1.77	0.00017	1.48	0.00558	conserved hypothetical protein	
CIMG_09405	3.82	2.24E-16	2.50	1.07E-06	conserved hypothetical protein	
CIMG_09408	1.39	0.03709	2.01	0.00045	conserved hypothetical protein	
CIMG_09424	1.38	0.03041	2.62	4.02E-07	50S ribosomal subunit L30	
CIMG_09431	2.36	0.03277	5.35	1.76E-06	conserved hypothetical protein	
CIMG_09439	6.39	3.91E-27	3.43	3.95E-13	conserved hypothetical protein	
CIMG_09440	3.05	2.17E-08	5.04	7.46E-13	conserved hypothetical protein (PHD-finger)	
CIMG_09459	1.58	0.00574	1.81	0.00072	conserved hypothetical protein (Protein of unknown function (DUF2454))	
CIMG_09471	8.07	9.41E-26	19.28	1.73E-32	tyrosinase central domain-containing protein	*
CIMG_09477	2.39	1.67E-09	3.12	7.61E-11	cyanate hydratase	
CIMG_09478	4.17	2.08E-16	25.83	5.34E-48	3-dehydroshikimate dehydratase	
CIMG_09483	4.78	9.80E-18	7.65	1.29E-26	conserved hypothetical protein	
CIMG_09484	5.29	1.27E-14	4.16	4.89E-13	conserved hypothetical protein	
CIMG_09485	4.64	7.54E-21	4.28	1.46E-18	conserved hypothetical protein (Fungal protein of unknown function (DUF1760))	
CIMG_09486	3.50	8.98E-14	5.80	7.31E-20	conserved hypothetical protein	
CIMG_09494	2.44	2.06E-08	3.18	4.26E-11	conserved hypothetical protein	
CIMG_09498	3.16	1.13E-13	9.73	1.44E-39	conserved hypothetical protein	*
CIMG_09499	1.47	0.01213	1.51	0.01139	conserved hypothetical protein (B-block binding subunit of TFIIIC)	
CIMG_09500	2.19	0.01091	5.29	1.34E-06	conserved hypothetical protein	*
CIMG_09502	1.62	0.00120	1.81	0.00017	glutamyl-tRNA synthetase	
CIMG_09521	3.25	2.99E-12	1.56	0.02528	serine peptidase, family S28	*
CIMG_09522	2.02	0.00005	1.59	0.03219	FAD binding domain-containing protein	
CIMG_09523	1.31	0.04692	2.35	7.52E-07	conserved hypothetical protein	*
CIMG_09539	64.98	4.20E-26	184.24	8.57E-61	conserved hypothetical protein	
CIMG_09540	2.01	0.00059	5.74	1.57E-19	conserved hypothetical protein	
CIMG_09541	2.91	0.02495	7.35	3.00E-09	conserved hypothetical protein	
CIMG_09568	1.59	0.00159	1.69	0.01011	INO80 chromatin remodeling complex	
CIMG_09581	3.33	1.15E-07	4.33	1.57E-09	conserved hypothetical protein	
CIMG_09583	1.54	0.03115	1.83	0.01183	conserved hypothetical protein	
CIMG_09599	2.55	0.00654	4.24	0.00038	conserved hypothetical protein	
CIMG_09609	2.69	4.18E-07	6.90	7.38E-22	conserved hypothetical protein	
CIMG_09614	2.10	0.01109	6.10	7.76E-13	hypothetical protein	
CIMG_09620	2.47	2.44E-07	1.66	0.00936	recombination hotspot-binding protein	
CIMG_09641	2.50	2.58E-06	1.97	0.00074	conserved hypothetical protein	
CIMG_09642	13.43	2.07E-34	4.19	6.15E-09	conserved hypothetical protein	
CIMG_09668	2.48	1.74E-07	2.87	4.98E-08	conserved hypothetical protein (WD repeat-containing protein 79)	
CIMG_09695	4.74	1.90E-12	6.17	1.29E-12	conserved hypothetical protein	
CIMG_09698	3.90	2.65E-07	12.16	7.05E-11	conserved hypothetical protein	
CIMG_09707	1.61	0.00301	1.57	0.00220	transcriptional elongation regulator Elc1/Elongin C	
CIMG_09714	4.36	2.28E-08	2.92	0.00004	conserved hypothetical protein (RNAse P Rpr2/Rpp21/SNM1 subunit domain)	
CIMG_09743	2.64	0.00029	1.99	0.00197	conserved hypothetical protein	*
CIMG_09749	4.14	2.35E-12	35.56	4.23E-53	conserved hypothetical protein	
CIMG_09750	3.94	2.82E-08	2.01	0.03637	cyclic peptide synthetase	**S**
CIMG_09751	22.88	4.40E-27	11.18	6.01E-15	conserved hypothetical protein	
CIMG_09753	6.57	2.90E-09	2.69	0.00121	ABC multidrug transporter	
CIMG_09764	2.21	4.16E-06	3.06	2.00E-10	ABC transporter	
CIMG_09765	12.92	4.62E-39	9.18	2.01E-28	oxidoreductase	
CIMG_09793	1.78	0.00065	1.80	0.00123	vesicle-associated membrane protein 712	

*: Predicted secreted;
+: Gene under positive selection [19]; G: Gene gained in Coccidioides [19]
H, S: Up-regulated in Hyphae or Spherule by microarray [18]

Table S2: All genes up-regulated in parasitic phase

CIMG_09795	6.63	6.73E-09	4.33	9.93E-07	conserved hypothetical protein	
CIMG_09796	2.12	1.19E-06	2.57	8.92E-09	ura1	
CIMG_09798	1.68	0.00080	2.33	0.00001	conserved hypothetical protein	
CIMG_09830	2.98	3.81E-11	2.85	1.57E-07	conserved hypothetical protein	
CIMG_09894	5.38	3.91E-14	6.90	1.03E-16	cytochrome P450	
CIMG_09897	3.32	1.51E-14	2.35	3.26E-06	conserved hypothetical protein (Thioesterase domain)	
CIMG_09902	2.30	1.57E-07	4.11	1.23E-16	conserved hypothetical protein (Phosphotransferase enzyme family)	
CIMG_09940	2.72	6.66E-09	2.28	0.00013	conserved hypothetical protein	**+**
CIMG_09960	1.66	0.00053	1.54	0.00508	conserved hypothetical protein	
CIMG_09972	5.99	4.36E-20	7.19	9.58E-18	DNA mismatch repair protein Msh4	
CIMG_09973	1.60	0.00056	1.80	0.00020	WD repeat protein	
CIMG_09978	2.80	5.21E-10	1.72	0.00802	conserved hypothetical protein	
CIMG_09980	2.93	3.75E-10	1.92	0.00214	DUF221 domain-containing protein	
CIMG_09990	10.56	9.39E-09	6.75	8.88E-08	conserved hypothetical protein	
CIMG_09991	1.98	0.01436	6.97	4.19E-10	conserved hypothetical protein	
CIMG_10000	8.35	1.44E-24	18.78	5.86E-31	conserved hypothetical protein	
CIMG_10001	5.23	3.19E-08	2.45	0.01859	conserved hypothetical protein	
CIMG_10002	2.79	5.84E-10	11.35	1.22E-33	conserved hypothetical protein (F-box domain)	
CIMG_10013	10.25	3.10E-35	1.57	0.04276	conserved hypothetical protein	
CIMG_10015	6.06	2.18E-06	6.97	1.92E-11	conserved hypothetical protein	
CIMG_10020	2.66	1.30E-10	5.40	1.18E-18	glutamate decarboxylase	
CIMG_10022	2.81	8.98E-08	13.12	3.60E-30	conserved hypothetical protein	
CIMG_10023	9.67	8.13E-15	6.19	1.67E-12	conserved hypothetical protein	
CIMG_10026	2.08	3.50E-06	4.77	3.17E-15	conserved hypothetical protein (DUF1687)	
CIMG_10027	2.39	1.04E-06	2.06	0.00009	conserved hypothetical protein	
CIMG_10030	1.81	0.00103	1.74	0.01160	fungal specific transcription factor domain-containing protein	
CIMG_10039	1.50	0.01532	4.63	6.60E-14	short-chain dehydrogenase/reductase	
CIMG_10046	3.21	0.00143	3.54	0.00002	hypothetical protein	
CIMG_10050	2.11	0.00001	2.40	0.00003	ankyrin repeat and BTB/POZ domain-containing protein	
CIMG_10054	2.10	0.00001	1.70	0.00390	conserved hypothetical protein	
CIMG_10055	2.79	6.58E-12	3.56	5.34E-11	RNAse P Rpr2/Rpp21 subunit domain-containing protein	
CIMG_10064	26.71	3.10E-54	3.93	1.43E-06	conserved hypothetical protein	
CIMG_10086	2.54	1.01E-09	1.70	0.01505	chitin synthase activator	
CIMG_10094	5.05	1.15E-10	3.64	1.77E-06	conserved hypothetical protein	
CIMG_10103	2.96	0.00011	1.87	0.03691	serine/threonine protein kinase	
CIMG_10109	3.23	9.19E-13	1.55	0.03924	alcohol dehydrogenase	**S**
CIMG_10112	2.70	1.22E-07	3.74	1.36E-10	aromatic ring-opening dioxygenase LigB subunit	
CIMG_10124	1.82	0.00063	2.05	0.00090	RTA1 domain-containing protein	
CIMG_10133	3.94	1.73E-14	3.15	3.83E-08	LCCL domain-containing protein	
CIMG_10144	1.84	0.00019	2.37	3.77E-06	oxidoreductase (Aldehyde dehydrogenase family)	
CIMG_10146	3.13	4.71E-10	5.14	9.89E-15	conserved hypothetical protein	
CIMG_10163	2.25	2.48E-06	2.54	0.00002	L-aminoadipate-semialdehyde dehydrogenase	
CIMG_10166	5.60	5.04E-18	5.68	3.78E-21	pyoverdine/dityrosine biosynthesis family protein	
CIMG_10169	3.05	1.77E-12	1.62	0.01221	cell cycle checkpoint protein (Hus1-like protein)	
CIMG_10172	1.44	0.02524	1.50	0.03559	conserved hypothetical protein	
CIMG_10173	2.39	1.27E-06	2.11	0.00032	conserved hypothetical protein (putative autophagocytosis associated protein)	
CIMG_10180	2.20	9.91E-07	1.61	0.00625	pheromone-dependent cell cycle arrest protein Far11	
CIMG_10182	7.40	3.08E-30	2.05	0.00010	conserved hypothetical protein	**G**
CIMG_10185	2.85	7.37E-12	3.69	7.50E-12	conserved hypothetical protein	
CIMG_10189	2.76	0.00115	3.60	0.00002	conserved hypothetical protein	
CIMG_10204	2.26	7.97E-08	2.67	5.92E-08	DUF28 domain-containing protein	
CIMG_10236	1.76	0.00022	1.53	0.01659	mismatch-specific thymine-DNA glycosylase	
CIMG_10239	3.18	3.14E-11	2.13	0.00010	NADPH oxidase	
CIMG_10240	2.98	0.00001	7.87	8.43E-15	RNA binding protein	
CIMG_10241	3.13	7.07E-15	11.02	2.25E-29	conserved hypothetical protein (RhoGEF domain)	
CIMG_10242	2.48	1.39E-09	2.29	9.12E-06	26S proteasome non-ATPase regulatory subunit Nas2	
CIMG_10264	4.37	5.81E-12	67.65	2.20E-61	aldehyde reductase	**S**
CIMG_10267	1.88	0.00006	2.24	9.84E-06	cytochrome c oxidase assembly protein Cox19	
CIMG_10268	1.58	0.00239	2.01	0.00029	RMD11	
CIMG_10269	3.00	2.09E-08	2.24	0.00073	conserved hypothetical protein	
CIMG_10270	2.06	0.00001	1.80	0.00271	La domain-containing protein	
CIMG_10277	1.54	0.00366	2.17	4.42E-06	mitochondrial inner membrane translocase subunit TIM44	
CIMG_10282	4.52	1.27E-14	8.04	7.86E-24	conserved hypothetical protein	
CIMG_10285	1.80	0.00490	2.19	0.00066	conserved hypothetical protein	
CIMG_10294	1.82	0.00005	2.16	0.00001	conserved hypothetical protein (Fungal Zn(2)-Cys(6) binuclear cluster domain)	
CIMG_10302	1.96	0.00002	2.47	3.98E-06	conserved hypothetical protein	
CIMG_10304	3.04	2.92E-13	7.14	1.71E-27	conserved hypothetical protein	
CIMG_10307	4.66	0.00001	9.63	4.09E-16	hypothetical protein	
CIMG_10317	1.86	0.00003	2.45	3.70E-07	integral membrane protein (Mpv17/PMP22 family)	
CIMG_10318	2.14	0.00058	2.18	0.00129	phosphoglycerate kinase 1	
CIMG_10434	15.70	2.15E-15	5.47	1.83E-09	conserved hypothetical protein	
CIMG_10440	2.24	5.84E-08	2.01	0.00008	conserved hypothetical protein	
CIMG_10463	1.40	0.03164	14.71	1.23E-31	DUF1212 domain membrane protein Prm10	
CIMG_10467	3.48	0.00565	3.20	0.00001	conserved hypothetical protein	
CIMG_10471	2.81	0.00685	3.16	0.00017	hypothetical protein	
CIMG_10478	3.25	1.00E-06	3.88	0.00182	hypothetical protein	
CIMG_10488	11.99	2.36E-15	24.18	6.41E-18	conserved hypothetical protein	
CIMG_10492	4.21	0.00001	2.89	0.00008	hypothetical protein	

*: Predicted secreted;
+: Gene under positive selection [19]; G: Gene gained in Coccididioides [19]
H, S: Up-regulated in Hyphae or Spherule by microarray [18]

Table S2: All genes up-regulated in parasitic phase

CIMG_10494	5.59	1.62E-22	1.93	0.00022	conserved hypothetical protein	
CIMG_10499	5.83	3.21E-16	4.23	5.67E-09	carboxypeptidase S1	*
CIMG_10500	2.06	0.00016	4.44	1.73E-09	hypothetical protein	
CIMG_10503	2.70	9.25E-07	9.53	6.55E-26	RNA binding protein MSSP-2	
CIMG_10505	11.43	1.05E-20	6.12	1.77E-11	hypothetical protein	
CIMG_10508	2.99	0.00001	2.24	0.00393	hypothetical protein	
CIMG_10516	1.54	0.04858	2.41	1.40E-06	hypothetical protein	
CIMG_10521	4.52	1.90E-12	5.55	2.20E-10	hypothetical protein	
CIMG_10537	2.96	1.56E-07	2.09	0.02775	hypothetical protein	
CIMG_10588	3.55	1.01E-11	2.30	6.05E-06	alpha/beta hydrolase	
CIMG_10589	3.36	3.55E-11	3.20	2.34E-07	LYR family protein	
CIMG_10605	1.90	0.00908	2.51	0.01547	hypothetical protein	
CIMG_10608	2.98	0.00002	2.07	0.00630	hypothetical protein	
CIMG_10613	4.23	3.86E-10	1.95	0.00120	conserved hypothetical protein	
CIMG_10628	2.16	0.00006	6.08	2.15E-20	integral membrane protein	
CIMG_10637	7.51	5.72E-20	10.89	3.63E-27	hypothetical protein	
CIMG_10638	2.42	0.01326	2.29	0.00009	hypothetical protein	
CIMG_10641	2.29	0.00610	2.31	0.00092	hypothetical protein	
CIMG_10662	1.83	0.00114	1.81	0.00084	hypothetical protein	
CIMG_10666	1.78	0.00504	4.08	7.76E-13	conserved hypothetical protein	
CIMG_10670	15.39	5.01E-12	20.01	1.95E-20	hypothetical protein	
CIMG_10675	2.02	0.00570	3.75	1.57E-06	hypothetical protein	
CIMG_10682	3.53	9.85E-08	6.12	4.13E-10	hypothetical protein	
CIMG_10697	2.90	4.90E-08	6.07	1.13E-14	conserved hypothetical protein	
CIMG_10704	5.70	1.13E-06	2.21	0.02157	hypothetical protein	
CIMG_10709	7.56	2.79E-10	3.59	0.00002	hypothetical protein	
CIMG_10710	3.24	1.25E-09	2.79	1.26E-07	hypothetical protein	
CIMG_10727	5.14	1.16E-15	2.49	0.00017	hypothetical protein	
CIMG_10730	3.83	1.11E-07	2.18	0.00545	hypothetical protein	
CIMG_10738	1.55	0.03215	3.80	3.30E-09	conserved hypothetical protein	
CIMG_10740	5.68	8.08E-17	8.66	3.60E-11	hypothetical protein	
CIMG_10751	1.78	0.00040	1.67	0.00121	conserved hypothetical protein (putative membrane protein)	
CIMG_10769	4.30	0.00011	5.35	1.06E-07	hypothetical protein	
CIMG_10785	3.33	4.09E-12	4.09	2.73E-12	hypothetical protein	
CIMG_10791	3.20	3.52E-06	6.00	3.06E-06	phosphotransferase enzyme family protein	
CIMG_10796	4.80	2.04E-10	7.47	4.44E-13	hypothetical protein	
CIMG_10799	1.94	0.00002	1.60	0.01950	conserved hypothetical protein	
CIMG_10801	3.12	0.00140	3.21	0.00007	hypothetical protein	
CIMG_10810	1.98	0.00002	2.11	0.00005	conserved hypothetical protein (Ran-interacting Mog1 protein)	
CIMG_10815	4.03	4.03E-09	3.64	8.24E-07	phosphotransferase enzyme family protein	
CIMG_10824	6.05	5.24E-16	4.52	6.27E-08	hypothetical protein	
CIMG_10832	2.22	4.02E-07	1.77	0.00501	acyl-CoA oxidase	
CIMG_10838	3.38	0.00005	6.42	3.75E-09	hypothetical protein	
CIMG_10845	1.61	0.00212	1.51	0.02571	hypothetical protein	
CIMG_10859	2.16	0.00349	2.91	0.00007	hypothetical protein	
CIMG_10861	3.67	3.16E-08	5.09	4.27E-11	hypothetical protein	
CIMG_10866	2.44	0.00920	3.61	0.00229	conserved hypothetical protein	
CIMG_10871	2.07	0.00070	2.59	0.00010	conserved hypothetical protein (TBP-1 interacting protein)	
CIMG_10873	4.10	1.64E-12	2.99	2.82E-08	conserved hypothetical protein	
CIMG_10884	2.46	0.00257	11.05	2.70E-17	hypothetical protein	
CIMG_10887	3.48	7.10E-12	3.62	3.37E-09	conserved hypothetical protein	
CIMG_10891	12.47	5.74E-35	6.25	2.38E-21	hypothetical protein	
CIMG_10901	2.22	0.00586	3.09	0.00009	hypothetical protein	
CIMG_10902	2.47	5.71E-09	2.15	3.98E-06	conserved hypothetical protein	
CIMG_10917	4.05	9.62E-09	3.23	2.42E-07	conserved hypothetical protein	
CIMG_10938	1.90	0.00115	1.84	0.02528	MFS drug efflux transporter	
CIMG_10940	3.33	0.00008	15.75	2.94E-30	hypothetical protein	*
CIMG_10941	1.92	0.00036	5.26	1.02E-10	hypothetical protein	
CIMG_10944	2.55	6.79E-07	4.33	3.67E-14	conserved hypothetical protein	
CIMG_10955	2.97	1.29E-07	2.85	0.00001	hypothetical protein	+
CIMG_10966	10.95	5.07E-14	5.19	1.06E-08	protein phosphatase 2C	
CIMG_10973	2.25	0.00015	3.31	8.46E-07	conserved hypothetical protein	
CIMG_10974	9.93	1.49E-10	8.09	1.15E-08	hypothetical protein	
CIMG_10980	6.14	8.12E-07	4.84	0.00011	hypothetical protein	
CIMG_10997	1.63	0.00139	1.80	0.00268	leucyl-tRNA synthetase	
CIMG_11001	2.55	0.00029	2.85	0.00037	hypothetical protein	
CIMG_11002	1.84	0.00554	2.74	2.85E-07	short chain dehydrogenase/reductase	
CIMG_11008	8.99	8.36E-11	7.01	5.74E-07	hypothetical protein	
CIMG_11011	18.37	2.44E-12	10.99	1.13E-08	hypothetical protein	
CIMG_11036	1.85	0.00005	2.07	0.00010	glutathione S-transferase	
CIMG_11038	5.96	2.57E-20	12.03	1.24E-18	hypothetical protein	
CIMG_11060	9.83	6.50E-08	6.64	7.45E-07	hypothetical protein	
CIMG_11064	3.61	3.84E-16	2.06	7.91E-06	pyridine nucleotide-disulphide oxidoreductase	
CIMG_11069	9.76	1.24E-30	2.80	7.49E-08	hypothetical protein	
CIMG_11081	3.54	8.34E-08	4.26	4.22E-07	hypothetical protein	
CIMG_11087	11.23	4.08E-20	11.85	1.87E-18	hypothetical protein	
CIMG_11094	3.98	1.26E-07	4.76	7.00E-07	hypothetical protein	
CIMG_11098	5.03	0.00006	4.64	0.00017	hypothetical protein	
CIMG_11118	4.66	4.90E-08	4.95	1.91E-07	hypothetical protein	
CIMG_11130	1.66	0.00683	2.47	0.00007	hypothetical protein	

*: Predicted secreted;
+: Gene under positive selection [19]; G: Gene gained in Coccidioides [19]
H, S: Up-regulated in Hyphae or Spherule by microarray [18]

Table S2: All genes up-regulated in parasitic phase

CIMG_11132	3.87	0.00046	4.27	2.55E-06	hypothetical protein	
CIMG_11137	4.88	3.76E-13	3.91	1.07E-07	hypothetical protein	
CIMG_11147	1.36	0.03354	2.17	0.00002	cyclin	
CIMG_11151	3.52	3.86E-08	1.90	0.00951	conserved hypothetical protein	
CIMG_11156	4.75	1.21E-13	3.34	0.00003	conserved hypothetical protein	
CIMG_11166	1.51	0.01337	2.13	0.00019	FHA domain-containing protein SNIP1	
CIMG_11170	1.68	0.00064	5.43	2.35E-23	hypothetical protein	
CIMG_11192	3.65	5.40E-08	4.69	7.79E-07	hypothetical protein	
CIMG_11202	3.57	1.50E-06	4.44	7.51E-09	hypothetical protein	
CIMG_11203	30.77	1.04E-47	15.56	2.20E-36	hypothetical protein	
CIMG_11213	1.97	0.00004	2.81	3.17E-11	integral membrane protein	
CIMG_11218	2.85	8.80E-11	2.01	0.00040	hypothetical protein	*
CIMG_11219	1.53	0.01699	3.51	2.94E-08	hypothetical protein	
CIMG_11220	5.05	1.28E-12	5.20	5.22E-11	conserved hypothetical protein	
CIMG_11224	2.34	0.00468	3.55	0.00959	hypothetical protein	
CIMG_11231	1.80	0.00005	2.37	2.17E-07	tRNA splicing 2' phosphotransferase 1	
CIMG_11244	1.79	0.00078	1.97	0.00069	conserved hypothetical protein	
CIMG_11246	2.50	0.00011	9.32	6.44E-10	hypothetical protein	
CIMG_11251	2.76	0.00071	4.68	7.59E-06	hypothetical protein	
CIMG_11266	2.30	0.03021	2.37	0.04272	hypothetical protein	
CIMG_11290	1.86	0.00212	1.72	0.04540	hypothetical protein	
CIMG_11294	1.81	0.00190	1.55	0.01607	conserved hypothetical protein	
CIMG_11295	3.55	3.24E-14	2.64	1.18E-07	conserved hypothetical protein (Methyltransferase domain)	
CIMG_11297	5.60	2.78E-12	48.13	2.13E-32	hypothetical protein	
CIMG_11301	1.69	0.00200	2.16	0.00025	conserved hypothetical protein (putative glutathione-dependent formaldehyde-activating enzyme)	
CIMG_11309	5.60	2.84E-16	7.04	1.14E-17	beta-N-acetylglucosaminidase	**S**
CIMG_11317	6.53	1.57E-07	8.54	8.55E-09	conserved hypothetical protein	
CIMG_11321	3.00	3.88E-07	2.09	0.00380	hypothetical protein	
CIMG_11323	1.71	0.00016	1.46	0.04898	FYVE zinc finger protein	
CIMG_11324	2.34	0.00220	14.14	5.33E-14	hypothetical protein	
CIMG_11342	4.77	1.30E-13	4.45	7.27E-10	hypothetical protein	
CIMG_11343	2.29	0.03329	6.91	1.28E-06	hypothetical protein	
CIMG_11344	2.04	0.00152	2.61	0.00078	hypothetical protein	
CIMG_11345	3.28	9.90E-07	3.88	0.01286	hypothetical protein	
CIMG_11347	2.08	0.00008	1.98	0.00264	hypothetical protein	
CIMG_11349	3.12	0.00005	7.76	3.42E-08	conserved hypothetical protein	
CIMG_11360	2.76	3.65E-06	2.90	0.00066	conserved hypothetical protein	
CIMG_11374	6.95	5.75E-14	7.02	1.35E-17	conserved hypothetical protein (FAD binding domain, Berberine and berberine like)	*
CIMG_11395	1.55	0.01909	2.35	0.00028	hypothetical protein	
CIMG_11408	3.18	0.00001	8.04	5.77E-11	hypothetical protein	
CIMG_11416	12.14	3.21E-35	2.50	0.00003	conserved hypothetical protein (FAD binding domain, Thi4 family)	
CIMG_11422	1.85	0.00007	1.89	0.00123	C2H2 transcription factor	
CIMG_11436	2.30	0.00541	3.19	0.00007	hypothetical protein (2OG-Fe(II) oxygenase superfamily)	
CIMG_11443	2.90	1.19E-07	2.52	0.00002	hypothetical protein	*
CIMG_11456	3.27	9.16E-15	3.75	4.44E-12	conserved hypothetical protein	
CIMG_11458	1.43	0.01577	1.75	0.00442	alpha/beta hydrolase	
CIMG_11460	1.43	0.01346	5.97	4.10E-20	hypothetical protein	
CIMG_11463	3.51	0.00005	3.11	0.00037	hypothetical protein	
CIMG_11491	3.46	6.47E-08	1.92	0.03933	conserved hypothetical protein (meiosis protein SPO22/ZIP4-like)	
CIMG_11520	16.05	2.23E-41	13.16	2.84E-22	hypothetical protein	
CIMG_11522	10.23	1.16E-26	46.44	1.83E-64	hypothetical protein	
CIMG_11523	7.69	2.28E-30	8.90	1.02E-31	hypothetical protein	
CIMG_11529	2.01	0.00005	3.27	1.17E-08	phospholipase A2	*
CIMG_11534	6.03	2.74E-16	8.03	7.31E-18	hypothetical protein	
CIMG_11535	3.14	2.74E-06	2.94	9.67E-07	hypothetical protein	
CIMG_11536	1.63	0.00341	1.68	0.00278	plasma membrane antiporter	
CIMG_11543	4.42	5.28E-09	9.66	1.24E-22	conserved hypothetical protein	
CIMG_11545	2.27	0.00001	1.53	0.00333	fructose-2,6-bisphosphatase	
CIMG_11550	3.67	1.13E-10	2.21	0.00004	hypothetical protein	
CIMG_11551	10.87	2.87E-09	11.35	9.80E-09	hypothetical protein	
CIMG_11552	2.37	2.35E-08	2.31	3.25E-07	hypothetical protein	
CIMG_11553	3.53	3.98E-08	4.63	4.14E-11	hypothetical protein	
CIMG_11555	4.04	2.86E-13	1.76	0.02951	conserved hypothetical protein	
CIMG_11556	3.98	0.00004	4.80	1.36E-08	hypothetical protein	
CIMG_11557	14.47	7.14E-31	6.16	4.83E-10	hypothetical protein	
CIMG_11558	2.17	4.74E-06	3.91	2.42E-14	conserved hypothetical protein	
CIMG_11561	4.57	3.78E-06	5.54	3.65E-13	sucrase/ferredoxin domain-containing protein	
CIMG_11587	1.82	0.00378	2.34	0.00019	conserved hypothetical protein	
CIMG_11589	1.76	0.01125	2.01	0.00893	conserved hypothetical protein	
CIMG_11604	2.15	2.01E-06	2.02	0.00004	GNAT family acetyltransferase	
CIMG_11607	3.84	1.72E-15	2.47	3.82E-06	hypothetical protein	
CIMG_11625	2.12	0.03156	1.66	0.03000	conserved hypothetical protein (glycosyl hydrolase family 20 catalytic domain)	
CIMG_11639	1.46	0.01479	1.83	0.00013	mitochondrial outer membrane protein	
CIMG_11650	4.39	0.00029	4.36	0.00080	hypothetical protein	
CIMG_11655	6.03	1.78E-19	12.17	6.57E-23	conserved hypothetical protein	
CIMG_11657	2.07	0.00164	4.49	9.21E-11	hypothetical protein	
CIMG_11663	7.33	2.39E-20	2.27	0.00197	hypothetical protein	

*: Predicted secreted;
+: Gene under positive selection [19]; G: Gene gained in Coccidioides [19]
H, S: Up-regulated in Hyphae or Spherule by microarray [18]

Table S2: All genes up-regulated in parasitic phase

CIMG_11664	5.82	1.85E-24	8.64	4.84E-36	hypothetical protein	
CIMG_11682	2.82	1.16E-09	2.96	2.47E-08	inositol 5-phosphatase	
CIMG_11698	4.71	0.00001	6.86	4.52E-20	hypothetical protein	
CIMG_11714	9.41	1.87E-20	1.90	0.02019	hypothetical protein	
CIMG_11718	2.41	8.68E-09	2.16	0.00001	conserved hypothetical protein	
CIMG_11719	3.76	6.65E-12	4.25	4.97E-11	trehalose synthase	**S**
CIMG_11723	2.40	0.00182	7.73	5.26E-24	hypothetical protein	
CIMG_11727	1.58	0.00329	3.33	9.35E-12	NADH-ubiquinone oxidoreductase 78 kDa subunit	
CIMG_11728	6.79	1.49E-19	9.04	1.33E-19	hypothetical protein	
CIMG_11738	2.30	1.41E-08	1.43	0.00363	MATH and UCH domain-containing protein	
CIMG_11742	6.68	5.19E-22	4.92	8.47E-13	hypothetical protein	
CIMG_11755	2.33	0.00001	3.07	2.65E-08	conserved hypothetical protein	
CIMG_11766	2.32	0.00053	7.73	1.37E-16	hypothetical protein	*
CIMG_11791	4.44	2.38E-07	5.14	0.00012	hypothetical protein	
CIMG_11800	1.53	0.01659	1.54	0.04899	metalloproteinase 5	*
CIMG_11809	2.13	6.51E-07	8.77	3.58E-20	sister chromatid cohesion protein	
CIMG_11845	1.85	0.00018	1.65	0.01268	RNA binding protein	
CIMG_11847	3.62	6.49E-10	10.05	5.46E-17	hypothetical protein	
CIMG_11875	2.00	0.00416	3.22	7.68E-06	conserved hypothetical protein	*
CIMG_11895	1.76	0.00009	4.84	2.85E-16	conserved hypothetical protein	*
CIMG_11896	2.86	2.36E-07	2.06	0.01034	conserved hypothetical protein (Beta-lactamase)	
CIMG_11899	5.56	5.19E-21	2.39	9.44E-06	conserved hypothetical protein	
CIMG_11902	3.78	7.33E-09	2.09	0.01421	hypothetical protein	
CIMG_11913	1.78	0.00250	2.32	0.00088	hypothetical protein	
CIMG_11915	1.50	0.00617	1.47	0.01156	WD repeat protein	
CIMG_11931	4.69	1.65E-21	3.53	5.92E-12	FK506 suppressor Sfk1	*
CIMG_11944	1.47	0.04970	2.35	0.00068	hypothetical protein	
CIMG_11953	2.78	0.00011	2.53	0.00378	hypothetical protein	
CIMG_11986	4.00	0.00006	2.47	0.00016	hypothetical protein	
CIMG_12013	3.16	6.55E-13	3.32	1.67E-10	conserved hypothetical protein	
CIMG_12018	3.77	3.48E-14	1.90	0.00335	ubiE/COQ5 methyltransferase	
CIMG_12027	7.11	7.05E-12	14.41	1.33E-21	hypothetical protein	*
CIMG_12045	3.91	0.00003	4.61	0.02002	hypothetical protein	
CIMG_12050	3.23	2.91E-12	1.60	0.00914	hypothetical protein	
CIMG_12068	7.56	1.08E-27	5.67	1.19E-22	multicopper oxidase	*
CIMG_12069	1.57	0.00256	2.84	2.14E-10	long-chain fatty acid transporter	*
CIMG_12088	3.38	3.22E-10	4.34	1.42E-11	hypothetical protein	
CIMG_12089	3.59	3.86E-08	5.38	5.37E-06	hypothetical protein	
CIMG_12111	2.00	0.00001	1.41	0.03718	DNA damage-inducible protein 1	
CIMG_12142	6.53	1.04E-20	2.66	0.00001	hypothetical protein	
CIMG_12143	8.89	5.83E-09	8.62	5.31E-10	hypothetical protein	
CIMG_12198	1.96	0.03954	2.08	0.00431	conserved hypothetical protein	
CIMG_12203	6.63	1.54E-25	7.11	3.99E-33	hypothetical protein	
CIMG_12204	2.88	5.07E-11	3.63	3.81E-14	hypothetical protein	
CIMG_12244	3.35	2.23E-06	2.16	0.00135	conserved hypothetical protein	
CIMG_12252	3.53	0.00003	6.63	0.00001	hypothetical protein	
CIMG_12263	2.99	0.00038	2.49	0.01304	hypothetical protein	
CIMG_12264	3.28	9.34E-08	4.02	1.65E-07	hypothetical protein	
CIMG_12272	6.34	2.07E-21	12.28	1.09E-32	hypothetical protein	
CIMG_12285	2.76	5.86E-12	4.44	8.44E-08	hypothetical protein	
CIMG_12292	2.13	4.79E-06	1.38	0.03563	AP-3 adaptor complex subunit beta	
CIMG_12303	1.87	0.00069	1.58	0.01024	phthalate transporter	
CIMG_12310	7.36	5.94E-25	4.87	2.35E-12	hypothetical protein	
CIMG_12314	3.46	1.30E-08	1.90	0.01807	hypothetical protein	*
CIMG_12341	9.95	5.05E-22	4.84	1.37E-08	hypothetical protein	
CIMG_12343	1.52	0.03100	1.86	0.00996	conserved hypothetical protein (zinc-binding dehydrogenase)	
CIMG_12358	11.35	1.60E-37	13.09	2.53E-18	hypothetical protein	
CIMG_12361	4.32	6.45E-20	1.56	0.04188	conserved hypothetical protein (GDP/GTP exchange factor Sec2p)	
CIMG_12383	2.44	0.00176	2.44	0.00013	hypothetical protein	
CIMG_12387	13.56	1.18E-34	12.05	5.79E-20	hypothetical protein	
CIMG_12388	3.75	2.08E-11	2.83	8.72E-07	hypothetical protein	
CIMG_12390	4.41	0.00098	4.38	8.79E-06	hypothetical protein	
CIMG_12394	7.85	6.00E-11	4.98	6.84E-08	hypothetical protein	
CIMG_12395	2.07	1.92E-06	2.59	9.10E-07	copper amine oxidase	
CIMG_12396	4.25	2.94E-20	10.15	8.95E-34	UV-endonuclease UVE-1	
CIMG_12404	2.54	9.76E-07	2.68	0.00002	conserved hypothetical protein	**S**
CIMG_12411	1.76	0.00023	5.72	5.24E-25	acetamidase	
CIMG_12424	3.86	6.75E-16	3.97	2.98E-10	hypothetical protein	*
CIMG_12427	1.54	0.01659	4.33	9.65E-12	hypothetical protein	
CIMG_12441	2.46	0.00021	3.78	7.29E-07	hypothetical protein	
CIMG_12456	3.31	6.64E-10	2.18	0.00016	hypothetical protein	
CIMG_12470	2.47	0.00037	2.64	0.00167	hypothetical protein	
CIMG_12472	2.67	9.39E-10	7.73	4.89E-20	conserved hypothetical protein (SCP-like extracellular protein)	*
CIMG_12485	2.21	0.03726	13.63	5.22E-16	hypothetical protein	
CIMG_12499	2.82	0.00824	7.58	2.11E-11	hypothetical protein	
CIMG_12501	3.33	0.00005	7.42	2.10E-15	conserved hypothetical protein	
CIMG_12513	1.51	0.01197	2.18	0.00017	hypothetical protein	
CIMG_12515	2.04	0.00788	7.48	1.38E-14	hypothetical protein	
CIMG_12529	2.01	3.44E-06	2.63	1.25E-07	conserved hypothetical protein	
CIMG_12534	1.77	0.00140	6.32	7.97E-23	conserved hypothetical protein	

*: Predicted secreted;
+: Gene under positive selection [19]; G: Gene gained in Coccidioides [19]
H, S: Up-regulated in Hyphae or Spherule by microarray [18]

Table S2: All genes up-regulated in parasitic phase

CIMG_12535	3.24	3.25E-10	1.99	0.00561	conserved hypothetical protein	
CIMG_12537	6.56	5.22E-09	3.40	0.00359	hypothetical protein	
CIMG_12539	2.01	2.52E-06	3.56	4.63E-13	DUF636 domain-containing protein	
CIMG_12569	17.87	1.63E-11	6.79	1.11E-19	C2H2 finger domain-containing protein	
CIMG_12621	2.60	0.00003	2.32	0.00028	hypothetical protein	
CIMG_12635	15.36	2.22E-09	1.80	0.00006	conserved hypothetical protein (protein kinase domain)	
CIMG_12643	2.40	8.92E-10	4.36	1.22E-13	hypothetical protein	
CIMG_12656	1.80	0.02208	3.17	6.01E-06	conserved hypothetical protein	
CIMG_12659	2.21	0.00319	2.15	0.00810	hypothetical protein	
CIMG_12670	1.46	0.03016	2.37	3.43E-06	hypothetical protein	
CIMG_12671	2.86	0.00396	4.32	0.00003	conserved hypothetical protein	*
CIMG_12690	6.76	1.58E-09	3.33	0.00007	conserved hypothetical protein	
CIMG_12707	2.45	2.09E-08	2.98	6.74E-08	conserved hypothetical protein	
CIMG_12714	2.46	4.23E-10	2.54	0.00002	conserved hypothetical protein	
CIMG_12724	2.56	5.38E-07	2.20	5.91E-06	hypothetical protein	
CIMG_12727	2.21	0.02098	2.90	0.01902	conserved hypothetical protein	
CIMG_12738	3.30	7.05E-12	2.27	0.00088	conserved hypothetical protein	
CIMG_12739	1.82	0.04597	3.14	0.00185	conserved hypothetical protein	
CIMG_12741	2.54	1.49E-06	3.34	5.62E-09	hypothetical protein	
CIMG_12745	3.34	1.15E-06	3.50	1.07E-06	hypothetical protein	
CIMG_12746	2.66	0.00006	2.26	0.02138	conserved hypothetical protein	
CIMG_12747	6.93	8.08E-14	23.12	3.40E-27	hypothetical protein	
CIMG_12749	2.92	1.10E-07	2.06	0.00145	conserved hypothetical protein	
CIMG_12752	2.85	3.77E-07	4.72	1.54E-08	conserved hypothetical protein	*
CIMG_12758	3.17	9.77E-09	3.41	1.96E-09	FluG domain-containing protein	
CIMG_12760	1.48	0.00795	2.60	5.09E-08	conserved hypothetical protein	
CIMG_12761	1.31	0.04198	16.38	4.66E-48	conserved hypothetical protein (putative heat shock protein 9/12)	
CIMG_12764	7.23	6.40E-08	4.06	0.00174	hypothetical protein	
CIMG_12770	2.14	6.74E-08	4.61	6.01E-19	conserved hypothetical protein	
CIMG_12774	1.77	0.00241	2.66	0.00004	hypothetical protein	
CIMG_12778	3.19	5.78E-08	2.04	0.00264	conserved hypothetical protein	
CIMG_12779	2.04	0.00045	3.59	2.14E-09	hypothetical protein	
CIMG_12782	4.69	3.72E-21	2.45	0.00059	conserved hypothetical protein	
CIMG_12786	3.92	1.94E-06	1.85	0.03832	hypothetical protein	
CIMG_12788	3.20	0.00351	3.38	0.00002	hypothetical protein	
CIMG_12789	5.12	8.42E-19	2.62	0.00009	conserved hypothetical protein	
CIMG_12791	3.31	0.00005	4.25	0.00003	conserved hypothetical protein	
CIMG_12795	2.32	0.03113	5.01	1.84E-06	conserved hypothetical protein	
CIMG_12798	3.47	0.00002	5.09	2.27E-06	conserved hypothetical protein	
CIMG_12802	4.52	3.80E-16	2.58	0.00028	conserved hypothetical protein	
CIMG_12807	2.20	0.00089	5.61	1.19E-10	hypothetical protein	
CIMG_12812	3.22	4.80E-14	25.90	1.40E-53	conserved hypothetical protein	
CIMG_12822	45.40	1.34E-60	16.71	5.54E-45	conserved hypothetical protein	
CIMG_12831	6.08	2.41E-20	6.36	2.13E-19	conserved hypothetical protein	
CIMG_12835	2.21	0.00468	5.29	6.12E-10	hypothetical protein	
CIMG_12837	1.69	0.01382	2.58	0.00395	hypothetical protein	
CIMG_12839	1.74	0.00991	2.22	0.01607	conserved hypothetical protein	
CIMG_12841	2.59	7.62E-07	3.17	0.00002	hypothetical protein	
CIMG_12852	4.80	7.60E-14	1.99	0.00884	conserved hypothetical protein	
CIMG_12862	3.75	3.09E-11	2.99	0.00019	hypothetical protein	
CIMG_12868	3.93	3.80E-06	2.77	0.00406	hypothetical protein	
CIMG_12869	1.60	0.00106	1.90	0.00002	hypothetical protein	
CIMG_12877	6.16	2.32E-10	2.61	0.00001	hypothetical protein	
CIMG_12879	5.16	1.24E-06	6.06	7.79E-07	conserved hypothetical protein	
CIMG_12880	2.59	0.00001	2.83	0.00003	conserved hypothetical protein	
CIMG_12886	5.43	5.31E-09	4.12	0.00007	hypothetical protein	
CIMG_12887	6.01	3.03E-24	9.01	3.90E-31	glutathione S-transferase omega-like 2	
CIMG_12892	6.50	4.64E-06	5.55	8.48E-08	hypothetical protein	*
CIMG_12894	5.04	4.20E-08	4.04	1.38E-07	conserved hypothetical protein	
CIMG_12895	3.10	2.30E-06	4.93	2.31E-08	conserved hypothetical protein	
CIMG_12898	2.87	6.44E-08	2.67	4.71E-06	hypothetical protein	
CIMG_12903	3.28	8.13E-07	8.22	2.88E-13	hypothetical protein	*
CIMG_12904	2.07	0.00990	2.47	0.00005	hypothetical protein	
CIMG_12905	2.85	5.50E-07	3.23	1.55E-07	conserved hypothetical protein	*
CIMG_12906	2.74	1.12E-07	30.55	2.76E-30	hypothetical protein	
CIMG_12946	4.30	0.00001	4.43	0.00006	hypothetical protein	
CIMG_12963	2.53	3.82E-09	1.73	0.00311	vacuolar protein sorting protein	
CIMG_12964	1.54	0.04794	2.19	0.00099	hypothetical protein	
CIMG_12965	2.08	0.00126	2.84	0.00004	hypothetical protein	
CIMG_12966	5.45	0.00002	1.98	0.02605	conserved hypothetical protein	
CIMG_12968	3.39	2.99E-12	2.66	3.34E-08	conserved hypothetical protein	
CIMG_12969	3.84	1.32E-11	2.19	0.00012	conserved hypothetical protein	
CIMG_12973	1.66	0.04195	2.18	0.02786	hypothetical protein	
CIMG_12974	5.16	3.73E-09	6.57	3.73E-08	hypothetical protein	
CIMG_12980	2.42	0.00599	2.07	0.00916	conserved hypothetical protein	
CIMG_12981	1.66	0.00056	5.76	8.16E-21	conserved hypothetical protein (methyl-CpG binding domain-containing protein 4)	
CIMG_12987	69.58	2.14E-32	5.76	6.37E-06	conserved hypothetical protein	
CIMG_12992	1.97	0.00586	1.93	0.01510	hypothetical protein	
CIMG_13013	1.82	0.00407	3.51	0.00001	hypothetical protein	

*: Predicted secreted;
+: Gene under positive selection [19]; G: Gene gained in Coccidioides [19]
H, S: Up-regulated in Hyphae or Spherule by microarray [18]

Table S2: All genes up-regulated in parasitic phase

CIMG_13014	3.50	0.00002	3.32	0.00151	hypothetical protein	
CIMG_13015	2.68	8.23E-07	2.21	0.00033	hypothetical protein	
CIMG_13018	5.96	0.00005	2.84	0.04708	conserved hypothetical protein	
CIMG_13019	2.45	1.94E-07	1.65	0.00902	hypothetical protein (C2H2 type zinc finger)	
CIMG_13022	6.82	5.99E-09	3.81	0.00007	hypothetical protein	
CIMG_13025	5.50	2.24E-08	9.01	1.60E-09	hypothetical protein	
CIMG_13031	1.69	0.02658	2.49	0.00333	hypothetical protein	
CIMG_13049	2.66	0.00003	6.49	3.55E-12	hypothetical protein	
CIMG_13061	3.03	6.87E-07	2.74	0.00015	hypothetical protein	
CIMG_13079	3.08	7.96E-07	2.06	0.00081	conserved hypothetical protein (DUF1037)	
CIMG_13082	6.29	3.65E-16	7.95	2.39E-16	hypothetical protein	*
CIMG_13083	3.40	2.38E-07	4.71	4.40E-09	hypothetical protein	
CIMG_13084	27.58	1.10E-50	30.90	8.17E-47	conserved hypothetical protein	
CIMG_13085	3.63	3.58E-13	4.25	2.82E-17	hypothetical protein	
CIMG_13092	4.14	6.03E-18	4.55	4.97E-14	succinyl-CoA ligase, alpha-chain (SUCLG1)	
CIMG_13098	1.36	0.02417	2.72	7.29E-09	hypothetical protein	
CIMG_13110	6.10	1.70E-23	3.26	1.95E-10	conserved hypothetical protein	
CIMG_13111	5.26	3.36E-14	4.91	1.18E-11	hypothetical protein	
CIMG_13119	3.69	1.34E-11	2.15	0.01051	hypothetical protein	
CIMG_13121	3.70	1.38E-07	2.80	0.00057	hypothetical protein	
CIMG_13134	1.93	3.51E-06	2.73	6.48E-12	hypothetical protein	
CIMG_13136	9.29	2.04E-11	7.21	1.72E-09	conserved hypothetical protein	
CIMG_13137	2.04	0.00382	2.27	0.01051	conserved hypothetical protein	
CIMG_13160	13.30	2.46E-36	1.76	0.00139	C6 zinc finger domain-containing protein	
CIMG_13163	4.04	1.36E-09	3.90	4.37E-09	conserved hypothetical protein (putative DNA mismatch repair protein)	
CIMG_13179	1.90	0.01862	4.03	0.00002	hypothetical protein	
CIMG_13180	8.51	1.29E-11	2.89	0.00010	conserved hypothetical protein	
CIMG_13183	2.21	0.00239	2.49	0.00484	conserved hypothetical protein	
CIMG_13191	2.52	0.00052	4.78	2.24E-07	conserved hypothetical protein	
CIMG_13199	11.43	7.54E-19	7.01	1.67E-10	hypothetical protein	
CIMG_13254	3.31	0.00001	1.63	0.01062	hypothetical protein	
CIMG_13255	2.25	4.11E-06	1.99	0.00002	3-isopropylmalate dehydratase	
CIMG_13256	1.93	0.00004	1.60	0.01751	alpha-1,3-glucan synthase	*
CIMG_13257	4.40	2.45E-20	5.27	1.25E-20	hypothetical protein	
CIMG_13258	2.80	0.00202	4.02	0.00002	hypothetical protein	
CIMG_13259	3.96	7.31E-07	7.61	3.54E-08	hypothetical protein	*
CIMG_13263	5.66	0.00001	3.01	0.01197	hypothetical protein	
CIMG_13264	2.37	0.00027	1.77	0.00999	hypothetical protein	
CIMG_13271	3.44	2.23E-07	8.21	2.14E-14	conserved hypothetical protein	
CIMG_13273	6.91	8.44E-19	8.47	1.33E-15	conserved hypothetical protein	
CIMG_13280	5.04	1.39E-19	7.05	4.62E-18	hypothetical protein	
CIMG_13281	2.74	0.00001	3.01	0.00024	hypothetical protein	
CIMG_13282	3.17	9.94E-07	4.60	4.87E-10	hypothetical protein	
CIMG_13283	3.15	0.00052	8.23	3.31E-13	hypothetical protein	
CIMG_13286	4.19	2.82E-11	3.57	1.66E-09	conserved hypothetical protein	
CIMG_13287	3.90	1.54E-07	2.57	0.00965	conserved hypothetical protein	
CIMG_13291	1.68	0.02558	1.94	0.04075	conserved hypothetical protein	
CIMG_13292	1.79	0.02072	1.97	0.03060	hypothetical protein	
CIMG_13293	2.83	1.01E-09	5.02	1.08E-15	hypothetical protein	*
CIMG_13294	3.50	0.00005	3.27	0.00003	hypothetical protein	
CIMG_13295	1.84	0.00383	2.28	0.00017	glycosyl hydrolase	
CIMG_13311	3.91	2.52E-14	2.56	5.41E-06	hypothetical protein	
CIMG_13313	8.14	7.14E-07	6.39	0.00004	hypothetical protein	
CIMG_13314	10.91	3.43E-09	7.12	6.06E-07	hypothetical protein	
CIMG_13316	6.45	1.10E-21	16.37	1.71E-36	hypothetical protein	
CIMG_13320	2.53	0.00011	2.57	0.00947	hypothetical protein	
CIMG_13321	6.13	5.95E-11	6.89	7.02E-12	conserved hypothetical protein	
CIMG_13323	5.29	3.05E-06	3.27	0.00620	hypothetical protein	
CIMG_13324	4.12	5.34E-08	3.81	0.00001	hypothetical protein	
CIMG_13325	2.15	0.00075	5.09	3.91E-10	hypothetical protein	
CIMG_13329	1.87	0.00042	1.85	0.00937	hypothetical protein	*
CIMG_13330	4.66	3.58E-07	6.42	8.74E-07	hypothetical protein	
CIMG_13334	2.28	9.19E-08	2.09	0.00003	conserved hypothetical protein	
CIMG_13354	5.30	4.35E-13	5.14	1.71E-09	hypothetical protein	
CIMG_13357	6.64	3.49E-25	2.75	1.69E-08	conserved hypothetical protein	
CIMG_13358	4.22	2.84E-15	6.07	1.75E-21	conserved hypothetical protein	
CIMG_13360	7.29	7.94E-28	69.94	7.29E-83	conserved hypothetical protein	
CIMG_13362	2.99	0.00013	3.76	6.05E-06	hypothetical protein	
CIMG_13363	3.49	0.00021	9.80	2.46E-07	conserved hypothetical protein	
CIMG_13365	2.15	0.00195	7.72	4.67E-17	hypothetical protein	
CIMG_13367	3.34	0.00004	8.10	1.22E-06	hypothetical protein	
CIMG_13370	2.95	0.00002	1.85	0.02119	conserved hypothetical protein (Fungal Zn(2)-Cys(6) binuclear cluster domain, homeobox-associated leucine zipper)	
CIMG_13372	2.15	0.04836	5.29	6.11E-06	conserved hypothetical protein	
CIMG_13380	1.99	0.03468	3.46	3.14E-06	hypothetical protein	
CIMG_13384	13.81	1.19E-41	9.57	7.80E-22	hypothetical protein	
CIMG_13388	5.60	2.65E-18	7.64	6.83E-17	hypothetical protein	
CIMG_13389	13.94	2.11E-10	5.62	1.75E-07	hypothetical protein	
CIMG_13395	4.22	4.91E-14	2.10	0.00419	hypothetical protein	
CIMG_13396	2.10	0.00269	4.75	1.67E-08	hypothetical protein	

*: Predicted secreted;
+: Gene under positive selection [19]; G: Gene gained in Coccidioides [19]
H, S: Up-regulated in Hyphae or Spherule by microarray [18]

Table S2: All genes up-regulated in parasitic phase

CIMG_13398	3.71	0.00001	1.77	0.02814	hypothetical protein	
CIMG_13402	3.13	2.17E-10	2.13	0.00002	conserved hypothetical protein	
CIMG_13407	2.84	3.87E-06	2.04	0.00529	hypothetical protein	
CIMG_13408	3.14	0.00002	2.28	0.00315	hypothetical protein	
CIMG_13411	5.72	1.40E-18	2.85	0.00011	conserved hypothetical protein	
CIMG_13414	3.28	0.00002	6.25	1.36E-09	conserved hypothetical protein	
CIMG_13417	5.34	5.27E-09	5.45	2.26E-06	conserved hypothetical protein	
CIMG_13420	4.79	0.00191	6.59	0.00003	conserved hypothetical protein	
CIMG_13421	4.47	5.51E-10	7.96	5.68E-15	conserved hypothetical protein	
CIMG_13444	4.71	0.00001	3.44	3.66E-07	conserved hypothetical protein	
CIMG_13445	2.63	1.25E-08	4.40	1.08E-15	hypothetical protein	
CIMG_13464	1.76	0.00018	1.71	0.00355	cAMP-dependent protein kinase type 2	
CIMG_13473	1.65	0.00104	2.12	0.00064	YjgH family protein	
CIMG_13476	3.62	7.40E-14	2.69	1.24E-06	MFS monosaccharide transporter	
CIMG_13478	2.56	2.42E-11	2.20	0.00001	hypothetical protein	
CIMG_13497	21.11	2.88E-13	8.10	5.66E-09	conserved hypothetical protein	
CIMG_13502	3.03	4.49E-07	2.99	1.89E-07	hypothetical protein	
CIMG_13504	5.66	4.15E-12	10.08	4.60E-24	hypothetical protein	
CIMG_13526	2.26	0.00012	2.37	0.00020	molybdenum cofactor biosynthetic protein	
CIMG_13529	2.50	0.02437	5.84	4.07E-09	conserved hypothetical protein	
CIMG_13531	8.03	4.36E-27	6.37	6.84E-14	conserved hypothetical protein	
CIMG_13532	2.81	0.00078	2.42	0.03354	hypothetical protein	
CIMG_13533	3.89	0.00001	4.95	4.49E-07	conserved hypothetical protein	
CIMG_13535	2.65	0.00171	7.57	4.80E-13	conserved hypothetical protein	
CIMG_13551	2.25	0.00122	10.99	4.26E-19	conserved hypothetical protein	
CIMG_13569	2.49	0.00005	1.99	0.00145	hypothetical protein	
CIMG_13570	1.98	0.00703	1.91	0.00872	hypothetical protein	
CIMG_13571	2.57	0.00001	8.36	2.65E-16	hypothetical protein	
CIMG_13572	4.11	3.91E-06	18.65	2.15E-51	conserved hypothetical protein (CVNH domain)	*
CIMG_13573	12.78	1.49E-26	10.66	2.67E-19	hypothetical protein	
CIMG_13575	4.07	9.24E-17	1.57	0.00469	conserved hypothetical protein	
CIMG_13576	3.32	0.00034	5.20	0.00002	hypothetical protein	
CIMG_13581	26.29	7.78E-52	11.52	1.38E-20	hypothetical protein	
CIMG_13582	6.60	4.76E-14	7.43	5.74E-12	hypothetical protein	
CIMG_13586	1.62	0.01619	2.98	1.74E-06	conserved hypothetical protein	
CIMG_13590	2.15	0.00011	1.96	0.00981	conserved hypothetical protein (fungal specific transcription factor domain)	
CIMG_13592	1.77	0.00014	2.21	0.00001	pseudouridine synthase	
CIMG_13600	4.09	1.15E-07	2.37	0.01674	hypothetical protein	
CIMG_13603	5.66	2.19E-09	5.01	9.24E-09	conserved hypothetical protein	
CIMG_13604	5.18	4.67E-09	4.60	1.18E-06	hypothetical protein	
CIMG_13605	4.28	0.00022	5.71	1.61E-08	hypothetical protein	
CIMG_13611	3.08	0.00003	2.29	0.00081	conserved hypothetical protein	
CIMG_13613	2.38	0.02785	3.90	0.00072	conserved hypothetical protein	
CIMG_13614	3.08	0.00001	2.21	0.02179	hypothetical protein	
CIMG_13616	2.56	0.00005	3.44	1.40E-06	hypothetical protein	
CIMG_13617	3.00	8.14E-11	2.06	0.00001	conserved hypothetical protein	
CIMG_13619	2.33	0.03800	3.20	2.74E-06	conserved hypothetical protein	
CIMG_13620	3.63	3.54E-06	2.95	0.00023	hypothetical protein	
CIMG_13631	2.54	0.02811	4.63	0.00002	hypothetical protein	
CIMG_13674	2.23	0.00060	2.50	0.00003	hypothetical protein	
CIMG_13679	2.51	0.00001	3.15	5.79E-09	hypothetical protein	
CIMG_13680	9.44	3.70E-14	3.86	7.68E-08	conserved hypothetical protein	
CIMG_13682	3.84	8.46E-10	4.99	4.91E-07	hypothetical protein	
CIMG_13687	2.16	0.00032	4.18	3.30E-14	hypothetical protein	
CIMG_13690	2.00	0.00158	2.01	0.01066	hypothetical protein	
CIMG_13693	1.51	0.02358	2.05	0.00085	tRNA(His) guanylyltransferase	
CIMG_13696	9.75	7.92E-19	21.20	5.41E-35	hypothetical protein	
CIMG_13698	3.92	6.63E-13	6.55	9.65E-27	conserved hypothetical protein	
CIMG_13701	4.15	0.00010	6.51	1.01E-08	hypothetical protein	
CIMG_13703	6.74	3.18E-06	3.53	0.00007	conserved hypothetical protein	
CIMG_13706	2.63	4.18E-10	2.14	3.77E-06	alpha-1,2-mannosyltransferase	S
CIMG_13708	2.52	1.04E-09	27.12	6.77E-64	conserved hypothetical protein	
CIMG_13712	4.11	0.00002	5.13	0.00003	conserved hypothetical protein	
CIMG_13718	1.55	0.02192	2.25	0.00100	hypothetical protein	
CIMG_13721	4.33	1.02E-08	2.39	0.00266	hypothetical protein	*
CIMG_13724	4.23	2.19E-07	4.01	0.00004	hypothetical protein	
CIMG_13726	3.34	4.45E-08	1.91	0.01633	hypothetical protein	
CIMG_13729	2.68	0.00128	2.58	0.00288	hypothetical protein	*
CIMG_13733	2.94	4.29E-06	11.71	8.32E-11	hypothetical protein	
CIMG_13740	6.21	3.02E-15	1.99	0.00180	hypothetical protein	
CIMG_13741	5.92	1.92E-09	6.63	6.76E-14	conserved hypothetical protein	
CIMG_13743	8.91	7.08E-08	5.54	9.56E-07	hypothetical protein	
CIMG_13746	4.72	0.00001	5.21	0.00001	conserved hypothetical protein	
CIMG_13748	6.62	8.43E-14	6.36	8.55E-13	hypothetical protein	
CIMG_13752	2.33	0.00050	5.00	6.34E-09	hypothetical protein	
CIMG_13757	7.88	2.14E-20	16.19	8.45E-42	hypothetical protein	
CIMG_13758	3.60	0.00001	3.60	0.00002	hypothetical protein	
CIMG_13763	2.08	0.01252	2.50	0.00062	hypothetical protein	
CIMG_13766	2.45	1.54E-06	3.11	8.22E-10	glutamate/Leucine/Phenylalanine/Valine dehydrogenase	
CIMG_13767	5.64	9.84E-21	4.51	1.28E-12	hypothetical protein	

*: Predicted secreted;
+: Gene under positive selection [19]; G: Gene gained in Coccididioides [19]
H, S: Up-regulated in Hyphae or Spherule by microarray [18]

Table S2: All genes up-regulated in parasitic phase

CIMG_13768	2.45	0.00003	2.11	0.01732	conserved hypothetical protein	
CIMG_13770	1.98	0.00032	2.88	1.46E-07	dihydroceramide delta	
CIMG_13780	1.84	0.00641	1.79	0.01017	conserved hypothetical protein	

*: Predicted secreted;
+: Gene under positive selection [19]; G: Gene gained in Coccididioides [19]
H, S: Up-regulated in Hyphae or Spherule by microarray [18]
