# Supplementary material for: Comparative Transcriptomics of the Saprobic and Parasitic Growth Phases in Coccidioides spp
Source: PLoS One. 2012 Jul 20;7(7):e41034. doi: 10.1371/journal.pone.0041034 (PMC3401177; doi:10.1371/journal.pone.0041034)
Supplement: Table S3 — Gene ontology (GO) terms significantly enriched (p-value <0.05) in saprobic-phase and parasitic-phase up-regulated gene sets. (DOCX) [file pone.0041034.s003.docx]

| **Phase** | **GO term description** | **Population frequency** | **Sample frequency** |
| --- | --- | --- | --- |
| **Genes up-regulated in saprobic phase** | Inner kinetochore of condensed  chromosome | 10/3857 | 6/610 |
|  | Spindle pole body | 51/3857 | 23/610 |
|  | Cytokinesis | 64/3857 | 22/610 |
|  | Mitotic sister chromatid segregation | 35/3857 | 16/610 |
|  | Attachment of spindle microtubules to  kinetochore | 11/3857 | 7/610 |
|  | Mitotic chromosome condensation | 10/3857 | 8/610 |
|  | Regulation of exit from mitosis | 14/3857 | 9/610 |
|  | Regulation of mitotic cell cycle | 50/3857 | 17/610 |
|  | Nuclear pore organization and biogenesis | 13/3857 | 7/610 |
|  | Nuclear nucleosome | 7/3857 | 5/610 |
|  | rRNA-nucleus export | 13/3857 | 7/610 |
|  | Nucleocytoplasmic transport | 33/3857 | 14/610 |
|  | Bud neck | 50/3857 | 19/610 |
|  | Septin complex | 4/3857 | 4/610 |
|  | Septin ring (sensu Saccharomyces) | 6/3857 | 5/610 |
|  | Cytoplasmic microtubule | 10/3857 | 6/610 |
|  | Structural constituent of cytoskeleton | 23/3857 | 10/610 |
|  | Microtubule cytoskeleton | 17/3857 | 9/610 |
|  | Establishment of cell polarity (sensu  Fungi) | 35/3857 | 15/610 |
|  | Conjugation with cellular fusion | 35/3857 | 13/610 |
|  | Calmodulin binding | 7/3857 | 5/610 |
|  | Isoprenoid biosynthesis | 7/3857 | 5/610 |
|  | Ergosterol biosynthesis | 28/3857 | 12/610 |
|  | Peroxisomal membrane | 11/3857 | 7/610 |
| **Genes up-regulated in parasitic phase** | Response to light | 190/3857 | 53/551 |
|  | Lipid particle | 20/3857 | 11/551 |
|  | Oxidoreductase activity | 36/3857 | 15/551 |

**Table S3**. GO terms significantly enriched (*p*-value < 0.05) in saprobic-phase and parasitic-phase up-regulated gene sets.
